# Chemotherapeutic drug-triggered AEP-cleaved G3BP1 orchestrates stress granules/nucleoli/mitochondria in osteosarcoma

**DOI:** 10.1038/s41413-025-00453-w

**Published:** 2025-08-26

**Authors:** Zhonggang Shi, Jianyi Zhao, Qi Lv, Keman Liao, Lu Cao, Jian Yang, Mengying Wang, Li Zhou, Haoping Xu, Jianwei Ge, Yongming Qiu, Juxiang Chen, Jiayi Chen, Chunhui Ma, Yingying Lin

**Affiliations:** 1 Department of Radiation Oncology, Ruijin Hospital, Shanghai Jiao Tong University School of Medicine, Shanghai, China; 2 Shanghai Key Laboratory of Proton-Therapy, Shanghai, China; 3 Department of Medical Imaging, Tongji Hospital, Tongji University School of Medicine, Shanghai, China; 4 Department of Neurosurgery, Ren Ji Hospital, Shanghai Jiao Tong University School of Medicine, Shanghai, China; 5 Brain Injury Center, Shanghai Institute of Head Trauma, Ren Ji Hospital, Shanghai Jiao Tong University School of Medicine, Shanghai, China; 6 Department of Neurosurgery, Shanghai Changhai Hospital, Naval Medical University, Shanghai, China; 7 Department of Orthopedic Surgery, Shanghai Geriatric Medical Center, Shanghai, China

**Keywords:** Bone cancer, Bone cancer, Pathogenesis

## Abstract

Osteosarcoma (OS) is the most frequent primary bone sarcomas with high recurrence and poor prognosis. Emerging evidence indicates that membraneless organelles stress granules (SGs), whose assemblies are driven by scaffold protein G3BP1, are extensively involved in tumor, especially in OS. However, how SGs behave and communicate with organelles, particularly nucleoli and mitochondria, during drug challenges remain unknown. This study revealed that chemotherapeutic drugs activated the cysteine protease asparagine endopeptidase (AEP) to specifically cleave the SG core protein G3BP1 at N258/N309 in OS and malignant glioma. tG3BP1-Ns modulated SG dynamics by competitively binding to full-length G3BP1. Strikingly, tG3BP1-Cs, containing a conserved RNA recognition motif CCUBSCUS, sequestered mRNAs of ribosomal proteins and oxidative phosphorylation genes in the nucleoli and mitochondria to repress translation and oxidative stress. Moreover, the inhibition of AEP promoted the tumor-suppressing effect of chemotherapeutic drugs, whereas AEP-cleaved G3BP1 rescue reversed the effect in both OS and glioma models. Cancerous tissues exhibited high levels of AEP and G3BP1 truncations, which were strongly associated with poor prognosis. Accordingly, this study proposed a new paradigm and potential therapeutic targets to address chemotherapy sensitivity conferred by AEP-cleaved G3BP1-mediated SGs/nucleoli/mitochondria coordination.

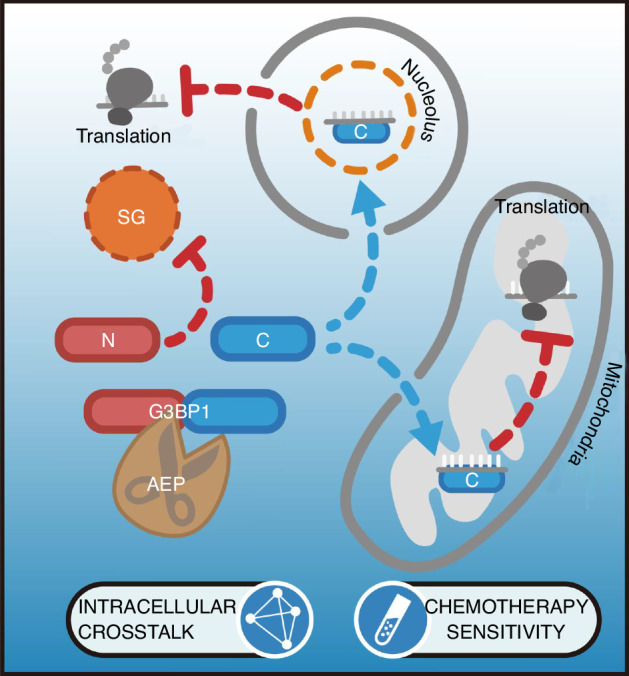

## Introduction

Osteosarcoma (OS) is the most common primary malignancy of the bone, affecting mainly children, adolescents, and young adults, and with a second peak of incidence in elderly individuals.^1,2^ Although combining surgery with chemotherapy has improved the outcomes of osteosarcoma patients, the prognosis of osteosarcomas is still unsatisfactory, as patient survival has not improved in recent decades.^3,4^ Illustrating the underlying mechanisms of tumor therapy in OS are crucial to improve the treatment of OS.

Involvement of stress granules (SGs) is important during chemotherapy.^5,6^ Besides SG, organelles, particularly nucleoli and mitochondria, are crucially involved in translation and stress response.^7–9^ Moreover, communications between nucleoli and mitochondria to balance translation have been reported for longevity.^10,11^ However, the dynamic and spatiotemporally coordinated actions of SGs with the nucleoli and mitochondria that mediate tumor cell survival under chemotherapeutic drug challenge are largely unknown.

Ras-GTPase-activating protein (GAP)-binding protein 1 (G3BP1) is a multifunctional RNA-binding protein that is essential for SG assembly. As G3BP1 is a key protein in SG formation, its regulation carries particular importance.^12^ For example, G3BP1 ubiquitination mediates SG disassembly in a context-specific manner.^13^ Translational repression of G3BP in cancer suppresses SG formation and increases stress tolerance.^14^ Caspase-8 mediated cleavage of G3BP1 subverts SG to promote viral replication.^15^ Although G3BP1 is associated with chemotherapy,^6^ its precise function and regulation remain elusive.

Asparagine endopeptidase (AEP), also known as legumain (LGMN), is a cysteine proteinase and member of the C13 family of peptidases.^16,17^ AEP is highly expressed in various solid tumors, where its high expression correlates with poor prognosis and advanced clinical stage.^18^ Our previous works revealed P53,^19^ Tmod3,^20^ and DDX3X^21^ as novel substrates of AEP in tumors. As AEP can specifically hydrolyze proteins downstream of carboxyl-terminal asparagine residues,^22^ approaches inhibiting AEP have increased feasibility and decreased toxicity and are helpful in the development of targeted therapeutics. Although AEP has been well studied in the context of neurodegenerative^23–25^ and other diseases,^26^ only a few pathological substrates for AEP have been discovered in tumors, a limitation that hinders translational studies on AEP. Therefore, the novel substrates generated after AEP cleavage and their associated functions must be systematically clarified.

In this study, chemotherapeutic drug-activated AEP specifically cleaved G3BP1 at N258/N309, producing tG3BP1-Ns, which modulated SG dynamics; moreover, tG3BP1-Cs sequestered mRNAs of ribosomal proteins in the nucleolus to repress translation in OS as well as malignant glioma. Meanwhile mitochondrial tG3BP1-Cs suppressed mtRNA translation to synergistically alleviate oxidative stresses. By utilizing in vitro assays and in vivo animal models, we demonstrated that AEP-cleaved G3BP1 modulates tumor cell survival, and that inhibition of AEP sensitizes cancer cells to chemotherapeutic drugs. High levels of AEP and G3BP1 truncations in cancerous tissues are potential factors for malignancy and poor prognosis in OS and malignant glioma.

## Results

### Chemotherapy drugs triggered AEP to specifically cleave G3BP1 at N258/N309

**Fig. 1:**
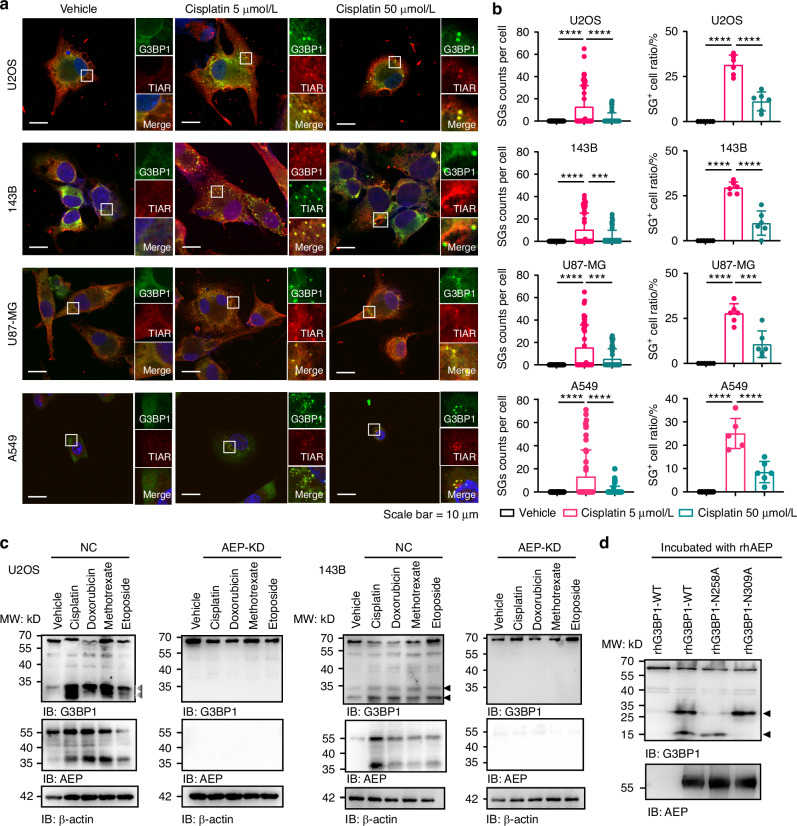
Chemotherapeutic drugs induced SG assembly and triggered AEP to specifically cleave G3BP1 at N258/N309. **a** Representative immunofluorescences (IF) images of SG assembly in U2OS, 143B, U87-MG and A549 cells exposed to cisplatin (5 and 50 μmol/L) or vehicle for 6 h. Scale Bar = 10 μm. **b** Quantification of the counts of SGs per cell (*n* = 50) and SG^+^ cell ratio (*n* = 6) in cells of (**a**). **c** WB analysis of G3BP1 and AEP in U2OS and 143B with NC or AEP-knockdown (KD) exposed to different chemotherapeutic drugs for 6 h. The arrows point out the truncated fragments of G3BP1 cleaved by AEP. **d** In vitro cleavage experiment of AEP and G3BP1 (WT and point mutants) purified recombinant proteins. Data are expressed as mean ± SD. ***P < 0.001, ****P < 0. 0001. Comparisons were conducted using one-way ANOVA

To investigate SGs in response to chemotherapy drugs, multiple drugs such as cisplatin, doxorubicin, etoposide, or methotrexate were exploited in different cancer cells using the SG markers G3BP1 and TIAR.^12,27^ Indeed, all the tested drugs induce SG assembly in OS, glioma and NSCLC cells (Figs. 1a, b, and S1). To further verify the bona fide SG induced by chemotherapeutic drugs, OS, glioma and NSCLC cancer cells were co-treated with the SG formation inhibitor ISRIB,^28^ which reverses the effects that occur downstream of eIF2α phosphorylation. As shown in Fig. S2, ISRIB suppressed SG assembly induced by chemotherapeutic drugs effectively.

However, either SG count per cell or SG^+^ cell ratio did not increase with drug concentration as expected but decreased at high drug concentrations (Figs. 1a, b, and S1). Considering the importance of G3BP1 in SG assembly, G3BP1 expression was examined in response to chemotherapeutic drugs. The Western blot results revealed dose-dependent G3BP1 cleavage fragments in cancer cells after chemotherapeutic drug treatment (Figs. 1c and S3a, S4a). Given the results of our IP-MS analysis^21^ and enzymatic substrate screening of AEP in vitro,^29^ AEP was surprisingly found to interact with various SG proteins, including G3BP1 (Fig. S3b–S3e). Moreover, AEP activity was triggered by chemotherapeutic drugs simultaneously with G3BP1 truncation, and AEP knockdown (KD) abolished G3BP1 truncation (Figs. 1c and S4a and S4b). G3BP1 cleavage was also noted after treatment with the classical SG inducer sodium arsenate or with heat shock, consistent with the observations after chemotherapeutic drug treatment (Fig. S4c and S4d).

To further verify the interaction between AEP and G3BP1, co-IP and IF experiments were performed. Pro-AEP interacts with G3BP1 in U2OS cells in response to cisplatin treatment (Fig. S4e-S4g). Given that AEP is a cysteine protease, enzyme-cut sites were further screened, and AEP was found to specifically cleave G3BP1 at N258 and N309 in in vitro enzymatic assay with purified recombinant G3BP1 and AEP protein (Fig. 1d). G3BP1/G3BP2 double-knockout (dKO) U2OS cells was further constructed, which were rescued with G3BP1-WT, G3BP1-N258A, and G3BP1-N309A to further validate that the cleavage sites at N258 and N309 of G3BP1 actually occurred in tumor cells during drug treatment **(**Fig. S4h and S4i). Through multispecies sequence alignment, asparagine N258 and N309 were found to be species conserved and precisely fit the AEP enzymatic center (Fig. S4j and S4k). Although AEP is a lysosomal compartment protease, not only our findings but those of other groups revealed that only a portion of AEP located in lysosomes **(**Fig. S4l).^19,26,30,31^

Factors that have been reported to regulate AEP activation^32–35^ were further screened. As shown in Fig. S5, only p53 KD significantly reduced pro-AEP and activated AEP in response to chemotherapeutic drug treatment. KD of CLOCK, CCAAT enhancer binding protein beta (CEBPB), or TRAF6 did not affect AEP expression. Moreover, chemotherapeutic drug treatment upregulates p53 levels consistent with AEP (Fig. S5a–c). Upon cisplatin stimulation in p53-KD OS, glioma and NSCLC cells, we observed significantly enhanced stress granule (SG) assembly. Notably, this elevated SG formation was suppressed in p53-KD/AEP rescue cells (Fig. S5d and S5e). Thus, these data suggest that chemotherapeutic drug-activated AEP can directly bind and cleave the core SG protein G3BP1 dependent on p53. Taken together, chemotherapeutic drug-triggered AEP regulates SG to specifically cleave G3BP1 at N258/N309.

### tG3BP1-Ns competitively bind to full-length G3BP1 to modulate SG dynamics

**Fig. 2:**
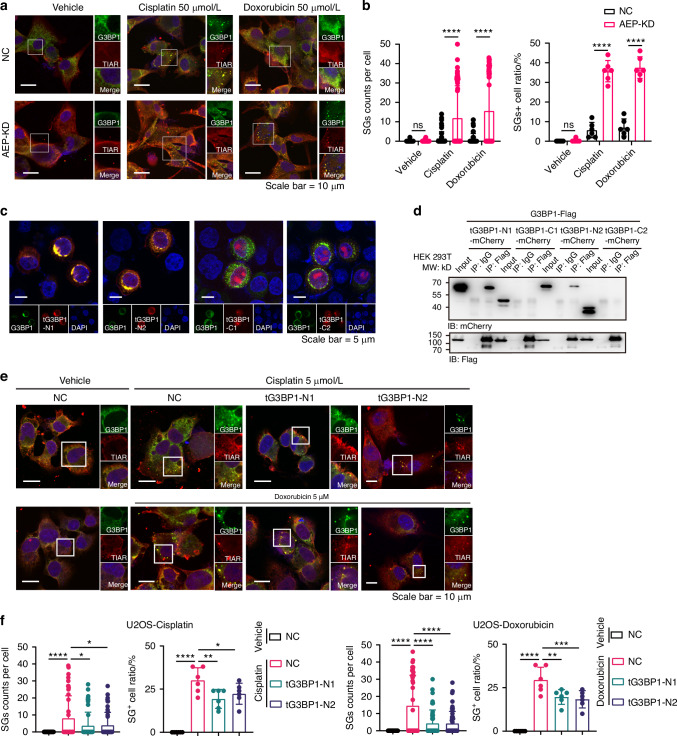
tG3BP1-Ns competitively bind to full-length G3BP1 and negatively modulate SG. **a** Representative images of SGs in U2OS cells with or without AEP-KD exposed to cisplatin (50 μmol/L), doxorubicin (50 μmol/L) for 6 h. Scale bar = 10 μm. **b** Quantification of the SG counts per cell (*n* = 50) and SG^+^ cell ratio (*n* = 6) in cells of (**a**). **c** Representative images of G3BP1-FL colocalized with tG3BP1-Ns or tG3BP1-Cs in Hela cells. Scale bar = 5 μm. **d** Co-IP and WB assays of mCherry-tagged tG3BP1-Ns or Cs cotransfected with flag-tagged full-length G3BP1 in HEK293T. **e** Representative images of SGs in tG3BP1-Ns overexpressed U2OS cells exposed to cisplatin (5 μmol/L), doxorubicin (5 μmol/L) for 6 h. Scale bar = 10 μm. **f** Quantification of SG counts per cell (*n* = 50) and SG^+^ cell ratio (*n* = 6) of (**e**). Data are expressed as mean ± SD. *P < 0.05, **P < 0.01, ***P < 0.001, ****P < 0.000 1. ns no significance. One-way ANOVA

To elucidate the functions of cleaved G3BP1 in chemotherapeutic drug-induced SG, whether AEP influences SG dynamics was initially investigated. AEP KD significantly increased the SG formation compared with NC U2OS cells after cisplatin or doxorubicin treatment (Fig. 2a, b). The domains of G3BP1 were analyzed, and tG3BP1-Ns was found to have a dimerization domain (NTF2L) that might complex with full-length G3BP1 (Fig. S6a).^36,37^ Both the IF and co-IP experiments verified the specific interaction between tG3BP1-Ns and G3BP1 and showed that tG3BP1-Cs cannot complex with G3BP1 (Fig. 2c, d). Moreover, tG3BP1-Ns overexpression (mCherry tagged) decreased the SG assembly significantly (Fig. 2e, f).

As chemotherapeutic drug-induced SGs are associated with tumor cell survival, the effects of AEP and associated G3BP1 truncations were analyzed. The apoptosis assay revealed that AEP KD significantly reduced tumor cell survival, whereas rescue with tG3BP1s increased cancer cell survival (Fig. S6b). Thus, these results indicate that tG3BP1-Ns produced by AEP-mediated cleavage of G3BP1 competitively bind to full-length G3BP1 to modulate SG dynamics and partially promote cancer cell survival in response to chemotherapeutic drugs.

### tG3BP1-Cs sequestered mRNAs of ribosomal proteins in the nucleolus to repress translation after chemotherapeutic drug treatment

**Fig. 3:**
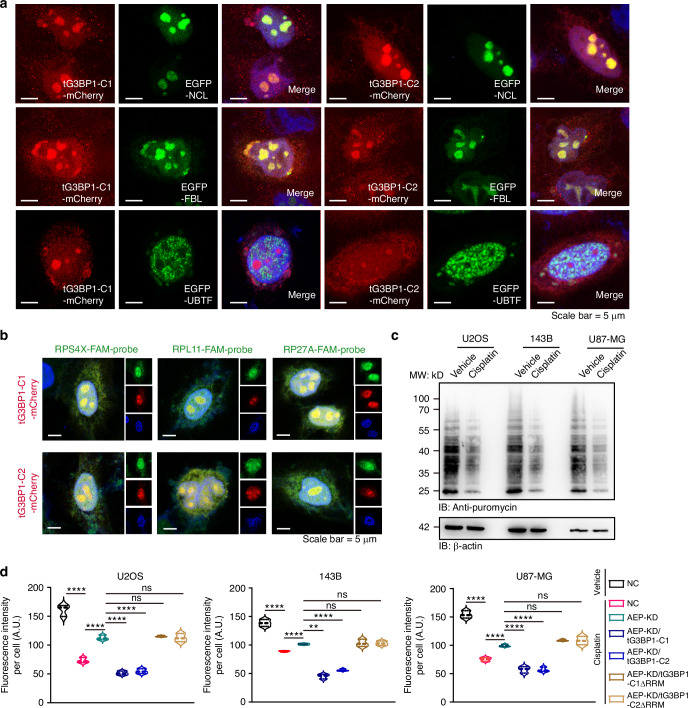
tG3BP1-Cs translocate into the nucleolus and sequester mRNAs of ribosomal proteins in the nucleolus to inhibit cellular translation. **a** Representative images of the sub-nucleolar localization of tG3BP1-Cs and sub-nucleolar markers in Hela cells. Scale bar = 5 μm. **b** Representative images of FISH and IF assays present the nucleolar colocalization of tG3BP1-Cs with FAM-conjugated probes of ribosomal mRNAs, RPS4X, RPL11, and RP27A. **c** SUnSET experiments analyzed the protein synthesis in U2OS, 143B, and U87-MG cells treated with cisplatin (50 μmol/L) or vehicle for 6 h. **d** Quantification of protein synthesis of the aforementioned cell lines exposed to cisplatin (50 μmol/L) or vehicle for 6 h were detected with the Click-iT HPG system (*n* = 3). Data are expressed as mean ± SD. **P < 0.01, ****P < 0.000 1. ns no significance. One-way ANOVA

Beyond the effects of tG3BP1-Ns, tG3BP1-Cs functions were also investigated. Unlike G3BP1 or tG3BP1-Ns, tG3BP1-Cs had substantial nucleolar localization (Fig. S6c). Further studies have revealed that tG3BP1-Cs were located at the granular center (GC) and dense fibrillar center (DFC), not at the fibrillar center (Figs. 3a, S6d, and S6e). The GC and DFC are major sites of ribosome production. MS analysis of the immunoprecipitated tG3BP1-Cs complex showed that the tG3BP1-Cs complex contained many ribosomal proteins (Fig. S6f and S6g, Table S1), further verifying the GC and DFC localization of tG3BP1-Cs. Surprisingly, mitochondrial proteins were also detected in the tG3BP1-Cs complex (Fig. S6f and S6g). The RNA recognition motif (RRM) is the major domain in tG3BP1-Cs (Fig. S7a). The RRM domain was deleted, or nuclear localization sequence (NLS) was predicted, only RRM-deleted tG3BP1-Cs lost nucleolar localization, and NLS deletion still resulted in nucleolar localization (Fig. S7b).

Considering the RNA-binding ability of the RRM, a PAR-CLIP assay revealed that tG3BP1-Cs can bind to RNAs, whereas tG3BP1-Ns exhibit only weak binding to RNAs (Fig. S7c). PAR-CLIP-seq revealed that tG3BP1-Cs bind mRNAs of ribosomal proteins associated with translation (Fig. S7d and S7e, Table S2). FISH/IF experiments confirmed that RPS4X, PRL11, and RPL27A mRNA complexes with tG3BP1-Cs in the nucleus (Figs. 3b and S7f).

As ribosomal proteins are crucial for translation, we sought to determine whether the translation ability is inhibited by tG3BP1-Cs during chemotherapeutic drug treatment. The puromycin incorporation assay (SUnSET) revealed the translation level of cancer cell lines was significantly repressed after cisplatin treatment (Fig. 3c). Newly synthesized proteins were further labeled with the noncanonical methionine analog homopropargylglycine (HPG), which was visualized with azide-containing fluorescent dyes through a copper-catalyzed cycloaddition (CLICK) reaction. The HPG labeling assay results were consistent with SUnSET (Figs. 3d and S8a), indicating that tG3BP1-Cs production (259-466, 310-466) might be one of the important mechanisms responsible for translation inhibition in response to chemotherapeutic drug treatment. These results indicate that tG3BP1-Cs sequester mRNAs of ribosomal proteins in the nucleolus to repress translation upon chemotherapeutic drug treatment.

### tG3BP1-Cs bind to mitochondrial mRNA targets and suppress their translation to alleviate mitochondrial stress

**Fig. 4:**
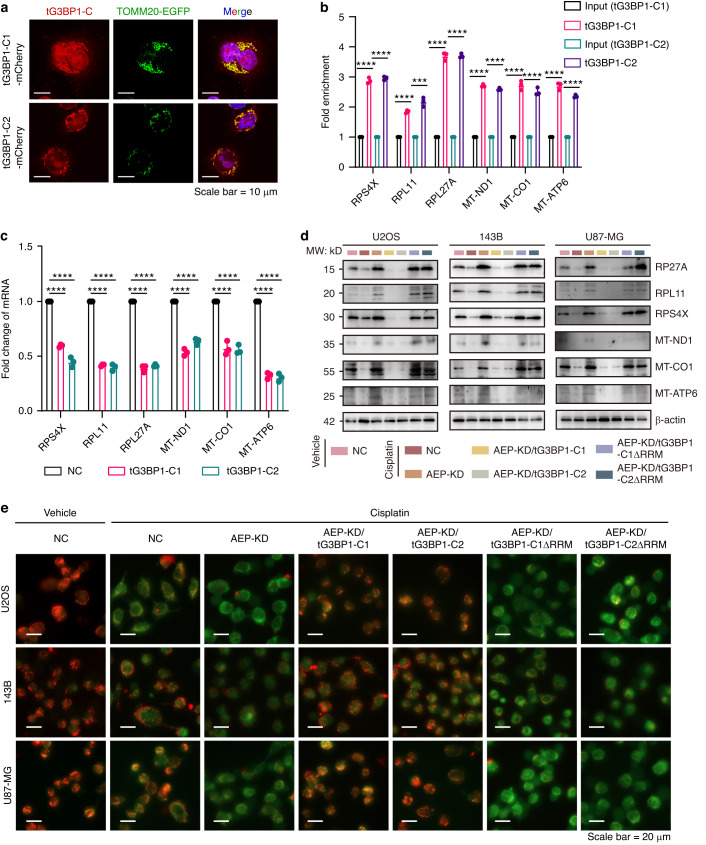
tG3BP1-Cs bind to mitochondrial mRNA targets and suppress their translation to alleviate mitochondrial stress. **a** Representative images of the colocalization of tG3BP1-Cs with the mitochondrial marker TOMM20 in Hela cells. Scale bar = 10 μm. **b** RNP-IP analysis of the mRNA target encoding ribosomal proteins and oxidative phosphorylation binding to tG3BP1-Cs (*n* = 3) in tG3BP1-Cs overexpressed U2OS cells. **c** Ribosome profiling-qPCR analysis demonstrated that tG3BP1 overexpression in U2OS cells significantly downregulates mitochondrial genes translation. **d** WB analysis of mitochondrial genes expression in cell lines exposed to cisplatin (50 μmol/L) or vehicle for 6 h. **e** Cisplatin-induced mitochondrial damage was detected by JC-1 probe staining in cells of (**d**). Data are expressed as mean ± SD. ***P < 0.001, ****P < 0.000 1. One-way ANOVA

As shown in Fig. S6e, tG3BP1-Cs are also complex with mitochondrial oxidative phosphorylation proteins. We sought to determine whether tG3BP1-Cs could influence mitochondrial functions. In the examination of a mitochondrial marker (TOMM20-EGFP), tG3BP1-Cs showed significant localization in the mitochondria (Figs. 4a and S8b). The PAR-CLIP results indicated that tG3BP1-Cs can bind to oxidative phosphorylation associating MT-mRNAs (Fig. S8c). PAR-CLIP data were confirmed using RNP-IP for cellular mRNA target encoding ribosomal proteins and oxidative phosphorylation (Fig. 4b). The consensus RNA motif that tG3BP1-Cs recognized was further analyzed, and PAR-CLIP-seq derived consensus motif analysis revealed three potential consensus RNA motifs, namely, (CCU[GCU][CG]CU[CG], [UG]GGCCA^38^ [GC], and G[CG][UC]G[CG]AG). RNA pulldown assay verified that CCU[GCU][CG]CU[CG] motif is preferentially bound to tG3BP1-Cs (Fig. S8d and S8e).

Polysome profiling revealed a significant reduction in polysome-associated ribosomal protein-encoding mRNAs after tG3BP1-C overexpression (Fig. 4c), suggesting impaired translational engagement. WB further showed that the protein levels of these genes were increased in AEP-KD cells and that rescue with tG3BP1-Cs in AEP-KD cells significantly reduced the protein levels, whereas rescue with RRM-deleted tG3BP1-Cs did not (Fig. 4d). An OCR assay was done in WT, AEP-KD, AEP-KD tG3BP1-Cs rescue, and AEP-KD tG3BP1-Cs-delRRM rescue cells after cisplatin treatment to determine whether mitochondrial activity was disturbed by these changes. The results revealed that the OCR significantly increased upon AEP-KD and decreased in cells rescued with WT tG3BP1-Cs (Fig. S9a).

As the generation of ROS is accompanied by mitochondrial OXPHOS, the levels of ROS and antioxidant GSH were measured. The results showed that compared with those in NC cells, ROS levels were reduced in AEP-KD cells and that rescue with tG3BP1-Cs in AEP-KD cells significantly increased ROS levels, whereas rescue with RRM-deleted tG3BP1-Cs did not (Fig. S9b and S9c). The change in the GSH level followed the opposite pattern (Fig. S10a). Can mitochondrial damage induced by chemotherapeutic drugs be alleviated by AEP/tG3BP1-Cs via reductions in OXPHOS and accompanying ROS production? Mitochondrial function was further analyzed after drug treatment by JC-1 staining. The results showed that the formation of JC-1 aggregates in the mitochondria was reduced in AEP-KD cells compared with NC cells and that rescue with tG3BP1-Cs in AEP-KD cells significantly increased the formation of JC-1 aggregates in the mitochondria, whereas rescue with RRM-deleted tG3BP1-Cs did not (Figs. 4e and S10b). According to TCGA data mining, high expression levels of MT-CO1, and MT-ATP6, MT-ND1 predict a better prognosis (Fig. S10c), indicating that reduced expression of these genes might be associated with a poor therapeutic response.

Mitochondria are involved in various cell death pathways. Cancer cells were then treated with mitochondria-associated cell death inhibitors that might influence chemosensitivity and found that the cuproptosis inhibitors TTM and Rotenone^39^ significantly increased cancer cell survival after drug treatment compared with the apoptosis inhibitor Z-VAD-FMK and ferroptosis inhibitor Fer-1 (Fig. S11a and S11b). Consistently, the Cu content changed with AEP/tG3BP1-Cs introduction (Fig. S11c), an effect that might confer chemotherapy response. Low expression of *LIAS*, *PDHA1*, and *PDHB*, which suppress cuproptosis, predicted poor survival (Fig. S11d).

These results indicate that tG3BP1-Cs produced by AEP-mediated cleavage of G3BP1 binds to mitochondrial mRNA targets and suppresses translation to alleviate mitochondrial stress induced by chemotherapeutic drugs.

### AEP-mediated cleavage of G3BP1 promotes chemotherapeutic drug sensitivity in multiple cancer models

**Fig. 5:**
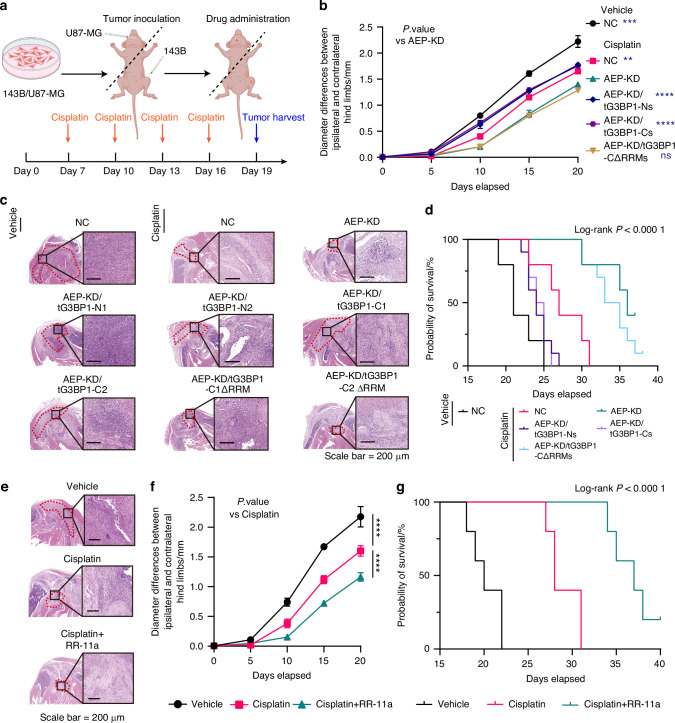
AEP-mediated cleavage of G3BP1 promotes drug resistance in animal models. **a** Diagram of the xenotransplantation of 143B or U87-MG cell lines into mice and the drug treatment schedule. **b** Line chart of the diameter between the ipsilateral and contralateral hind limbs of mice in the xenograft OS model in groups as indicated (*n* = 5). Two-way ANOVA. **c** Representative H&E images of tumors in mice of (**b**). The red dashed line delineates the tumor region. Scale bar = 200 µm. **d** Kaplan‒Meier survival curves for the OS-bearing xenografted mice of (**b**) (*n* = 5). log-rank (Mantel–Cox) test, *P* < 0.000 1. **e** Representative H&E images of tumors in the xenograft OS model of mice treated with cisplatin or RR-11a. Scale bar = 200 µm. **f** Line chart of the diameter between the ipsilateral and contralateral hind limbs of mice in the xenograft model in (**e**) (*n* = 5). Two-way ANOVA. **g** Kaplan‒Meier survival curves for the OS-bearing xenografted mice in (**f**) (*n* = 5). log-rank (Mantel-Cox) test, *P* < 0.000 1. Data are mean ± SD. **P < 0.01, ***P < 0.001, ****P < 0.000 1. ns no significance

Considering the importance of these molecular events, the pathological functions of AEP/G3BP1 truncations were investigated in chemotherapeutic drug sensitivity. Orthotopic OS and glioma animal models were utilized to investigate the importance of AEP/G3BP truncations (Fig. 5a). The results in the OS animal model showed that AEP KD significantly reduced tumor growth after cisplatin treatment (4 mg/kg BW, twice a week, i.p.). Rescue with tG3BP1-Cs or tG3BP1-Ns significantly increased tumor growth, whereas rescue with RRM-deleted tG3BP1-Cs did not (Figs. 5b, c, and S12a). The survival of OS-bearing mice was analyzed, and AEP KD prolonged survival, whereas rescue with tG3BP1-Cs or tG3BP1-Ns shortened the survival time. Rescue with RRM-deleted tG3BP1-Cs did not shorten the survival time (Fig. 5d). The orthotopic glioma animal models exhibited similar results (Fig. S12b, S12c, and S13a, S13b).

Moreover, cotreatment with an AEP inhibitor (RR-11a) and cisplatin suppressed tumor growth in OS animal models (Figs. 5e, f and S13c). The survival of glioma tumor-bearing mice was significantly prolonged when cisplatin was combined with RR-11a (20 mg/kg BW daily, i.p.) (Fig. 5g). The glioma animal models exhibited similar results (Fig. S13d-S13g). Thus, these results indicate that AEP-mediated cleavage of G3BP1 promotes chemotherapeutic drug sensitivity.

### High levels of AEP and truncated G3BP1 were found in OS and glioma tumor tissues and were strongly associated with poor prognosis

**Fig. 6:**
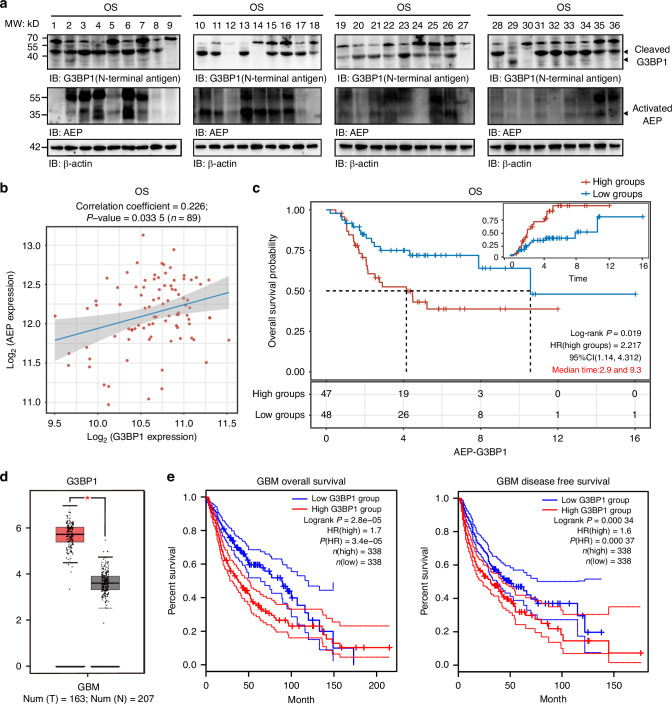
High levels of AEP and G3BP1 truncations in OS tumor tissues and strong association with poor prognosis. **a** WB analysis of G3BP1 cleavage and AEP activation in OS tumor tissues. **b** Correlation analysis of G3BP1 and AEP expression from TARGET RNA-seq data (*n* = 89). **c** Kaplan‒Meier survival curves for patients with low and high expression of G3BP1 and AEP [*n*(Low) = 48; *n*(High) = 47]. **d** Comparative expression analysis of G3BP1 in normal tissues versus glioblastoma (GBM) tumor tissues from TCGA RNA-seq database. **e** Kaplan‒Meier survival curves for GBM patients with low and high expression of G3BP1 [*n*(Low) = 338; *n*(High) = 338]. Data are expressed as mean ± SD. *P < 0.05. log-rank (Mantel-Cox) test

Relative to those in normal- or low-grade glioma tissues, high levels of AEP and G3BP1 truncations were found in high glioma tissues and OS tumor tissues (Figs. 6a and S14a, S14b). Are AEP and G3BP1 associated with cancer malignancy and prognosis? TCGA data analysis revealed that G3BP1 and AEP are highly expressed in tumor tissues and is significantly associated with poor prognosis in glioma and osteosarcoma (Fig. 6c–e). Further IHC staining of a glioma tissue microarray revealed that a high proportion of nuclear G3BP1 was associated with poor prognosis (Fig. S14c and S14d). Positive associations among AEP, G3BP1, and nuclear G3BP1 were found (Figs. 6b, S14e-S14f, Tables S3, S4). Thus, high levels of AEP and G3BP1 truncations in OS and glioma tumor tissues are strongly associated with tumor malignancy and poor patient prognosis.

Overall, a new paradigm for chemosensitivity conferred by AEP-cleaved G3BP1-mediated crosstalk was found between SGs and nucleoli/mitochondria.

## Discussion

Studies have shown that chemotherapeutic agents, including cisplatin, etoposide, oxaliplatin, paclitaxel, and doxorubicin, induce SG formation. However, the dynamics of SGs and their underlying molecular mechanisms in response to chemotherapeutic drugs remain elusive. In this study, chemotherapeutic drug triggered protease AEP to cleave G3BP1-producing truncates, which are crucial communicators among SG/nucleoli/mitochondria, which provide profound insight into the organelle crosstalk under stress conditions. Targeting AEP decreased cancer cell survival in GBO and animal models. These findings indicate that the AEP-mediated cleavage of G3BP1 could have prognostic/therapeutic potential in OS as well as malignant glioma.

This study reveals that G3BP1s truncations produced by AEP cleavage have different subcellular localizations and pathological functions, particularly tG3BP1-C. In fact, the generation of proteoforms through proteolytic cleavage is a widespread intracellular posttranscriptional modification modality with diverse important physiological and pathological functions.^40^ The cleavage of amyloid beta precursor protein^41^ by AEP results in the production of truncated APP (C586-689), which binds to CEBPB, leading to its nuclear translocation and promoting the progression of Alzheimer’s disease.^34^ Likewise, Tmod3-C translocates to the nucleus and affects transcription.^20^ Our recent work revealed that AEP-cleaved DDX3X induces alternative RNA splicing events to mediate cancer cell adaptation in harsh microenvironments,^21^ indicating that AEP has important functions in tumor stress response via distinct substrates. Further studies are needed to determine whether AEP can also affect other SG proteins, particularly G3BP2.

Strikingly, AEP-cleaved tG3BP1-Cs sequestered mRNAs of ribosomal proteins in the nucleoli, repressing translation. Studies have uncovered nucleoli as a stress sensor and signaling hub when cells encounter various stress conditions, such as nutrient deprivation, DNA damage, and oxidative and thermal stress.^9^ Nucleolar accumulation of mRNAs such as c-myc has been observed in tumor cells.^42^ tG3BP1-C-mediated nucleolar accumulation of mRNAs of ribosomal proteins may be a new strategy for tumor cells to manage stresses by delivering stress signals from SGs to the nucleus. Although our data indicates that AEP can affect protein synthesis, the participation of new ribosomal proteins in this process remains unclear. Moreover, full-length G3BP1 did not reduce after AEP cleaved as expected, indicating that G3BP1 expressing might be increased after chemotherapeutic drug treatment.

Moreover, AEP-cleaved tG3BP1-Cs also suppressed the translation of mitochondrial mRNAs. The mitochondrial stress response is a key mechanism for cancer cell survival, leading to cancer progression and therapy.^43^ Despite the reports of the crosstalk between SGs and paraspeckles,^44^ the communication between SGs and other organelles is poorly understood. Intracellular protein trafficking among SG, nuclei, nucleoli, cytosol, and mitochondria has been found.^45^ AEP-cleaved G3BP1 modulates the crosstalk between the SGs and nucleoli/mitochondria, orchestrating chemotherapeutic drug sensitivity by suppressing translation and oxidative stresses, which is a new paradigm. However, how tG3BP1-C shuttles between the nucleoli and mitochondria and why these target mRNAs are recruited by tG3BP1-C remain unknown. The exact function and mechanism of tG3BP1-C binding to these mRNAs remain elusive.

Notably, a regulated pattern was found in which the AEP cleavage site was primarily located at the IDR, a pattern of cleavage that can preserve the intact functional domains of substrates, indicating the important role of AEP in precision proteolytic events. A study reported the narrow cleavage specificity combined with clear cleavage preference for unstructured secondary regions in substrates.^46^ However, the precise regulatory mechanisms of the IDR remain largely unknown. Further investigations are warranted to show the modifications and regulators of the IDRs of these substrates under different tumor-related stresses.

In summary, our findings reveal a previously unappreciated role of AEP and G3BP1 as modulators of the communication between SGs and nucleoli/mitochondria, provide profound insight into organelle crosstalk under stress conditions, and highlight the therapeutic potential of targeting AEP or SGs to reinforce the efficacy of chemotherapy for refractory tumors, especially OS and malignant glioma. Regrettably, the survival data of these patients could not be retrieved due to logistical constraints. In the future, we will place greater emphasis on the acquisition and collection of clinical data to ensure more reliable experimental outcomes.

## Materials and methods

### Chemotherapeutic drug treatment in vitro assay

The wild-type (WT) cell lines of U2OS, 143B, U87-MG and A549 were seeded in 12-well plates with or without round coverslips. The next day, cisplatin, doxorubicin, etoposide, and methotrexate in appropriate concentration were added in culture as needed or proper vehicle solution for 6 h. The cells were treated appropriately for WB, fluorescence-activated cell sorting, and IF.

### Immunofluorescence staining (IF)

In this study, the indicated cells were plated on round coverslips in 6-well plates, treated with drugs, and fixed with 4% poly formaldehyde at room temperature (RT). The cell coverslips were washed three times with 1x TBST buffer, with each washing 5 min. The coverslips were incubated at RT with approximately 100 μL of blocking and permeabilization buffer for 1 h. The blocking and permeabilization buffer were removed, and the primary antibodies anti-G3BP1 and anti-TIAR (both are markers of SGs) diluted in an antibody dilution buffer (~100 μL, dilution: 1:50) were added to the coverslips and incubated overnight at 4 °C. On the following day, the primary antibody solution was removed, and the tissue slides were quickly washed three times using 1x TBST buffer in the staining dish, with each wash lasting 5 min. The coverslips were incubated at RT with approximately 100 μL of secondary antibodies (1:500 dilution) and DAPI (1:10 000 dilution), both diluted in an antibody dilution buffer, for 1.5 h. The secondary antibody/DAPI solution was removed, and the coverslips were quickly washed three times using 1x TBST buffer in the staining dish, with each wash lasting 5 min. For mounting, the slides were applied approximately 50–100 μL of mounting medium to each slide and covered with a square coverslip. The slides were allowed to dry overnight in a dark environment. The next day, the coverslip was sealed to the edge of the slide using transparent nail polish, which was left to dry for 1 h. Finally, the slides were imaged under a confocal microscope.

### SG quantitative analysis

The chemotherapy drug-induced SG were visualized via IF with anti-G3BP1 and anti-TIAR antibodies. SGs were quantified with the SG counts per cell and the SG-positive cell count ratio.

### WB

The lysates of the indicated cells or tissue were prepared in RIPA buffer (Cat# R0020, Solarbio, Beijing, China) containing phenylmethylsulphonyl fluoride (Cat# 329-98-6, Sigma-Aldrich) and Complete Protease Inhibitor Cocktail (Cat# 4693116001, Roche, Basel, Switzerland). Lysates were incubated for 30 min on ice and centrifuged for 10 min at 12 500 × *g* and 4 °C. A BCA protein assay kit (Cat# 23225, Thermo Fisher Scientific) was used to determine the protein concentrations. Sample proteins were separated by sodium dodecyl-sulfate polyacrylamide gel electrophoresis and transferred to PVDF membranes. After blocking with TBST (TBS with 0.1% Tween 20) containing 5% skim milk, the membranes were incubated with primary antibodies overnight at 4 °C. The membranes were then incubated with appropriate HRP-conjugated secondary antibodies at RT for 1 h. A ChemiDoc Imaging System (Cat# 12003154, ChemiDoc™ MP Imaging System, Bio-Rad, Hercules, CA, USA) was used to detect chemiluminescence.

### Co-immunoprecipitation (IP) and mass spectrometry (MS) analysis

AEP-KO U2OS infected with human AEP-expressing lentivirus were treated with 50 μmol/L cisplatin or vehicle for 6 h. Cells were lysed in IP buffer (Cat# 87787, Thermo Fisher Scientific) and then subjected to IP using an anti-AEP antibody (1:50 dilution). A portion of the immunoprecipitate was used for silver staining, and the remaining immunoprecipitate was sent to Oebiotech Co., Ltd. (Shanghai, China), for LC-MS/MS analysis.

MS analysis of AEP-KD/tG3BP1-C rescue U87-MG cells were performed in a similar process. Cell lysates were subjected to IP using an anti-Flag antibody (1:100 dilution).

### AEP cleaved G3BP1 in vitro

Recombinant human G3BP1 (10 μg) (WT, N258A, and N309A point mutants) was incubated with recombinant human AEP or in 200-µL reaction buffer (20 mmol/L citric acid, 60 mmol/L Na_2_HPO_4_, 1 mmol/L EDTA, 0.1% CHAPS and 1 mmol/L DTT, pH 6.0) for 30 min at 37 °C and analyzed by WB.

### Cell apoptosis detection

The cell lines were treated with 50 μmol/L cisplatin for 12 h; (1–5) ×10^5^ cells were collected and treated with BD Pharmingen™ FITC Annexin V Apoptosis Detection Kit (Cat#556547, BD Biosciences, New Jersey, USA) according to the manufacturer’s protocol. Samples were analyzed on a BD LSR Fortessa X-20 flow cytometer (BD Biosciences).

### PAR-CLIP sequencing and data analysis

Stable U2OS cells transfected with pHY023-tG3BP1-C and pLKO.1-shAEP were cultured in 15 cm^2^ dishes. When the cell confluence reached 70%, 50 μmol/L cisplatin and 100 μmol/L 4-SU were added to the culture, and the cells were incubated for 12 h. The medium was aspirated, and cells were added with ice-cold phosphate-buffered saline (PBS). The lids were removed, and the cells were irradiated twice (150 mJ/cm^2^) in a UVP Crosslinker with a 365-nm lamp. The cells were harvested and lysed with an IP buffer (1 mmol/L DTT, 1 × complete protease inhibitor, RNase inhibitor added) for 20 min. The samples were centrifuged at 4 °C and 22 000 × *g* for 10 min, and the supernatant was collected carefully. RNase T1 (1 U/μL) was added to the lysate, which was incubated at 25 °C for 15 min. The lysate was added with prewashed protein A/G agarose beads (10 μL/100 µL) and an anti-Flag-tag antibody (1:100) and incubated overnight on a rocking shaker. On the following day, the beads were washed three times with a lysis buffer, and RNAs were extracted using TRIzol. RNA-sequencing was performed by Novogene.

To generate the count matrices, raw FASTQ files of PAR-CLIP sequencing data were processed using STAR, whereas the GRCh38/hg38 index genome was used for the alignment of the PAR-CLIP-seq data. PAR-CLIP-seq analysis was conducted using R (version 3.6.0) package Limma (version 3.56.0) to process the enrichment matrices. Gene Expression Omnibus and Kyoto Encyclopedia of Genes and Genomes enrichment analyses were performed with the online tool DAVID (https://david.ncifcrf.gov/tools.jsp), and the plots of these results were produced online by SangerBox (http://sangerbox.com/). The sequencing coverage histogram was generated with IVG (version 2.15.2).

### DNA probes and reagents

All probes used in FISH were biotin-tagged and synthesized by Sangon Biotech Co., Ltd., and detail sequences are listed in Table S5. SA -onjugated Cy3 and FITC (Cat#D110512, Sangon Biotech Co., Ltd.) were used for FISH. Other reagents and buffers used in FISH were as follows: 20×SSC buffer, DEPC Treated (Cat# B548110, Sangon), Formamide deionized (Cat#A600211, Sangon).

### FISH

U2OS cells were seeded in 12-well cell culture plates with round coverslips and treated with cisplatin (50 μmol/L) for 6 h. The cells were fixed with 4% formaldehyde at RT for 15 min. The FISH kit was purchased from GenePharma, and subsequent FISH assays were performed according to manufacturer’s protocol.

### SUnSET assay

To monitor the protein synthesis of U2OS, 143B, and U87-MG stable cell lines after treatment with cisplatin, SUnSET assays were performed. These cell lines were seeded in 10-cm dishes and treated with 50 μmol/L cisplatin for 6 h until the confluence reach 80%. Puromycin was added with final concentration at 10 µg/mL and cultured for 1 h. The cells were collected, WB was performed, and anti-puromycin antibody was used for detection.

### Polysome profiling with RNA-qPCR

To assess translational activity of target genes’ mRNA, cancer cell lines are treated with cycloheximide (CHX, 100 μg/mL, 5 min) to arrest ribosomes on mRNAs, followed by rapid harvesting and washing in ice-cold PBS containing CHX. Cells are lysed in a buffer containing 20 mmol/L Tris (pH 7.5), 150 mmol/L KCl, 5 mmol/L MgCl_2_, 1% Triton X-100, 1 mmol/L DTT, CHX, and RNase inhibitors to preserve ribosome-mRNA complexes. After centrifugation to remove debris, the lysate is layered onto a pre-chilled 10%–50% linear sucrose gradient (prepared in 20 mmol/L Tris, 150 mmol/L KCl, 5 mmol/L MgCl_2_) and subjected to ultracentrifugation (35 000 r/min, 4 °C, 2.5 h, SW41 rotor) to separate ribosomal fractions. Gradients are fractionated using a density gradient system with continuous UV monitoring, collecting 12–14 fractions corresponding to monosomes (80S) and polysomes. RNA is extracted from each fraction using TRIzol, treated with DNase I, and reverse-transcribed into cDNA. Target mRNA levels in polysome versus monosome fractions are quantified by qPCR with gene-specific primers, normalized to housekeeping genes total RNA input. Higher mRNA enrichment in polysomal fractions indicates active translation.

### Protein synthesis assay with the Click-iT homopropargylglycine (HPG) system

U2OS, 143B, and U87-MG cells were synchronized using 200 nmol/L dexamethasone for 2 h. The medium was then replaced with dexamethasone-free DMEM. To prepare the Click-iT HPG working solution, a stock solution was diluted 1:1 000 in a prewarmed L-methionine-free medium to a final HPG concentration of 50 μmol/L. Thirty minutes before the designated time, the culture medium was replaced with an HPG working solution. After 30 min of incubation at 37 °C, the cells were washed once with PBS and fixed with 4% formaldehyde. Then, Alexa Fluor 488 azide was conjugated to HPG through a click reaction according to the manufacturer’s protocol. HPG-incorporated proteins were subsequently detected by fluorescence microscopy.

### RNA IP (RIP)-qPCR assay

The RIP assay was conducted to validate the mRNA binding of RPS4X and MT-ND1 to tG3BP1-C. U2OS overexpressing flag-tagged tG3BP1-C1 and tG3BP1-C2 or control cells were cultured in 10-cm dish and treated with cisplatin (50 μmol/L) for 6 h. The cells were fixed with 4% formaldehyde at RT for 15 min, and the fixation was then stopped with 0.125 mol/L glycine (in PBS) for 5 min. The collected cells were washed twice with 1 mL of ice-cold PBS, spinned, and the supernatant was discarded. Moreover, 500 µL of the IP lysis buffer (protease inhibitor and RNase inhibitor included) was added and incubated on ice for 15 min. The lysate was centrifuged at 15 000 × *g* for 15 min at 4 °C, 50 µL (10% v/v) of the supernatant was taken and restored at 20 °C as input, 20 µL of the anti-flag antibody and isotype mouse IgG were added, respectively, and 50 µL Protein A/G Plus agarose beads were mixed to the rest lysate. The mixture was incubated on a rotating shaker at 4 °C overnight. The next day, the mixture was spinned at 4 °C, the supernatant was discarded, and beads were washed four times with ice-cold PBS. RNA was isolated from the input lysate and beads with RNAclean Kit (Cat#DP412, Tiangen Biotech) according to the manufacturer’s protocol. To quantify the mRNA level of RPS4X and MT-ND1, in input, IP, and IgG groups, RT-qPCR was performed. The specific primers for the above mRNA are listed in Table S5. The final data were analyzed according to the following formulas:

$$\begin{array}{c}\varDelta C{t}_{{normalized\; RIP}}=\left[{AverageC}{t}_{{RIP}}-{AverageC}{t}_{{Input}}-{lo}{g}_{2}^{{Input\; dilution\; factor}}\right]\\ \varDelta C{t}_{{normalized\; RIP}/{negative\; control}}=\varDelta C{t}_{{normalized\; RIP}}-\varDelta C{t}_{{negative\; control}}\\ {Fold\; Enrichment}={2}^{-{\varDelta {Ct}}_{{normalized\; RIP}/{negative\; control}}}\end{array}$$

### Ribosome profiling

To measure the translation of the target mRNA in NC and tG3BP1-Cs-overexpressed U2OS cells, ribosome profiling assay was performed. The steps for ribosome profiling were slightly modified based on the literature (Ingolia et al., 2012). RNAs in ribosome pellets extracted from the aforementioned procedure were purified with RNAclean Kit (Cat#DP412, Tiangen Biotech). The sequences of RT-qPCR primers are listed in Table S5.

### RNA-motif pulldown assay

In the PAR-CLIP dataset, 10 candidate genes were identified, and the MEME suite was utilized (Bailey et al., 2015) for consensus motif prediction, ultimately selecting three candidate motifs. Subsequently, the motif sequences of two candidate genes were chosen, motifs were synthesized with approximately 30 nucleotides flanking RNAs and their respective antisense oligonucleotides, and 5’ biotin-modified RNAs were synthesized.

Separately, tG3BP1-C1/C2 was overexpressed in HEK 293 T cells in a 15-cm dish, and cell lysis was induced using RIPA cell lysis buffer to obtain the cell lysate including tG3BP1-Cs for the subsequent pulldown assay. Initially, the biotin-modified RNA was preincubated with streptavidin-magnet beads at RT for 1 h. Subsequently, the above cell lysate was added to each preincubated mixture and incubated overnight at 4 °C on a rocking shaker. The following day, the samples were washed four times with ice-cold PBS, and pulldown proteins were eluted using a 5× SDS sample loading buffer. Pulldown results were assessed by WB.

### Detection of the mitochondrial membrane potential

The mitochondrial membrane potential was applied to estimate mitochondrial damage in stable cell lines. The stable cells were seeded in 6-well cell culture plates and treated with cisplatin (50 μmol/L) and appropriate vehicle solution for 6 h. The mitochondrial membrane potential assay kit with JC-1 (Cat# C2006, Beyotime) was used for the detection of mitochondrial membrane potential according to the manufacturer’s protocol.

### Oxygen consumption rate (OCR) assay

Stable cell lines of U2OS, 143B, and U87-MG were seeded in 96-well cell culture plates and treated with cisplatin (50 μmol/L) or appropriate vehicle solution for 6 h. OCR Fluorometric Assay Kit (Cat# E-BC-F068, Elabscience, Wuhan, China) was used according to manufacturer’s protocol. In this experiment, JC-1 staining was used to assess mitochondrial membrane potential. The green signal represents JC-1 monomers (indicative of depolarized mitochondria, damaged mitochondria), while the red signal corresponds to JC-1 aggregates (indicative of polarized mitochondria, healthy damaged mitochondria).

### Patient specimens

This study included 95 glioma and 36 OS specimens that were clinically and histopathologically diagnosed at Renji Hospital. Their diagnoses were independently rereviewed by two pathologists and classified by the World Health Organization criteria. Freshly frozen glioma tissues [low-grade glioma (LGG), *n* = 10; high-grade glioma (HGG), *n* = 72; recurrent glioma, *n* = 18] and five normal brain tissues were procured from the Neurosurgery Department of Renji Hospital. All the tissues were made into tissue chips.

### In vivo xenograft model

Athymic male nu/nu mice (Lingchang Biotech, Shanghai, China) were used as glioma models. U87-MG cells (5 × 10^5^/5 μL) were stereotactically injected into the right ganglia region. Chemotherapy treatment (cisplatin, 4 mg/kg BW) was initiated 7 days post tumor implantation to allow establishment of measurable lesions. Therapy was administered via intraperitoneal injection every 3 days for a total of 4 cycles. Mice were monitored daily and examined by magnetic resonance imaging (MRI) when they exhibited weight loss or neurologic impairment. Tumor diameters were measured from the MR images. The mice were euthanized when they were severely emaciated and depressed.

For the OS model, athymic female nu/nu mice aged 6 weeks were used. 143B cells (5 × 10^5^/10 µL) were inoculated into the marrow cavity of the right tibia. After 20 days, the mice were sacrificed, and the tumors were isolated. The tumor volume was estimated by the diameter difference between ipsilateral and contralateral hind limbs.

For the in vivo assay, all chemical reagents were prepared in appropriate solution according to the manufacturer’s protocol. Their concentrations were indicated in our results. Treatment with chemical reagents began on day 7 after cell injection. Twelve tumor burden mice (glioma, *n* = 6; OS, *n* = 6) were treated with RR-11a (20 mg/kg BW, daily, i.p.). All the aforementioned mice and other mice xenografted with our stable transfected cell lines were treated with cisplatin (4 mg/kg BW, twice a week, i.p.), and negative control (NC) group was treated with normal saline solution.

### Statistical analysis

Expression and survival analyses of patients with glioma and breast cancer were performed by Gene Expression Profiling Interactive Analysis (GEPIA), a web server for gene expression profiling in tumor and normal tissue and interactive analyses. Correlation analysis of the expression of two genes was also performed with GEPIA.

Statistical analyses were performed using IBM SPSS Statistics version 21.0 (IBM Corp., Armonk, NY, USA). Graphs were generated using GraphPad Prism 9 (GraphPad Software Inc., San Diego, CA, USA). Two-tailed Student’s *t* test, one-way analysis of variance (ANOVA), Pearson correlation analysis, Kaplan‒Meier analysis, and log-rank tests were performed to analyze the corresponding data. A two-tailed *P* value < 0.05 was considered to indicate a significant difference. Grayscale analysis of WB bands and profiling and quantification of fluorescence intensity in fluorescence images were performed using Fiji (version 1.54 f).

## Supplementary information

Supplementary figures

Supplement Table

Supplementary methods

Supplement Table data

WB original data

Graphic Abstract

U2OS STR

143B STR

U87 STR

A549 STR

## Data Availability

All data associated with this study are present in the paper. PAR-CLIP-sequencing data of the G3BP1-FL, tG3BP1-C1, tG3BP1-C2 of U2OS cell have been deposited in the Sequence Read Archive (SRA) [https://submit.ncbi.nlm.nih. gov/subs/sra/] under the accession code PRJNA1149923.
