# Supplementary material for: Chemotherapeutic drug-triggered AEP-cleaved G3BP1 orchestrates stress granules/nucleoli/mitochondria in osteosarcoma
Source: Bone Res. 2025 Aug 26;13:74. doi: 10.1038/s41413-025-00453-w (PMC12381239; doi:10.1038/s41413-025-00453-w)
Supplement: Supplementary file 1 — Supplementary figures [file 41413_2025_453_MOESM1_ESM.docx]

**
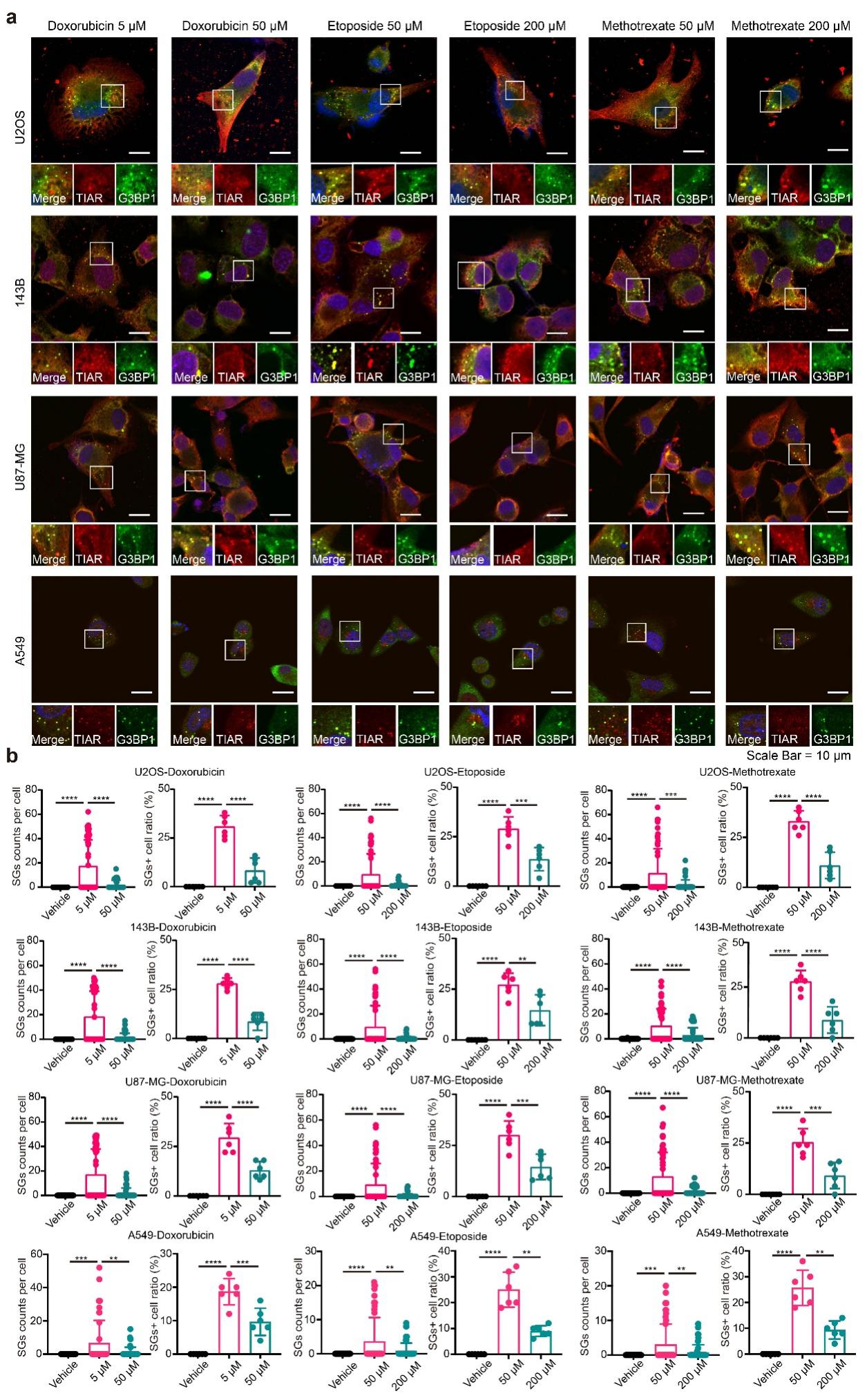
Supplemental Figures and figure legends**

**Fig. S1** **chemotherapeutic drugs induce SGs in cancer cells.** **a** Representative images of SGs assembly in U2OS, 143B, U87-MG and A549 cell lines exposed to various concentrations of doxorubicin, etoposide, or methotrexate for 6 hours. Vehicle groups were not shown in this panel (see **Fig. 1a**). **b** Quantification of the counts of SGs per cell (*n* = 50) and SGs+ cell ratio (*n* = 6) in cells of (**a**). Data are mean ± SD. **P<0.01, ***P <0.001, ****P < 0.0001. One-way ANOVA.


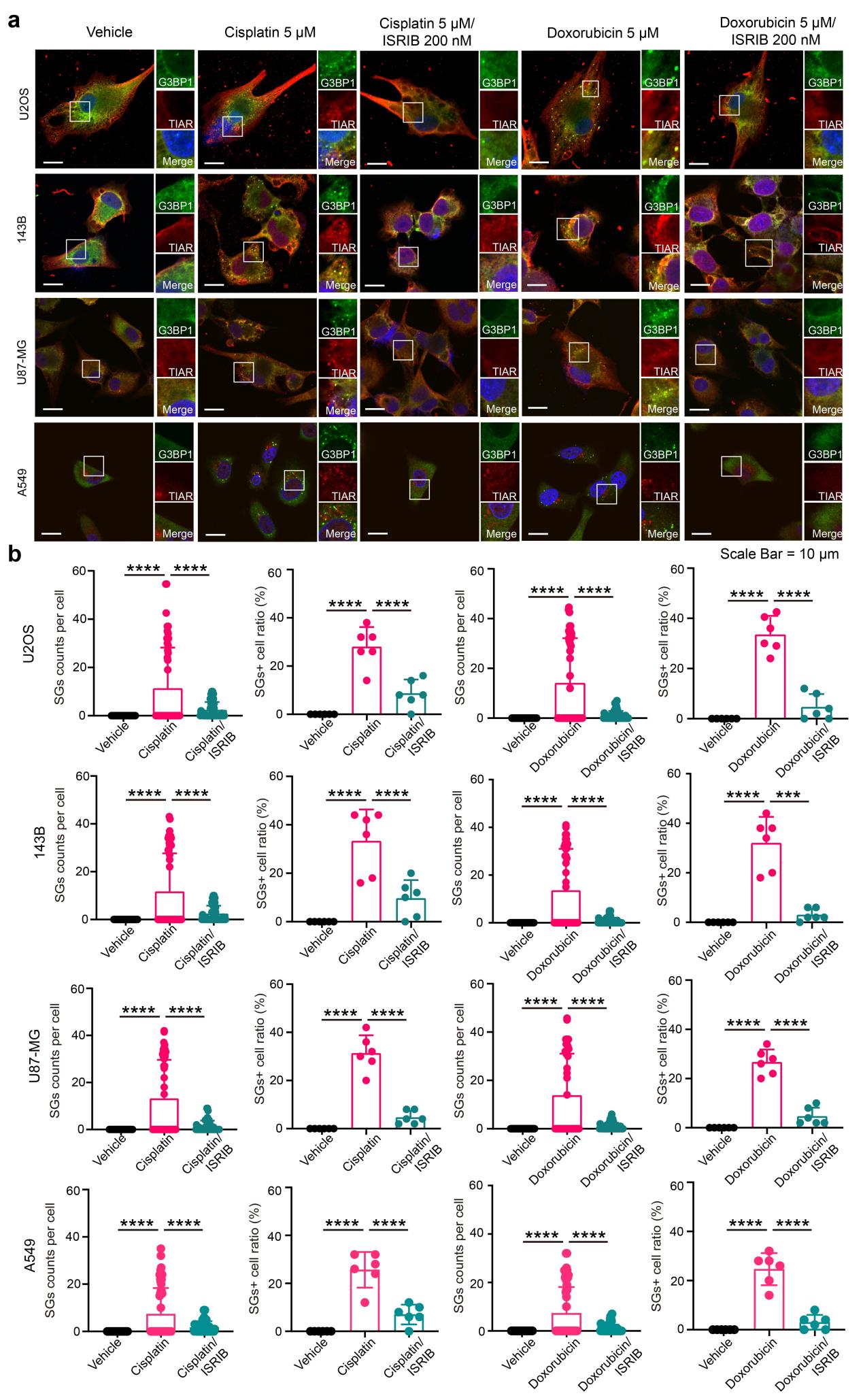


**Fig. S2 ISRIB inhibits the SGs induced by chemotherapeutic drugs in cancer cells. a** Representative images of SGs assembly in U2OS, 143B, U87-MG and A549 cells exposed vehicle, cisplatin (5 μM) or doxorubicin (5 μM) combined with ISRIB (200 nM) or vehicle for 6 hours. Scale Bar = 10 μm. **b** Quantification of the counts of SGs per cell (*n* = 50) and SGs+ cells ratio (*n* = 6) in cells of (**a**). Data are mean ± SD. ***P < 0.001, ****P < 0.0001. One-way ANOVA.

**
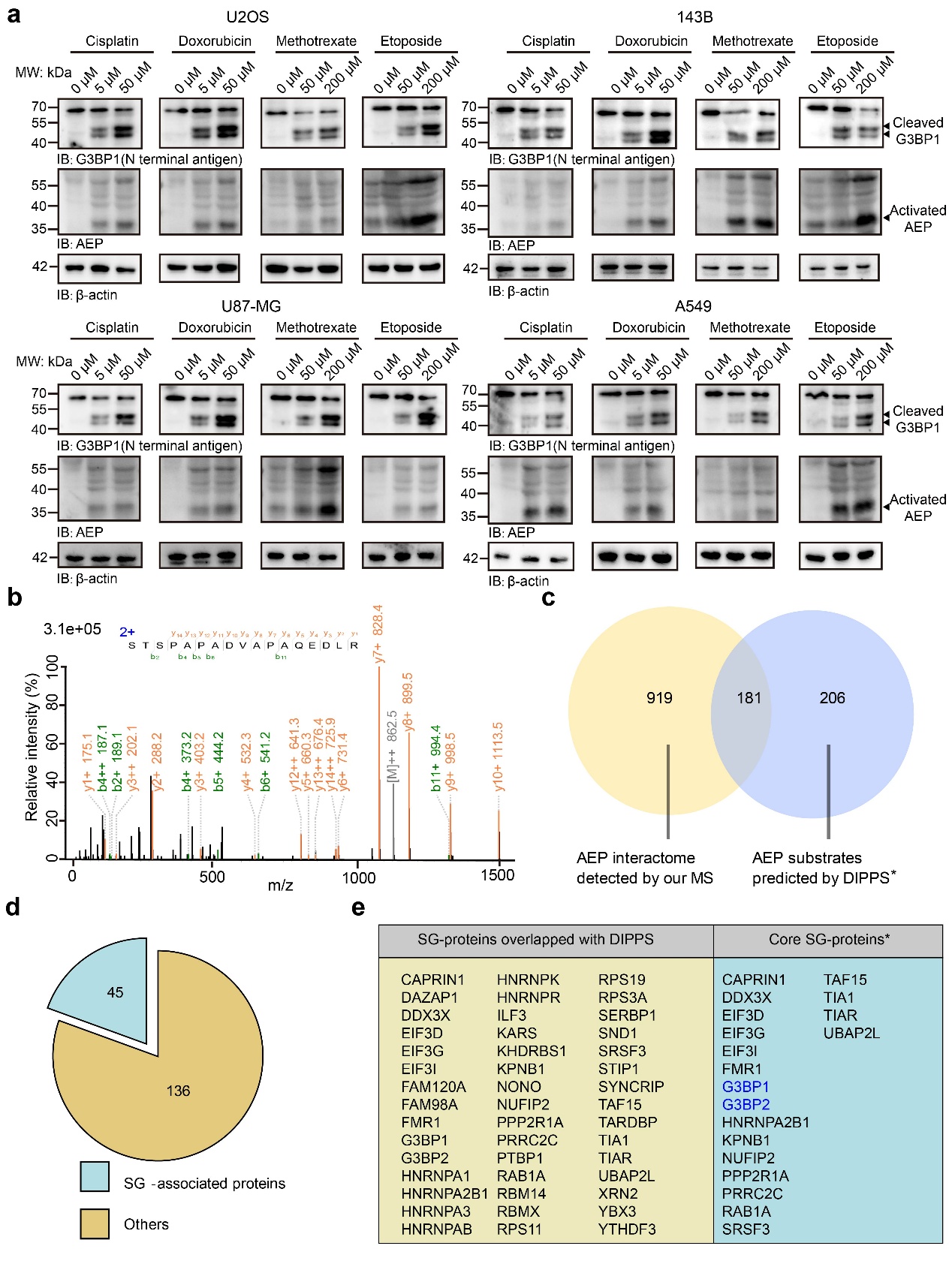
Fig. S3 The chemotherapeutic drugs induces the activation of AEP and cleaves G3BP1. a** WB analysis revealed AEP activation and corresponding G3BP1 cleavage patterns across gradient drug concentrations for 6 hours. **b** The acquired LC-MS/MS spectrum of G3BP1 peptides. **c** Venn diagram of the AEP interactome identified by our MS analysis and AEP substrates predicted by DIPPS*. **d** Pie chart showing the overlapping SG components in (**c**). **e** Detailed SG proteins mentioned above.

**
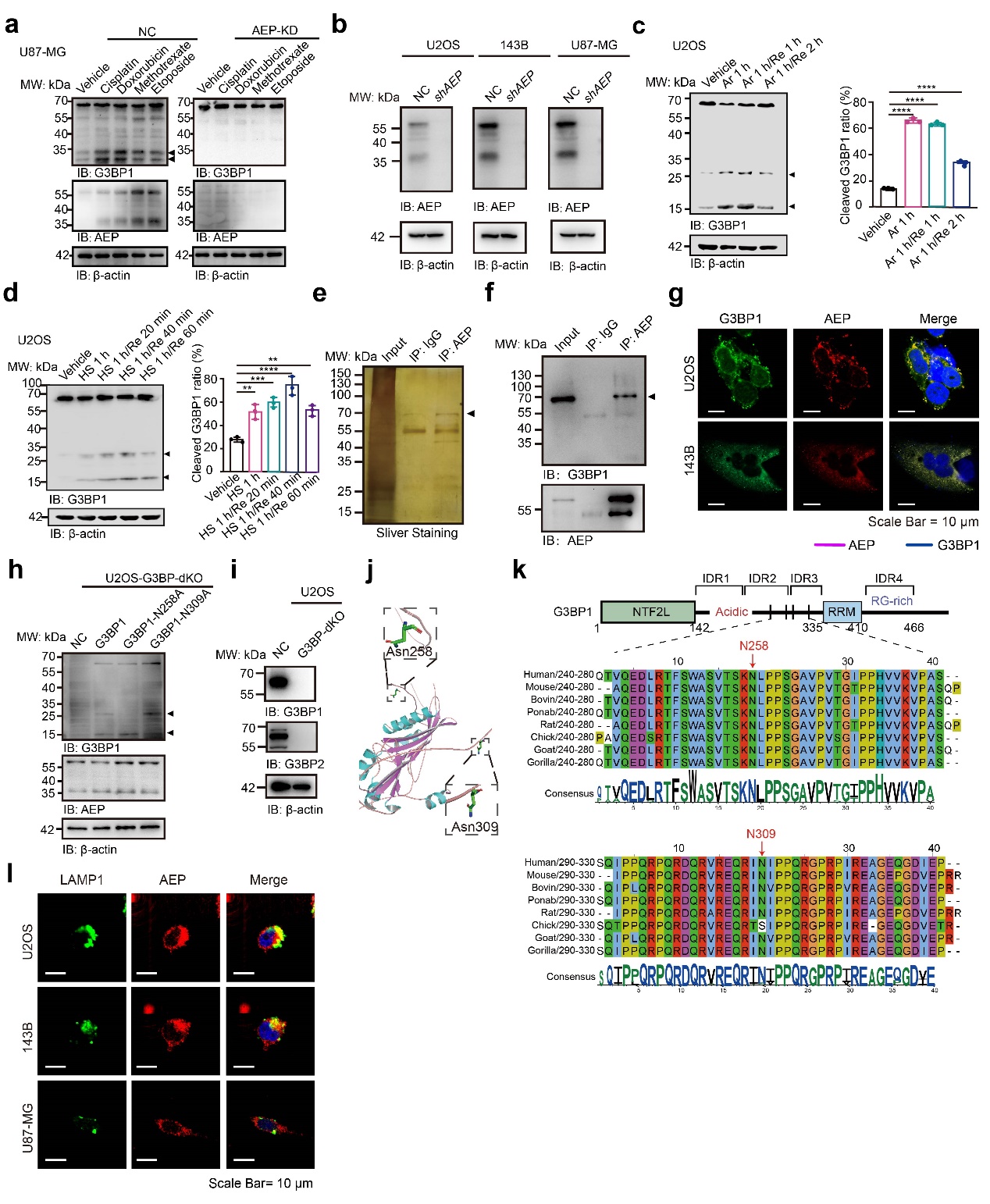
Fig. S4 AEP binds to and cleaves G3BP1 at N258 and N309. a** WB analysis of G3BP1 and AEP in U87-MG with NC or AEP-knockdown (KD) exposed to different chemotherapeutic drugs for 6 hours. **b** WB analysis of AEP knockdown in U2OS, 143B and U87-MG cells. **c** WB analysis and quantification of G3BP1 cleavage at various time points during sodium arsenite treatment and recovery (*n* = 3). **d** WB analysis and quantification of G3BP1 cleavage at various time points during heat shock and recovery (*n* = 3). **e** Co-IP and Sliver Stain analysis of interaction between AEP and G3BP1. **f** Co-IP and WB analysis of interaction between AEP and G3BP1. **g** Representative images of colocalization between AEP and G3BP1 detected in OS cells exposed to cisplatin (50 μM) for 6 hours. Scale Bar = 10 μm. **h** WB analysis of G3BP1 cleavage by AEP in G3BP1/G3BP2 double-knockout (dKO) U2OS cells rescued with G3BP1-WT, G3BP1-N258A, G3BP1-N309A. **i** Validation of knock out of G3BP1 and G3BP2 in U2OS. **j** Structure diagram of G3BP1, emulated domains of G3BP1 interacting with the AEP enzymatic centre. (Human G3BP1 UniProt code: Q13283)**. k** Evolutionary conservation analysis of cleavage sites of G3BP1 in multiple species. **l** IF analysis localization of AEP and LAMP1 (lysosome marker) in U2OS, 143B, and U87-MG cells. Scale Bar = 10 μm. Data are mean ± SD. **P < 0.01, ***P < 0.001, ****P < 0.0001. One-way ANOVA.


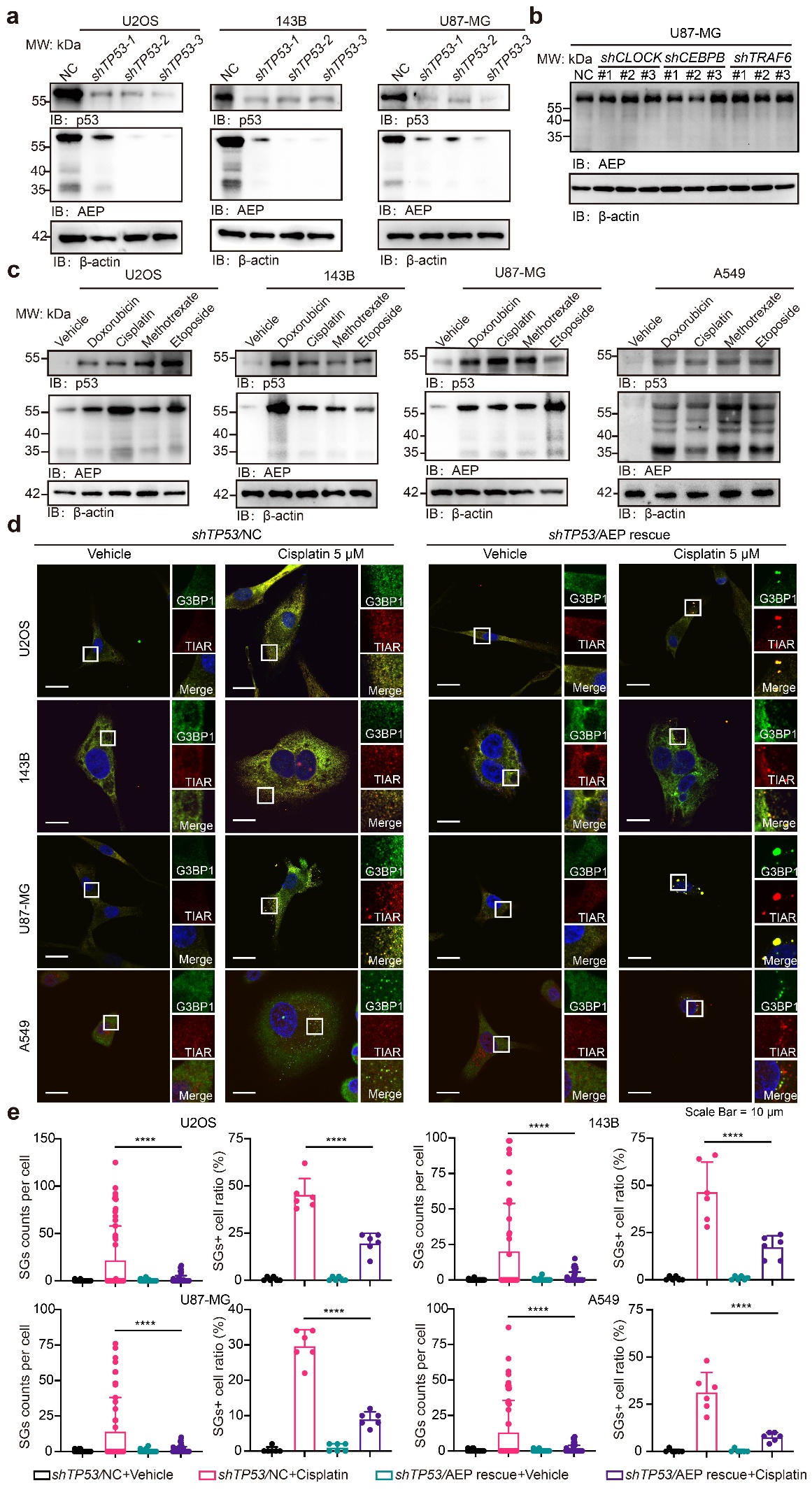


**Fig. S5 Knockdown of p53 decreased the expression of AEP. a** WB analysis expression of AEP and p53 in p53-KD U2OS, 143B and U87-MG cells. **b** WB analysis expression of AEP in CLOCK, CEBPB or TRAF6-KD U2OS, 143B and U87-MG cells. **c** The influence of cisplatin (50 µM, 6 hours), doxorubicin (50 μM, 6 hours), etoposide (200 μM, 6 hours), methotrexate (200 μM, 6 hours) on expression and activation of AEP. **d** Representative images of SGs in p53-KD/NC and p53-KD/AEP rescue U2OS, 143B, U87-MG and A549 cells exposed cisplatin (5 μM) or vehicle for 6 hours. Scale Bar = 10 μm. **e** Quantification of the counts of SGs per cell (*n* = 50) and SGs+ cell ratio (*n* = 6) in cells of (**d**). Data are mean ± SD. ****P < 0.0001. One-way ANOVA.

**
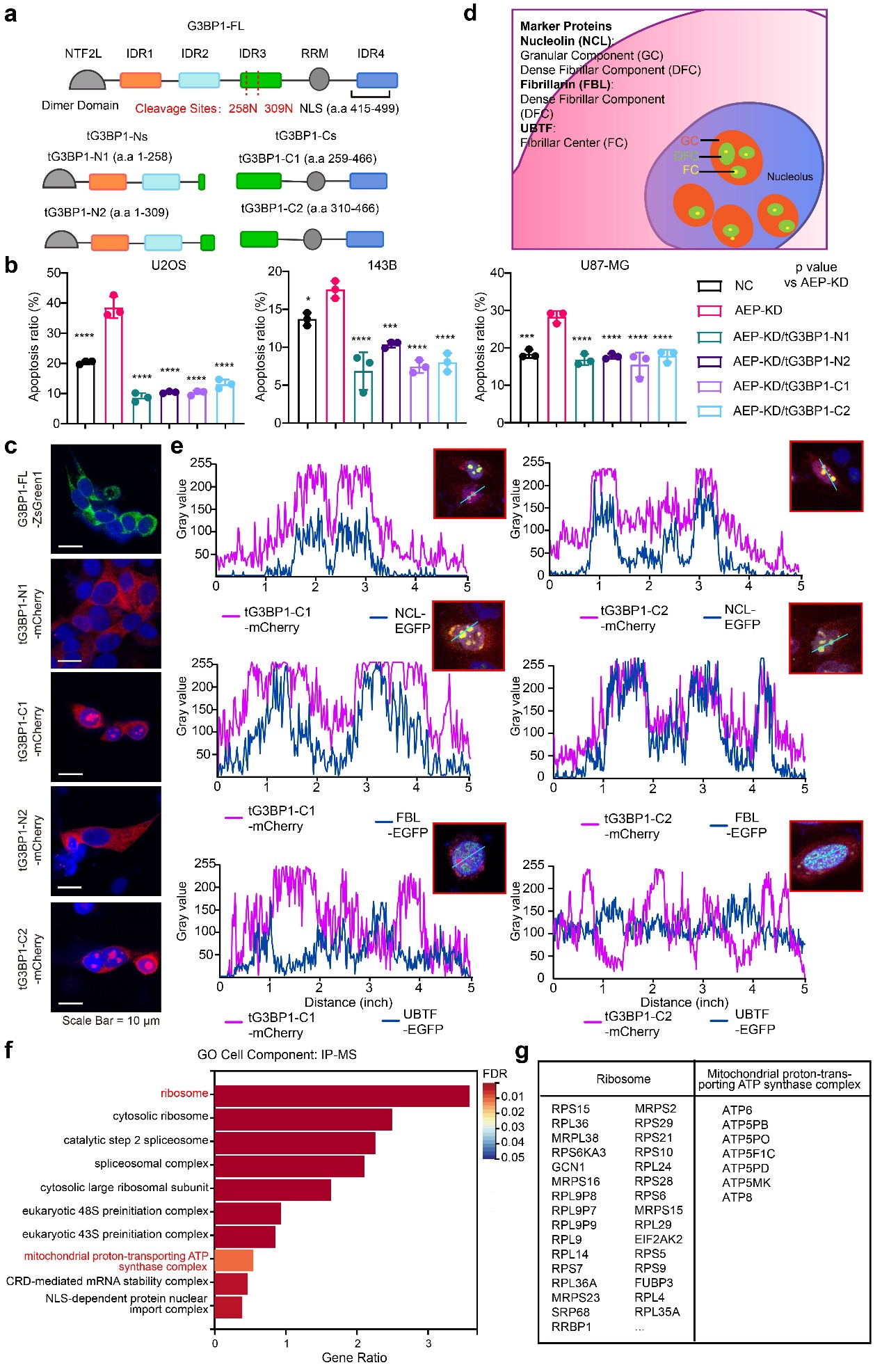
Fig. S6 tG3BP1-Cs translocated into nucleolus. a** 2D schematic diagram illustrating G3BP1 domains and cleavage. **b** Apoptotic cells (*n* = 3) induced by cisplatin (50 μM, 6 hours) were measured in U2OS, 143B, and U87-MG cell lines. **c** Representative image showing the subcellular localization of tG3BP1-Ns and tG3BP1-Cs in U2OS cells, Scale Bar = 10 μm. **d** Structure diagram of the nucleolus with marker proteins for the granular component (GC), dense fibrillar component (DFC) and fibrillar centre (FC). **e** Plot Profiling analysis of colocalization of mCherry-tagged tG3BP1-Cs and GFP-tagged sub-nucleolar marker proteins in **Fig. 3a.** **f and g** Mass spectrometry analysis of upregulated proteins interacting with tG3BP1-Cs compared to IgG. Data are mean ± SD. *P < 0.05, ***P < 0.001, ****P < 0.0001. One-way ANOVA.


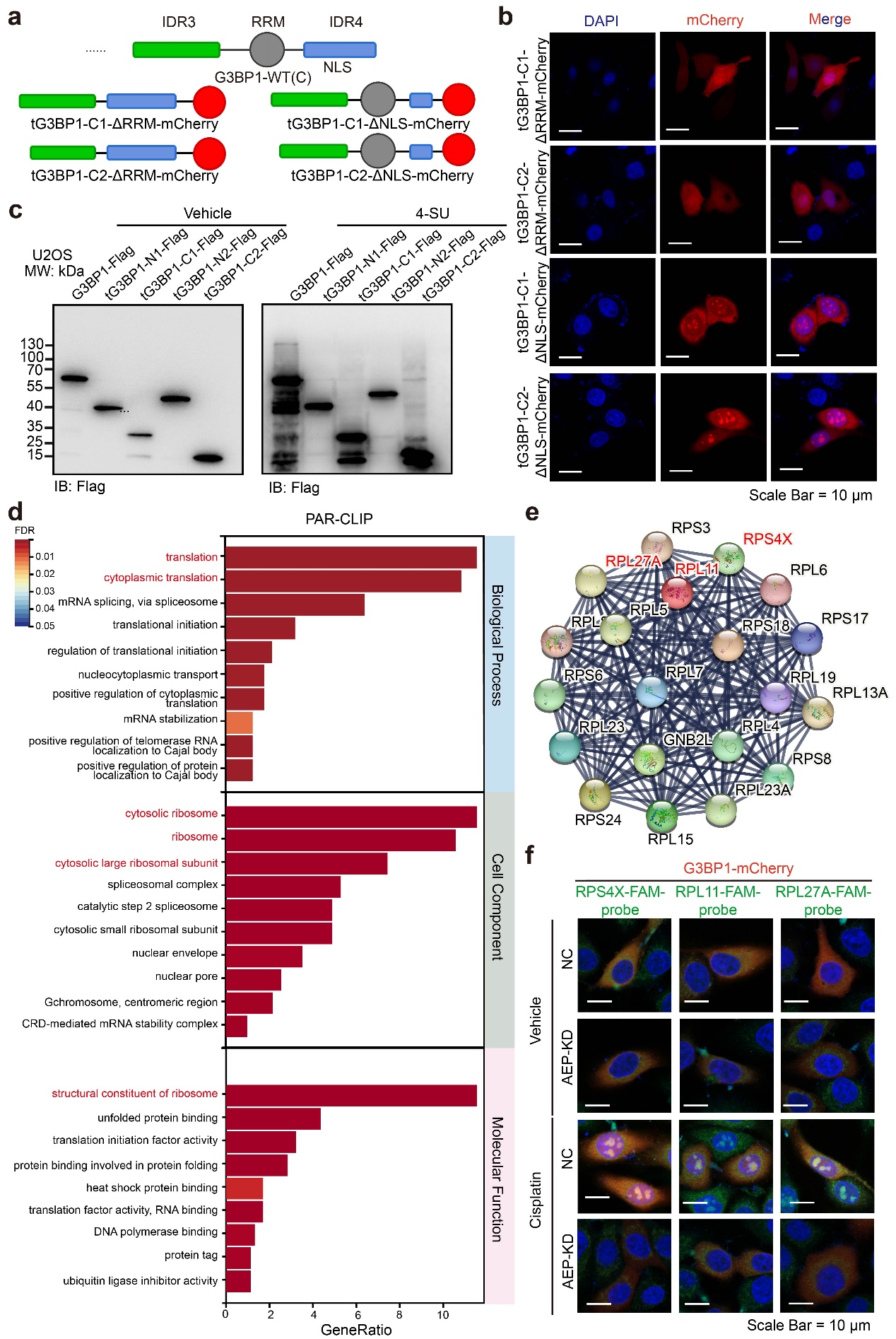
**Fig. S7 tG3BP1-Cs bind mRNA of ribosomal and mitochondrial. a** 2D structure diagram of tG3BP1-Cs as well as the RRM and NLS domain deletion mutants. **b** Representative images of localization of mCherry-tagged tG3BP1-Cs and the RRM and NLS domain deletion mutants in the nucleolus in HeLa cells, Scale Bar = 10 μm. **c** PAR-CLIP experiments in U2OS cells expressing G3BP1-Flag or truncates. Smear bands were observed in 4-SU group indicating the binding of RNA to G3BP1 and tG3BP1-Cs. **d** Gene ontology analysis of mRNAs with differential bond of tG3BP1-Cs. **e** PPI diagram showing the tG3BP1-C-bound RNAs involved in the *ribosome pathway*. **f** Representative images of tG3BP1-Cs translocation into the nucleolus induced by cisplatin (50 µM) or vehicle solution for 6 hours in NC and AEP-KD U2OS, 143B and U87-MG cell lines, Scale Bar = 10 μm.


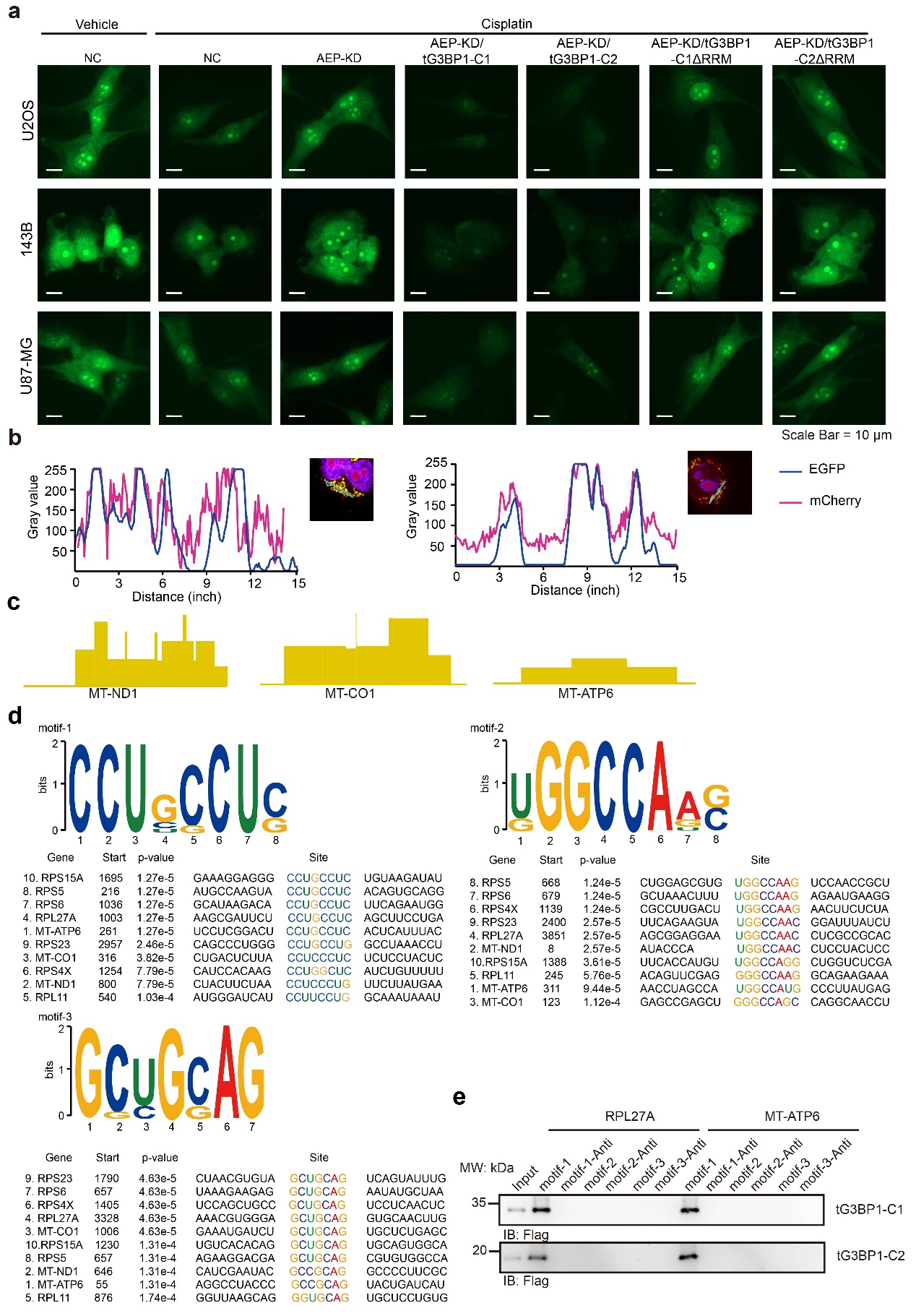
**Fig. S8 Predicted motifs of target mRNA binding to tG3BP1. a** Protein synthesis of the aforementioned cell lines exposed to cisplatin (50 μM) or vehicle for 6 hours were detected with the Click-iT HPG system. **b** Plot profiling analysis of colocalization of tG3BP1-Cs and TOMM20-mCherry in U2OS **Fig. 4a**. **c** Sequencing read peak profile of miRNA binding to tG3BP1-Cs. **d** Detailed information of potential motif that may dominate the binding between target mRNA and tG3BP1-Cs. **e** RNA pulldown and WB analysis of binding between candidate motif and tG3BP1-Cs.

**
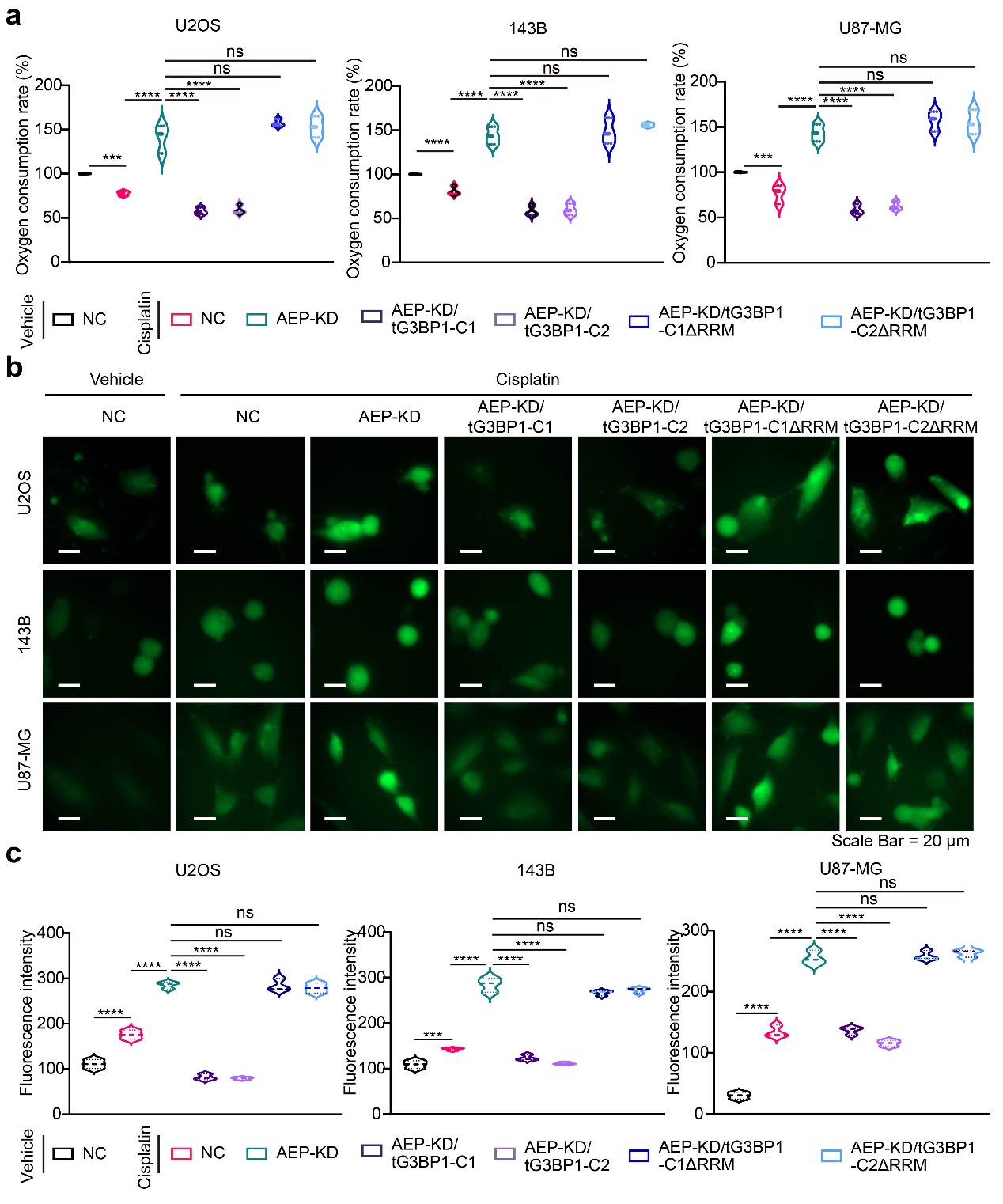
****Fig. S9 tG3BP1-Cs reduced mitochondrial-damage caused by cisplatin. a** The oxygen consumption rate (OCR) was analyzed in cells as indicated (*n*=3). **b** ROS production analysis of cell lines exposed to cisplatin (50 µM) or vehicle for 6 hours. **c** Quantitative analysis of cisplatin-induced ROS expression of (**b)** (*n* = 3). Data are mean ± SD. ***P < 0.001, ****P < 0.0001. ns: no significance. One-way ANOVA.


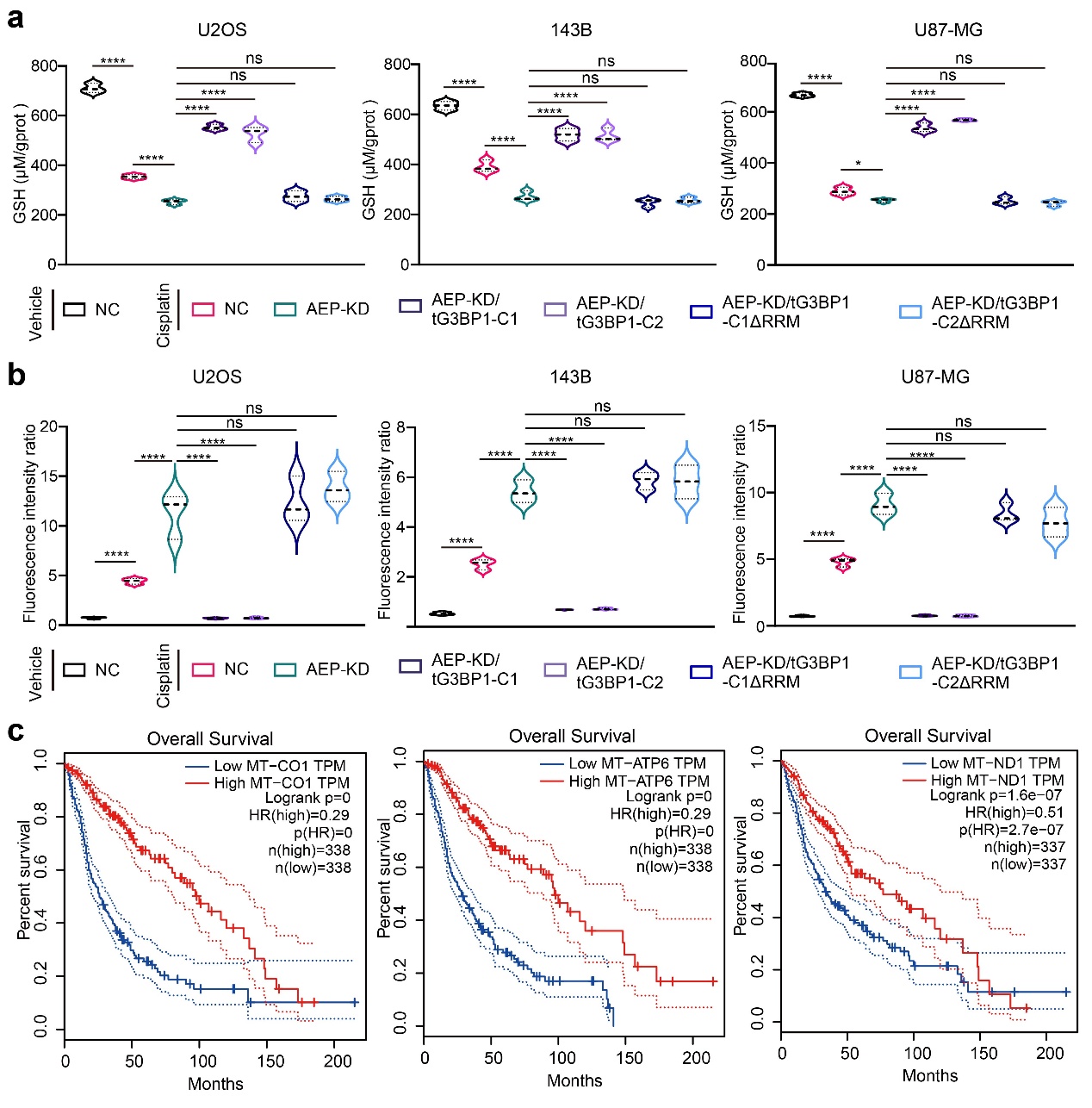
**Fig. S10 tG3BP1-Cs reduced mitochondrial-damage caused by cisplatin. a** Quantitative analysis of cisplatin (50 μM, 6 hours)-induced GSH consumption in cells as indicated (*n* = 3). **b** Quantitative analysis of cisplatin (50 μM, 6 hours)-induced mitochondrial damage estimated by JC-1 staining (*n* = 3). **c** Database analysis of the relationships between the expression levels of cuproptosis-related genes and survival in patients with glioma. Data are mean ± SD. *P < 0.05, ****P < 0.0001. ns: no significance. One-way ANOVA.


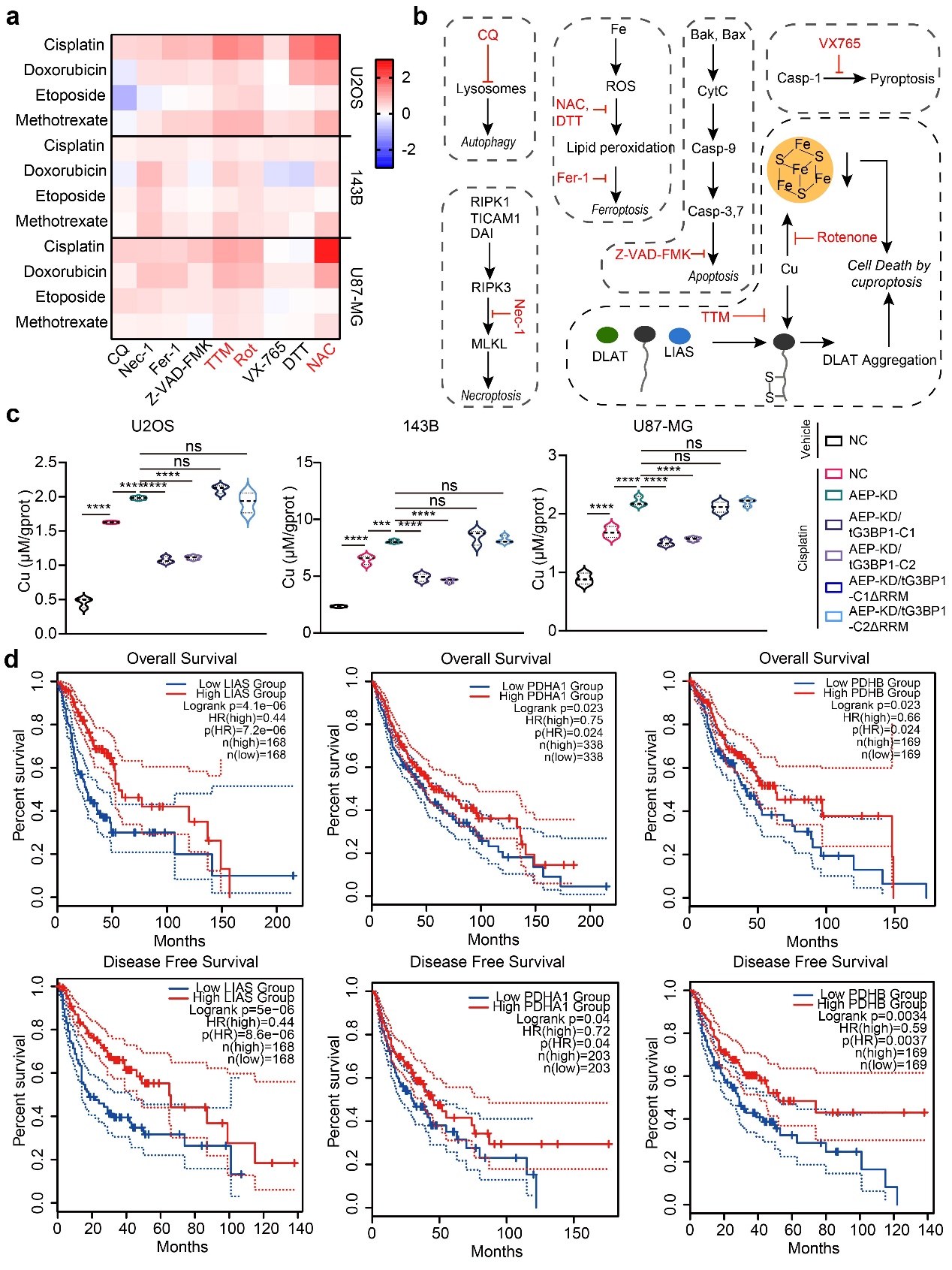
**Fig. S11 Chemotherapeutic drugs induced tumor cells cuproptosis. a** Heatmap of viability of cells pretreated overnight with chloroquine (CQ) (1 μM), necrostatin-1 (Nec-1) (20 μM), ferrostatin-1 (Fer-1) (10 μM), Z-VAD-FMK (30 μM), TTM (20 μM), Rotenone (Rot) (300 nM), Belnacasan (VX765) (500 nM), DTT (1 mM), N-acetylcysteine (NAC) (5 mM) and then treaded cisplatin (50 μM)，doxorubicin (50 μM)，etoposide (200 μM)，methotrexate (200 μM) (*n* = 3). **b** Schematic diagram of inhibitors targeting autophagy, necroptosis, ferroptosis, apoptosis, pyroptosis or cuproptosis. **c** Quantitative analysis of cisplatin (50 μM, 6 hours)-induced Cu in U2OS, 143B and U87-MG cells with AEP knockdown and rescued with tG3BP1-Cs or tG3BP1-CΔRRMs (*n* = 3). **d** TCGA database analysis of the relationships between the expression levels of cuproptosis-related genes and survival in patients with glioma and sarcoma. Data are mean ± SD. ***P < 0.001, ****P < 0.0001. ns: no significance. One-way ANOVA.


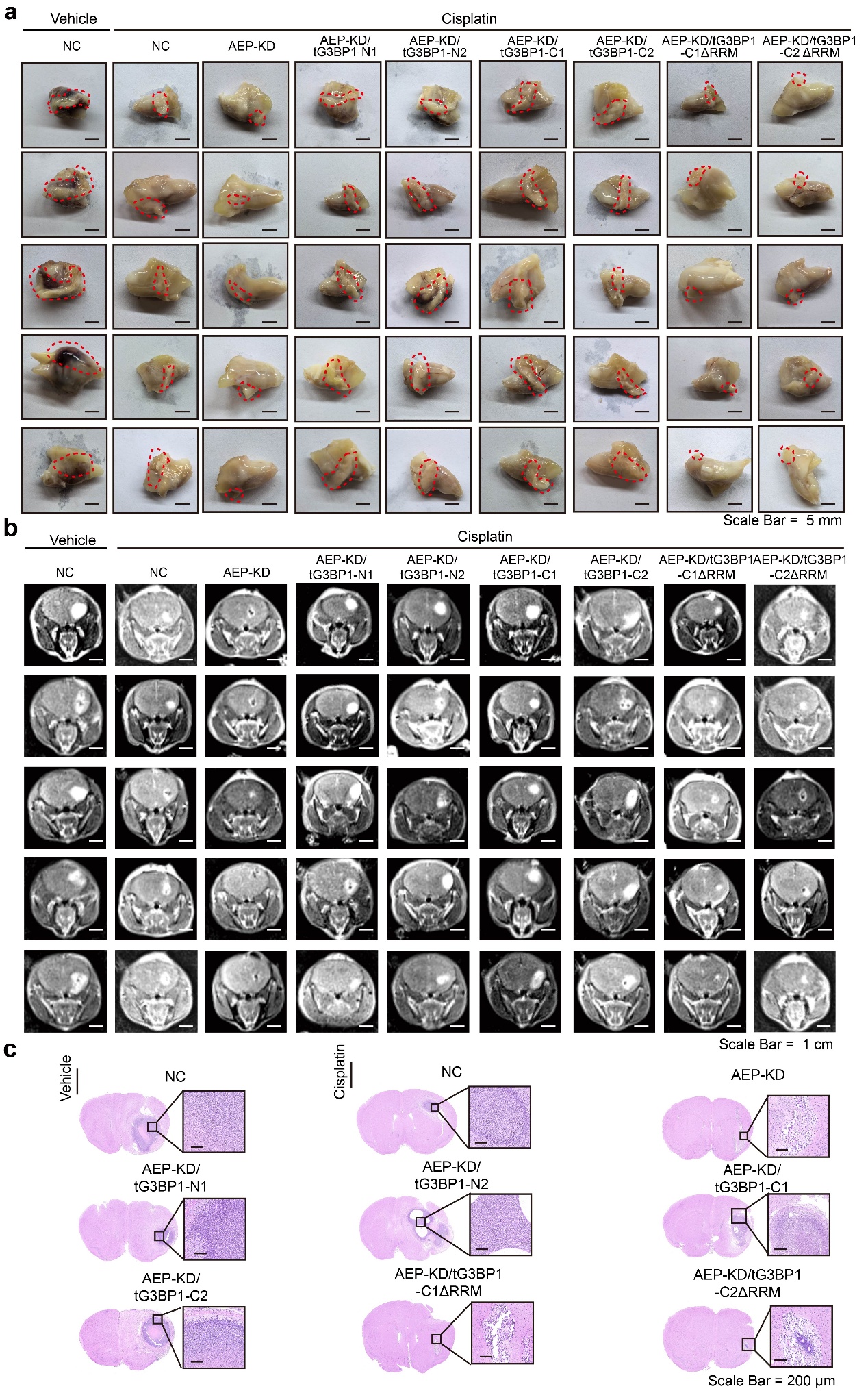
**Fig. S12 Macroscopic images, MRI and H&E images of xenografts mouse model. a** Macroscopic images of osteosarcoma xenografts mouse model of **Fig. 5b**. **b** Representative MRI images of the xenograft mouse model that were injected with the aforementioned U87-MG cell lines and treated with cisplatin or vehicle. **c** Representative H&E images of tumors in the xenograft model of (**b)**.


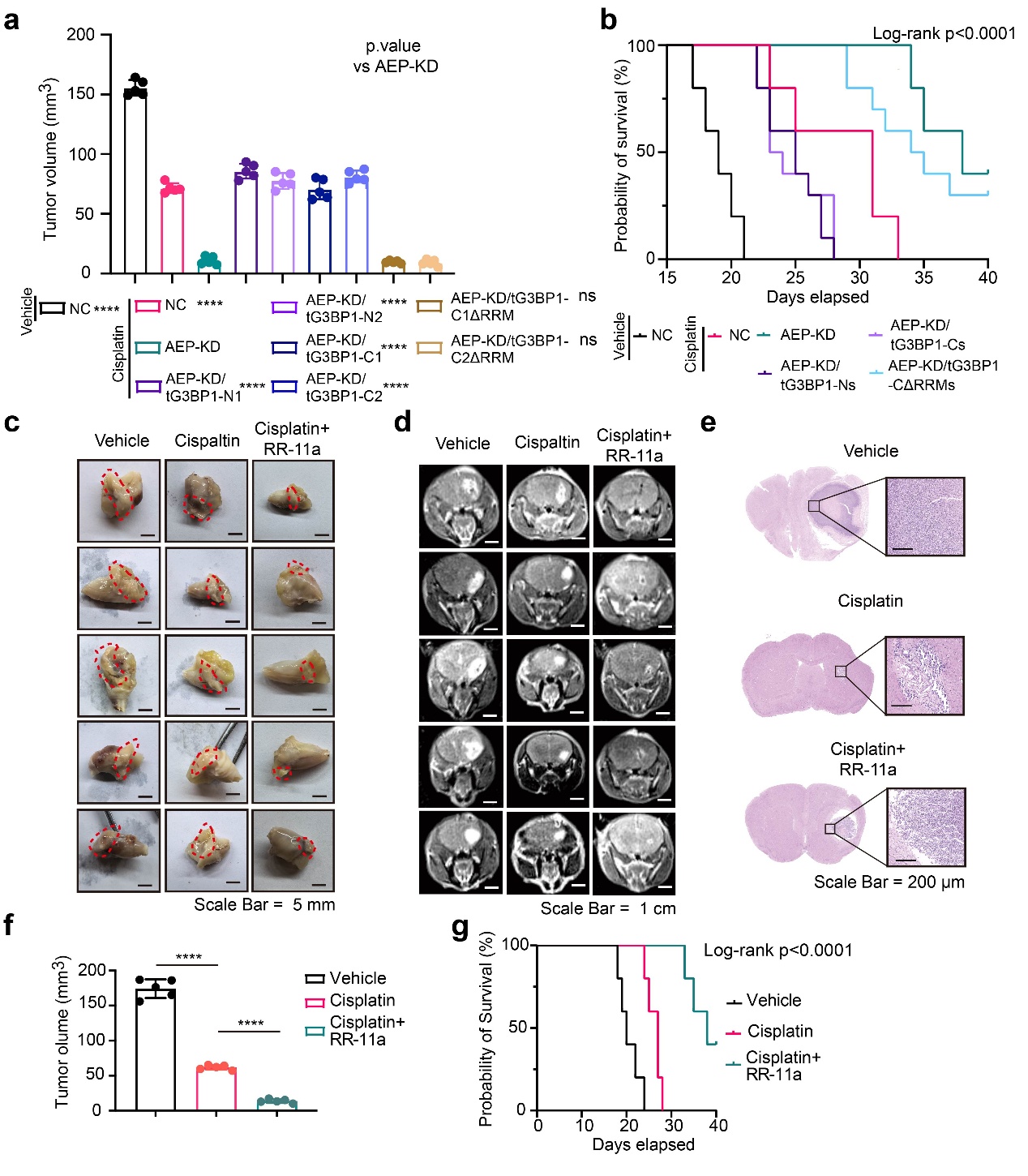
**Fig. S13 In situ glioma mouse models validate that AEP and truncated G3BP1 promote tumor drug-resistance. a** Quantification of the tumor volume from the xenograft mouse model implanted with AEP-KD, AEP-KD/tG3BP1-Cs rescue, AEP-KD/tG3BP1-CsΔRRMs rescue, and negative control U87-MG cells (*n*=5). **b** Kaplan‒Meier survival curves for the abovementioned glioma-bearing mice (*n*=5). log-rank (Mantel–Cox) test, P < 0.0001. **c** Macroscopic images of osteosarcoma xenografts mouse model of **Fig. 5e**. **d** Representative MRI images of the xenograft mouse model implanted with U87-MG cells and treated with cisplatin (4 mg/kg) and/or RR-11a (20 mg/kg). **e** Representative H&E images of tumors in the xenograft mouse model of (**d**). **f** Quantification of tumor volume in the xenograft mouse model in (**d**) (*n*=5). **g** Kaplan‒Meier survival curve for the abovementioned xenograft mouse model (*n*=5). Log-rank (Mantel-Cox) test, P <0.0001. Data are mean ± SD. ****P < 0.0001. ns: no significance.

**
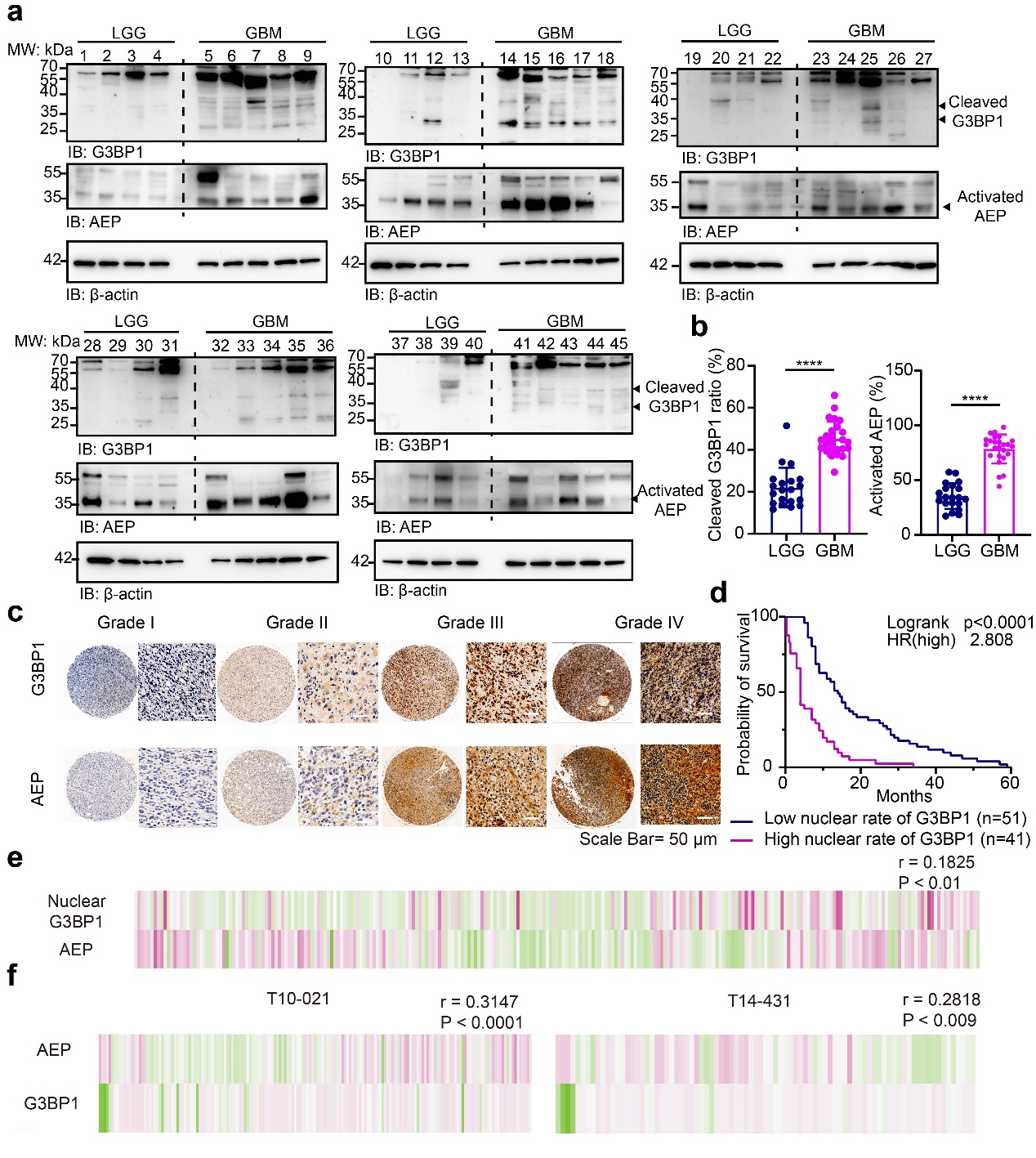
Fig. S14 Clinical specimens from patients with GBM analysis of expression and prognostic value of AEP and G3BP1. a and b** WB analysis and quantification of G3BP1 cleavage and AEP activation in low-grade glioma (LGG, *n* = 20) and GBM (*n* = 25) clinical tumor tissues. unpaired Student’s *t* test. **c** Representative IHC images of microarrays of tumor tissues of different grades from patients with glioma. **d** Kaplan‒Meier survival curves for patients with a low proportion of G3BP1 nuclear localization (*n*=51) and patients with a high proportion of nuclear localization (*n*=49). log-rank (Mantel-Cox) test. **e** Heatmap visualization and quantitative relationships between the nuclear localization of G3BP1 and AEP expression in tumor tissue microarrays. **f** Heatmap visualization and quantitative relationships of G3BP1 and AEP expression in tumor tissue microarrays. Data are expressed as mean ± SD. ****P < 0.0001.
