# Supplementary material for: Chemotherapeutic drug-triggered AEP-cleaved G3BP1 orchestrates stress granules/nucleoli/mitochondria in osteosarcoma
Source: Bone Res. 2025 Aug 26;13:74. doi: 10.1038/s41413-025-00453-w (PMC12381239; doi:10.1038/s41413-025-00453-w)
Supplement: Supplementary file 3 — Supplementary methods [file 41413_2025_453_MOESM3_ESM.docx]

**Cell lines**

The human embryonic kidney cell line HEK 293T and tumor cell lines U2OS (human osteosarcoma cell), 143B (human osteosarcoma [OS] cell), and U87-MG (human glioma cell) were obtained from the American Type Culture Collection (ATCC, VA, USA) with the following identifiers: HEK293T (Cat# CRL-3216, RRID: CVCL_0063), HeLa (Cat# CCL-2, CVCL_0030), human U87-MG (Cat# HTB-14, RRID: CVCL_0022), human U2OS (Cat# HTB-96, RRID: CVCL_0042), human 143B (Cat# CRL-8303, RRID: CVCL_2270) and human A549 (Cat# CRM-CCL-185, RRID: CVCL-0023).

All cells were maintained in modified Eagle’s medium (MEM) (Cat# 11095080, Gibco, USA), Dulbecco’s modified Eagle’s medium (DMEM) (Cat# 11965-092, Gibco, USA) or RPMI-1640 medium (Cat# SH30809.01, HyClone, Shanghai, China) containing 10% fetal bovine serum (FBS, Cat# 10270-106, Gibco, USA) and 100 IU/mL penicillin/streptomycin (Cat# P1400, Solarbio, Beijing, China) at 37°C in a humidified incubator with 5% CO_2_.

***Escherichia coli* competent cells**

DH5α (Cat# B528413, Sangon Biotech Co., Ltd., Shanghai, China) were used for plasmid amplification. Rosetta (DE3) Super Competent Cells (Cat# D1065M, Beyotime, Shanghai, China) were used for the prokaryotic expression of recombinant G3BP1 (WT and N258A, N309A point mutants).

**Antibodies**

The primary antibodies are as follows: rabbit monoclonal anti-G3BP1 C terminal antigen (Cat# CY8201, Antigen epitope: REGDRRDNRLRG, Abways, Shanghai, China), anti-G3BP1 N terminal antigen (Cat# 66486-1-PBS, Antigen epitope: RPQRDQRVREQR, Proteintech, Wuhan, China), mouse monoclonal anti-Flag (Cat# AB0008, Abways), TIAR monoclonal antibody (Cat# 66907-1-Ig, Proteintech, Wuhan, China), human anti-legumin/asparaginyl endopeptidase (Cat# AF2199, R&D Systems, MN, USA), rabbit monoclonal anti-mCherry (Cat# ab213511, Abcam, Cambridge, UK), mouse anti-P53 (Cat# sc-126, Santa Cruz Biotechnology, USA), anti-puromycin, clone 12D10 (Cat# MABE343, Millipore, Billerica, USA), polyclonal anti-ND1 (Cat# 19703-1-AP, Proteintech), rabbit anti-RPS4X (Cat#AY3996, Abways), rabbit anti-RPL11 (Cat#AY3963, Abways), rabbit anti-RPL27A (Cat#AY3971, Abways), ATP6 monoclonal antibody (Cat# 68442-1-Ig, Proteintech), and MTCO1 Rabbit mAb (Cat# A24805, ABclonal, Wuhan, China).

The horseradish peroxidase (HRP)-conjugated antibodies used for Western blotting (WB) were as follows: HRP goat anti-rabbit IgG (H+L) (Cat# AS014, ABclonal), HRP goat anti-mouse IgG (H+L) (Cat# AS003, ABclonal), HRP rabbit anti-goat IgG (H+L) (Cat# AS029, ABclonal), and HRP conjugated anti-beta actin (Cat# AB2001, Abways).

The secondary antibodies used for immunofluorescence (IF) and purchased from Thermo Fisher Scientific (MA, USA) were as follows: Alexa Fluor Plus 488 Donkey anti-goat (Cat# A32814), Alexa Fluor Plus 488 Donkey anti-rabbit (Cat# A32790), Alexa Fluor Plus 488 Donkey anti-mouse (Cat# A32766), Alexa Fluor Plus 594 Donkey anti-goat (Cat# A32758,), Alexa Fluor Plus 594 Donkey anti-rabbit (Cat# A32754), and Alexa Fluor Plus 594 Donkey anti-mouse (Cat# A32744).

**Chemicals and reagents**

The chemotherapeutic drugs and other compounds used in this study were listed as follows: cisplatin (Cat# HY-17394, MedChemExpress, Shanghai, China), doxorubicin (Cat# HY-15142A, MedChemExpress), etoposide (Cat# HY-13629, MedChemExpress), methotrexate (Cat# HY-14519, MedChemExpress), chloroquine (Cat# HY-17589A, MedChemExpress,), necrostatin-1 (Cat# HY-15760, MedChemExpress), ferrostatin-1 (Cat# HY-100579, MedChemExpress), Z-VAD-FMK (Cat# HY-16658B, MedChemExpress), ammonium tetrathiomolybdate (Guillén-Boixet et al.) (Cat# 323446, Sigma‒Aldrich, Darmstadt, Germany), rotenone (Cat# HY-B1756, MedChemExpress), VX-765 (Cat# HY-13205, MedChemExpress), DL-dithiothreitol (DTT) (Cat# HY-15917, MedChemExpress), N-acetylcysteine (Cat# HY-B0215, MedChemExpress) 4-thiouridine (4-SU) (Cat# T4509, Sigma‒Aldrich), RR-11a (Cat# HY-112205, MedChemExpress), ISRIB (Cat# HY-12495A, MedChemExpress), N,N-dimethylformamide (Cat# HY-Y0345, MedChemExpress), and dimethyl sulfoxide (Cat# A610163, Sangon Biotech), Hieff Trans®Polyethylenimine Linear (PEI) MW40000 (Cat# 40816ES02, Yeasen, Shanghai, China), IPTG (Cat# I6758, Sigma-Aldrich, Missouri, USA), Ni-NTA His•Bind® Resin (Cat# 70666, Millipore, MA, USA), and recombinant human legumain (Cat#2199-CY, R&D, MN, USA).

**Fluorescent images**

Fluorescent images of IF, fluorescent protein-tagged cell or fluorescence in situ hybridization (FISH) were acquired using a laser scanning confocal microscope (Leica TCS SP8, Heidelberg, Germany) and analyzed with Fiji (Version 1.54f).

**Plasmids construction and transfection**

G3BP1 was polymerase chain reaction (PCR) amplified from cDNA obtained from HEK293T cells. Flag-tagged G3BP1 and its mutants, namely, tG3BP1-Ns, tG3BP1-Cs, and tG3BP1-CΔRRMs, were cloned and inserted into the pHY-023 vector (Hanyin, Shanghai, China). ZsGreen1-fused G3BP1-FL, Nucleolar Transcription Factor 1 (UBTF), Fibrillarin (FBL) and Nucleolin (NCL), mCherry-fused tG3BP1-Ns, tG3BP1-Cs, tG3BP1-CΔRRMs, and tG3BP1-CΔNLSs were inserted into the pHY-009 vector (Hanyin). shRNA targeting AEP was cloned and inserted into pLKO.1 TRC control (Addgene plasmid # 10879; http://n2t.net/addgene:10879; RRID: Addgene_10879). The primers and shRNA sequences are listed in Table S5. All constructs were extracted from transformed DH5α bacterial cells using an Endotoxin Free Plasmid Extraction Kit (Cat# DP108, Tiangen Biotech, Beijing) and verified by Sanger sequencing (Genewiz, Suzhou, China). To transiently express related proteins in HeLa cells, Lipofectamine 2000 (Cat# 11668019, Thermo Fisher Scientific) was used as described previously (Chen et al., 2022).

**Lentivirus preparation and stable cell line establishment**

Lentiviruses were packaged by the co-transfection of the targeting shRNA construct or overexpression construct with the envelope plasmid pMD2.G (Addgene plasmid # 12259; http://n2t.net/addgene:12259; RRID: Addgene_12259) and packaging plasmid psPAX2 (Addgene plasmid # 12260; http://n2t.net/addgene:12260; RRID: Addgene_12260) into HEK 293T cells. The virus-containing supernatant was harvested and filtered through a 0.45-µm polyvinylidene fluoride (PVDF) filter (Cat# SLHV033RB, Millipore, USA) and applied to the indicated cells in the presence of polybrene (5–10 µg/mL) (Cat# 40804ES76, Yeasen). After 72 h, stably transduced cells were selected with puromycin or blasticidin.

**G3BP1/2 dKO and associating rescue U2OS cell line construction**

For U2OS stable cell lines of G3BP1/2 dKO, gRNAs for G3BP1/G3BP2 were cloned into pLentiCRISPR V2, and the corresponding lentiviruses were packaged with HEK 293T. The lentivirus for G3BP1/G3BP2 dKO was added into the culture of U2OS with 10 µg/mL polybrene, and the DMEM culture medium was changed the next day. After 3 days, single cells were seeded into 96-well cell culture plate. The subclone cells were analyzed with WB for validation of the G3BP1/2 dKO. Then, G3BP1/2 dKO U2OS cells were rescued with G3BP1 WT, N258A, or N309A point mutants with the corresponding lentiviruses. The detailed sequences for gRNAs and G3BP1 primers are listed in Table S5.

**Silver staining**

The U2OS cells for sliver staining were seeded in 10-cm dish and treated with cisplatin (50 μM) for 6 hours, and the cells were collected for subsequent assay. For silver staining, the Quick Silver Staining Kit (Cat# P0017S, Beyotime) was used. The gels were fixed with the fixation solution for >20 mins, washed with 30% ethyl alcohol, washed again with Milli-Q-grade pure water or double distilled water, sensitized, washed twice with water, and subsequently stained with silver solution (1×) for 10 mins at RT. Then, the cells were washed with ddH2O for 1 min, silver staining solution was added, and the cells were incubated on a shaker at RT for 3–10 mins until the desired protein band appeared. Eventually, the reaction was stopped with a stop solution (1×), and images were captured.

**Recombinant G3BP1 protein purification**

The DNA of G3BP1 and truncates were acquired by reverse transcription of HEK 293T total RNA. Then, these DNA were cloned into pET28a (+) vector and transformed into DH5α competent cells. The correct plasmids were amplified and transformed by Rosetta (DE3) competent cells continuously. IPTG (1 mM) was used to induce the expression of recombinant G3BP1 (WT and point mutants). The recombinant proteins were purified from cell lysates with the Ni-NTA His•Bind® Resin according to the protocol (De Marco, 2007) for subsequent assays.

**Sequence conservation analysis**

Conservation analysis of G3BP1 was performed by multiple-sequence alignment analysis (<https://www.ebi.ac.uk/Tools/msa/clustalo/>).

**NLS prediction**

The nuclear localization signal (NLS) of G3BP1 was predicted using the online tool cNLS Mapper (https://nls-mapper.iab.keio.ac.jp/cgi-bin/NLS_Mapper_form.cgi).

**Quantitative reverse-transcription PCR (qRT‒PCR)**

Total RNAs were extracted from the cultured cells using TRIzol (Cat# R411-01, Vazyme, Nanjing, China) according to the manufacturer’s protocol. Reverse transcription was performed using a HiScript III 1st Strand cDNA Synthesis Kit (+gDNA wiper) (Cat# R312-01, Vazyme). QRT‒PCR was carried out on a Roche LightCycler® 480 thermal cycler with gene-specific primers (Table S5) and AceQ qPCR SYBR Green Master Mix (High ROX Premixed) (Cat# Q141-02, Vazyme). The ΔΔCT method was used for quantitative analysis, with GAPDH as the endogenous control.

**Cell glutathione (GSH) quantification**

Stable cell lines of U2OS, 143B, and U87-MG were seeded in 10-cm dish and treated with cisplatin (50 μM) or appropriate vehicle solution for 6 hours. The collected cells were added with the appropriate buffer, and GSH assay was performed with Reduced GSH Colorimetric Assay Kit (Cat# E-BC-K030, Elabscience) according to the manufacturer’s protocol.

**Cell copper (Cu) quantification**

Stable cell lines of U2OS, 143B, and U87-MG were seeded in a 10-cm dish and treated with cisplatin (50 μM) or appropriate vehicle solution for 6. The collected cells were added with the appropriate buffer, and Cu in cells was measured using Cell Copper (Cu2+) Colorimetric Assay Kit (Cat# E-BC-K775, Elabscience) according to the manufacturer’s protocol.

**Reactive oxygen species (ROS) detection**

Stable cell lines of U2OS, 143B, and U87-MG were seeded in 96-well cell culture plates and treated with cisplatin (50 μM) or appropriate vehicle solution for 6 hours. An ROS Kit (Cat# S0033S，Beyotime) was used to detect ROS production in cell lines according to the manufacturer’s protocol. The cell counting kit-8 assay was performed as described previously (Chen et al., 2022).

**Hematoxylin–eosin (H&E) staining**

Paraffin-embedded sections were stained with hematoxylin and eosin as indicated (Chen et al., 2022). After dehydration, neutral resin was used for sealing, and images were captured using a Pannoramic Scanner (Pannoramic DESK, P-MIDI, 3D HISTECH, Hungary).

**Immunohistochemistry (IHC)**

The expression of AEP and G3BP1 was examined by IHC using a human glioma tissue microarray (T10-021 and T14-431) containing 258 glioma samples from the Department of Neurosurgery, Renji Hospital, Shanghai Jiao Tong University School of Medicine. The microarray contained LGG (n = 59) and HGG (n = 199) samples. IHC staining was performed according to the protocol described previously (Chen et al., 2022). Anti-AEP (1:50) and anti-G3BP1 (1:200) antibodies were used. Images were captured by a Panoramic Scanner (Pannoramic DESK, P-MIDI, 3D HISTECH, Hungary). Aipathwell (Solarbio, Wuhan), a digital pathological image analysis software based on artificial intelligence learning, was used to analyze each sample in the tissue microarray. The H-index was used for the semiquantification of the staining intensity of each sample. The H-score formula was as follows: H-score =∑ (pi×i) = (percentage of weakly stained cells × 1) + (percentage of moderately stained cells × 2) + (percentage of strongly stained cells × 3). The percentage of positive cells with a given intensity (pi) was grouped as follows: 0%–5%, 6%–25%, 26%–50%, and >50%; i indicates the staining intensity, which was classified as follows: 0, no staining; 1, weak staining; 2, moderate staining; and 3, strong staining. The H-score was thus a value between 0 and 300; the higher the value, the stronger was the combined positive staining intensity. These scores were determined by two independent pathologists in a blinded manner.
