# Supplementary material for: Chemotherapeutic drug-triggered AEP-cleaved G3BP1 orchestrates stress granules/nucleoli/mitochondria in osteosarcoma
Source: Bone Res. 2025 Aug 26;13:74. doi: 10.1038/s41413-025-00453-w (PMC12381239; doi:10.1038/s41413-025-00453-w)

Figure 1c U2OS-WT IB:G3BP1

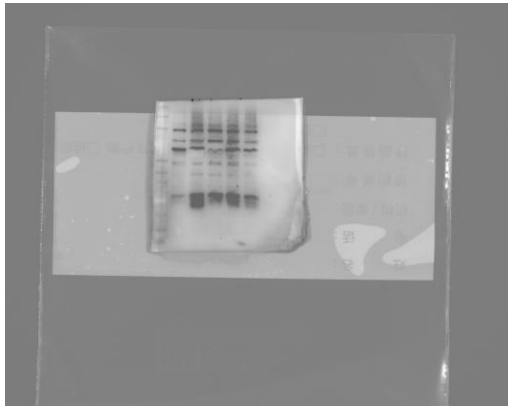

Figure 1c U2OS-AEP-KD IB:G3BP1

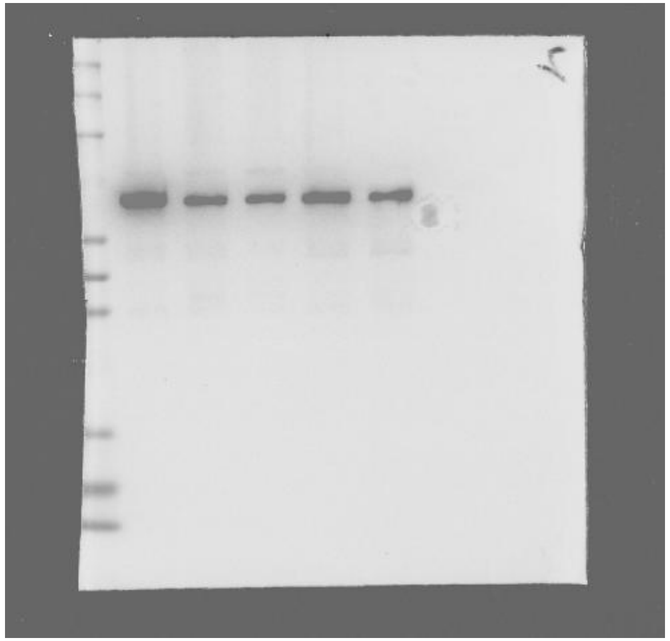

Figure 1c 143B-WT IB:G3BP1

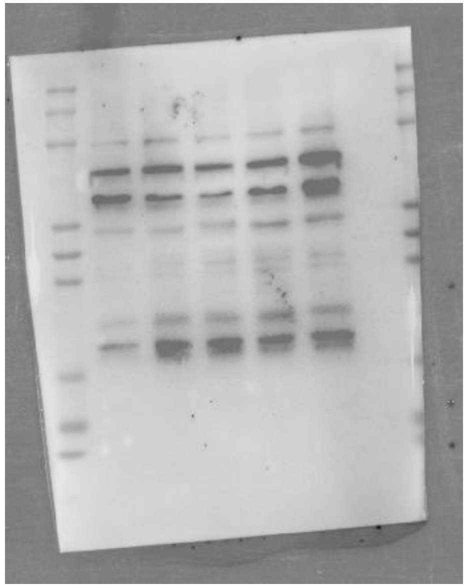

Figure 1c 143B-AEP-KD IB:G3BP1

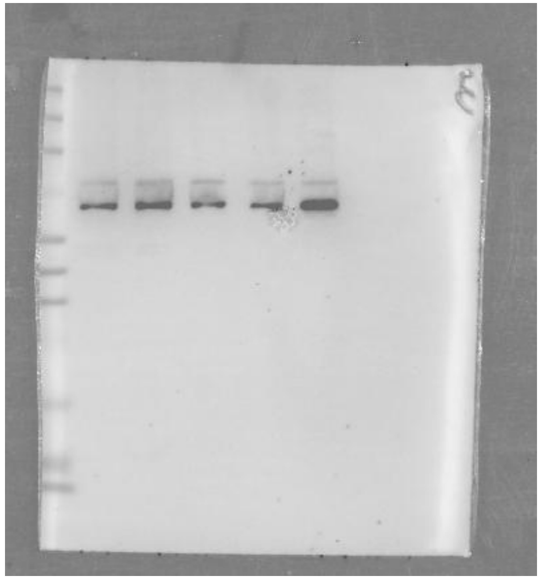

Figure 1c U2OS-wt/AEP-KD IB:AEP

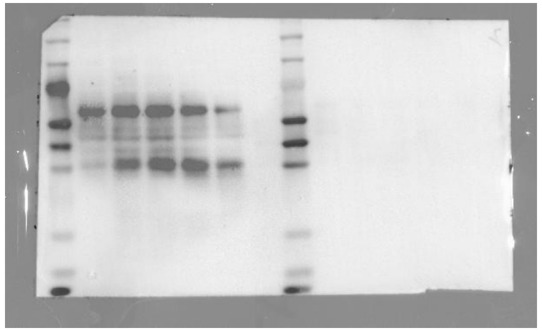

Figure 1c 143B-wt/AEP-KD IB:AEP

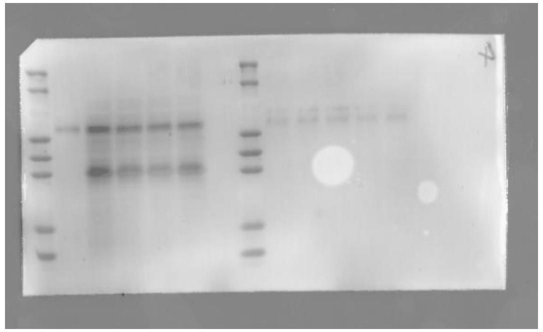

Figure 1c U2OS β-actin

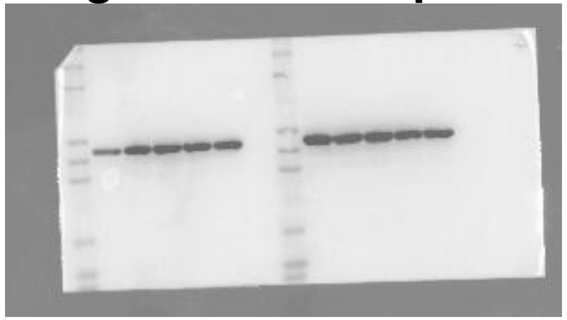

Figure 1c 143B β-actin

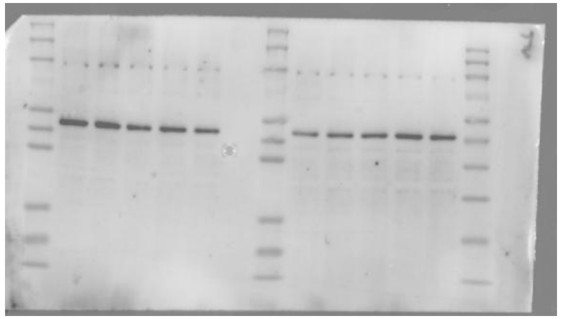

Figure 1d

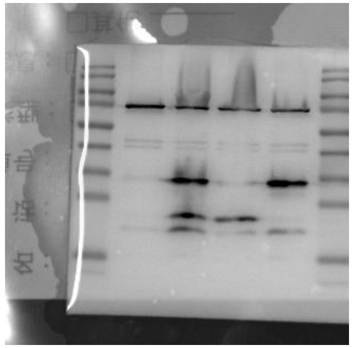

IB:G3BP1

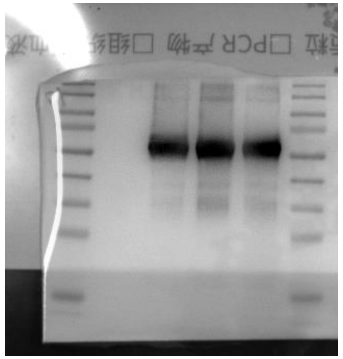

IB:AEP

Figure 2d

Co-IP mCherry

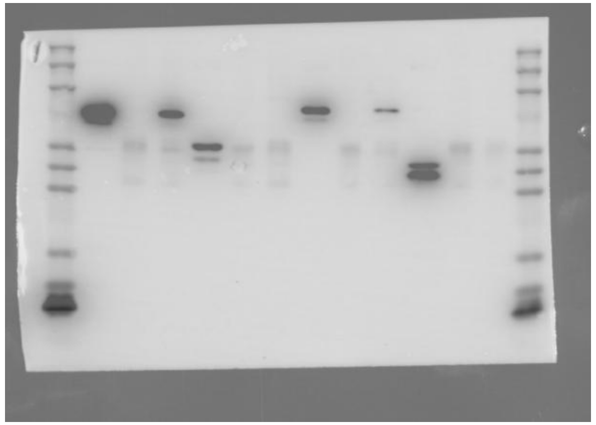

IP Flag

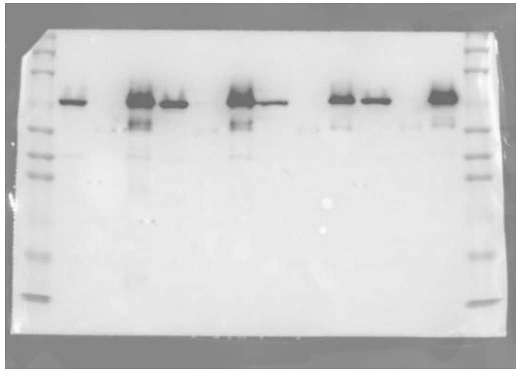

Figure 3c

U2OS 143B U87-MG

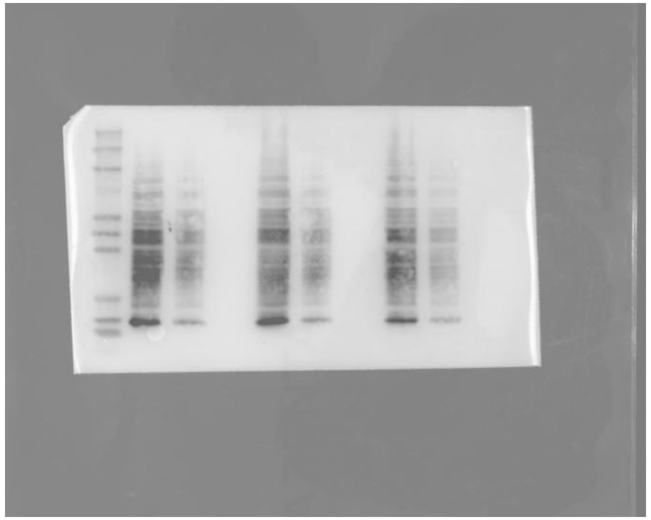

IB: Anti-puromycin

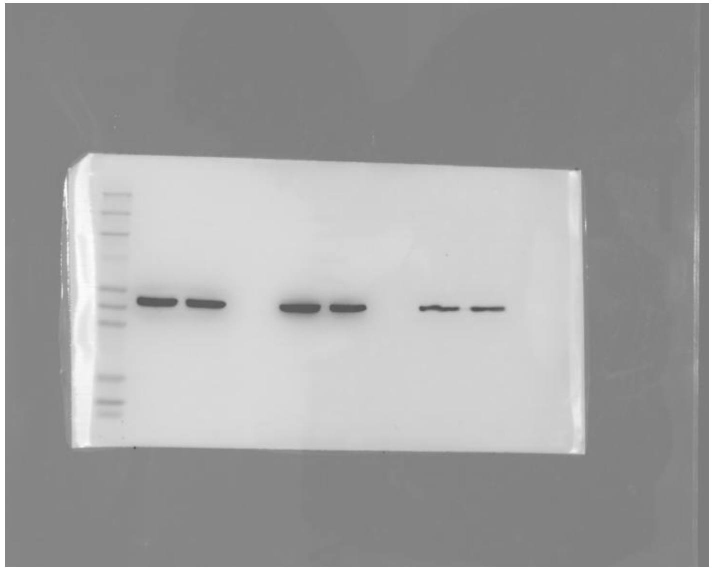

IB: β-actin

Figure 4d U2OS

IB: RPL27A

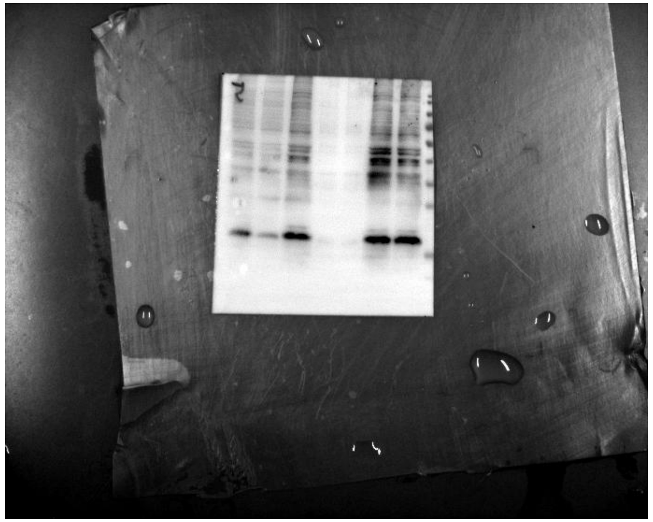

IB: RPL11

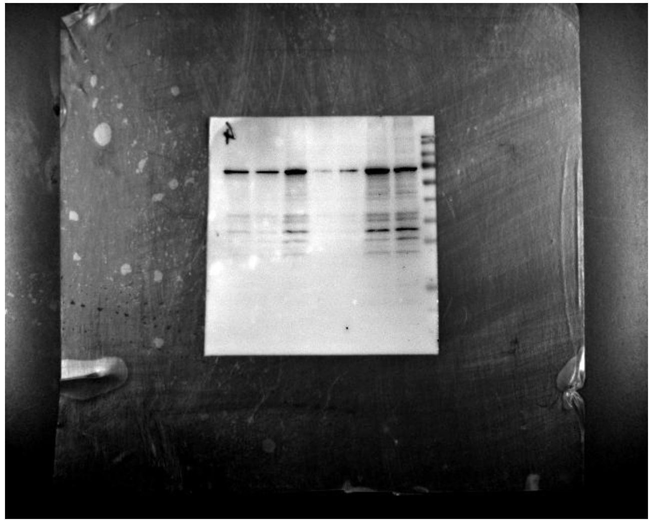

IB: RPS4X

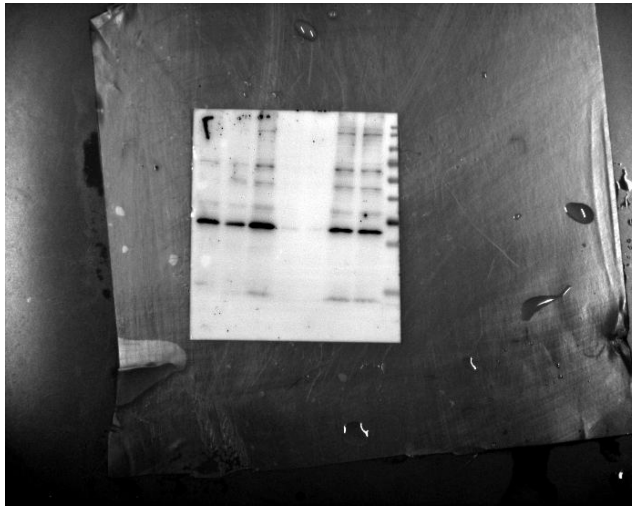

IB: MT-ND1

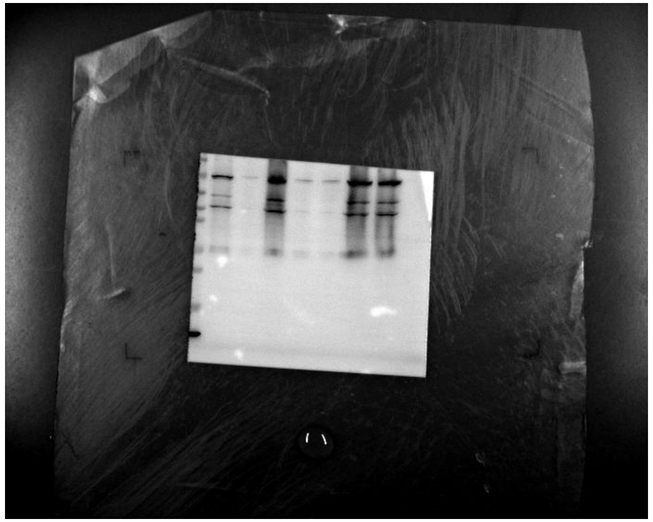

IB: MT-CO1

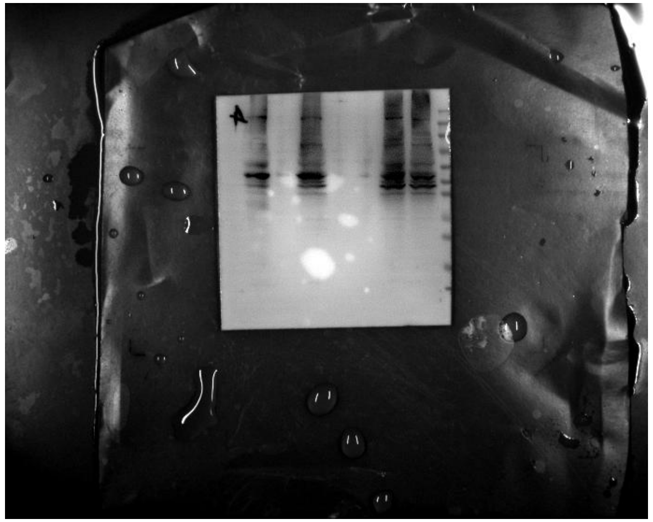

IB: MT-ATP6

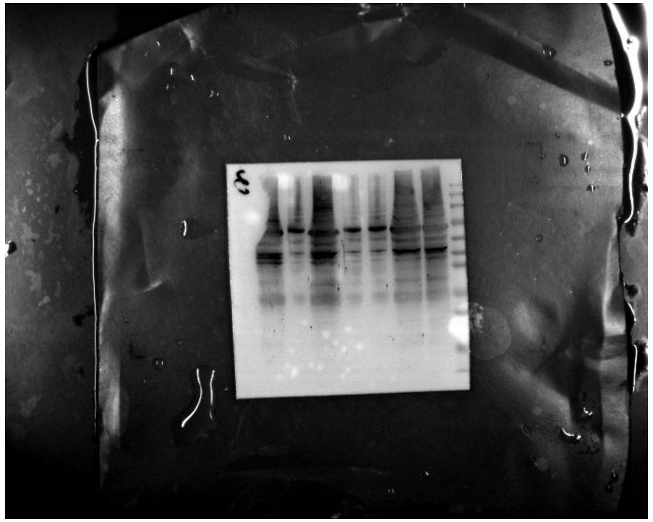

IB: β-actin

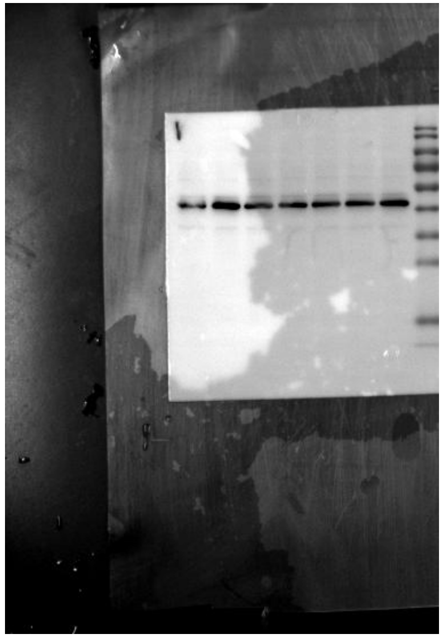

Figure 4d 143B

IB: RPL27A

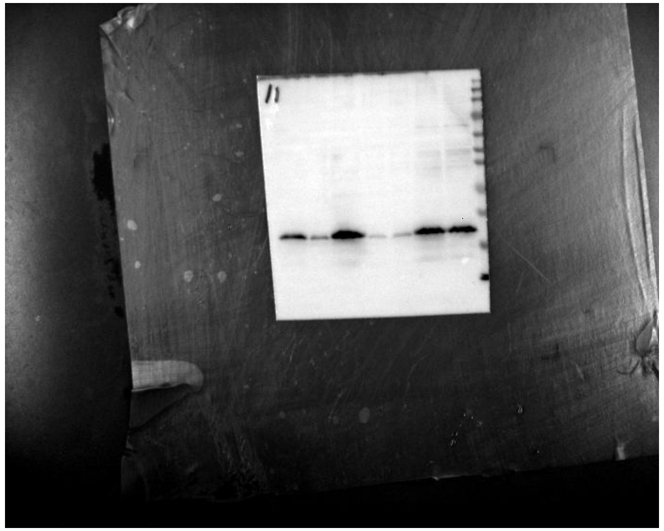

IB: RPL11

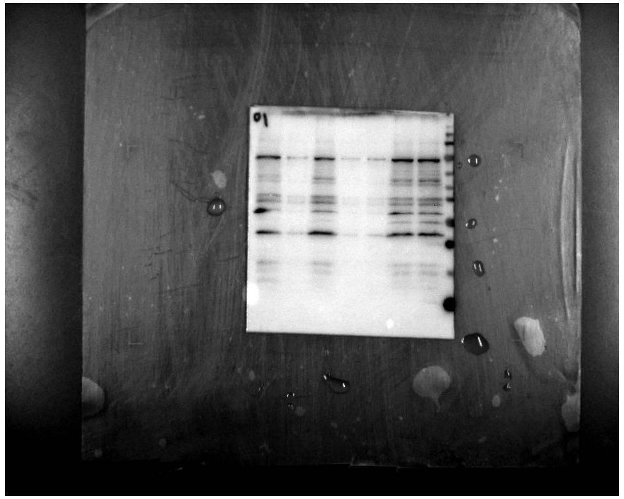

IB: RPS4X

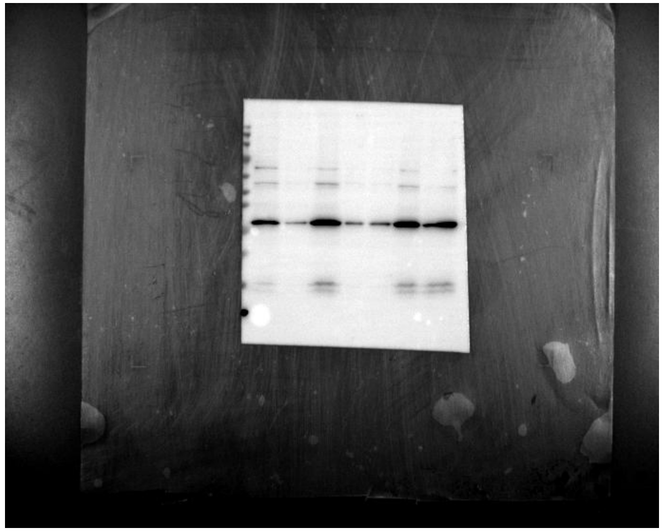

IB: MT-ND1

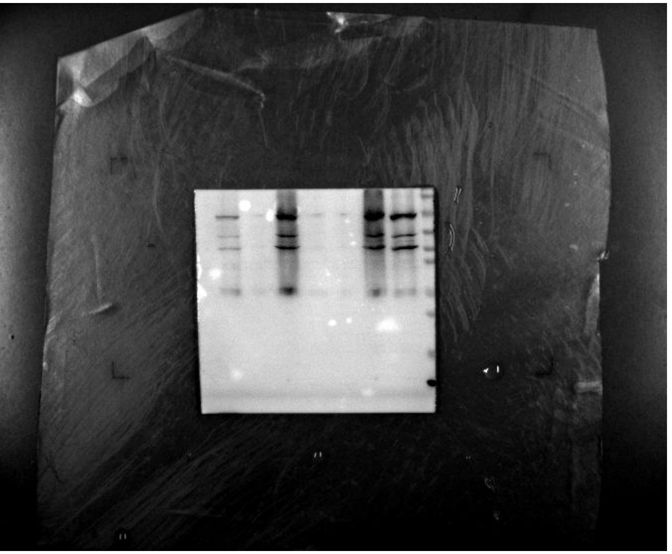

IB: MT-CO1

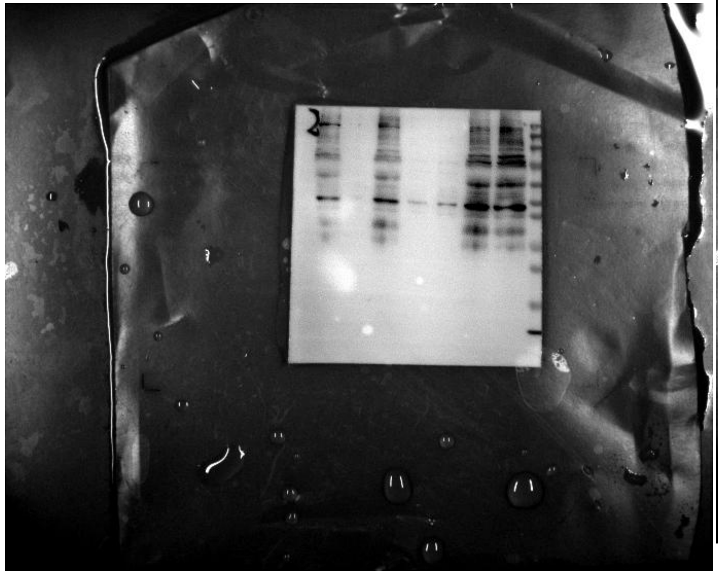

IB: MT-ATP6

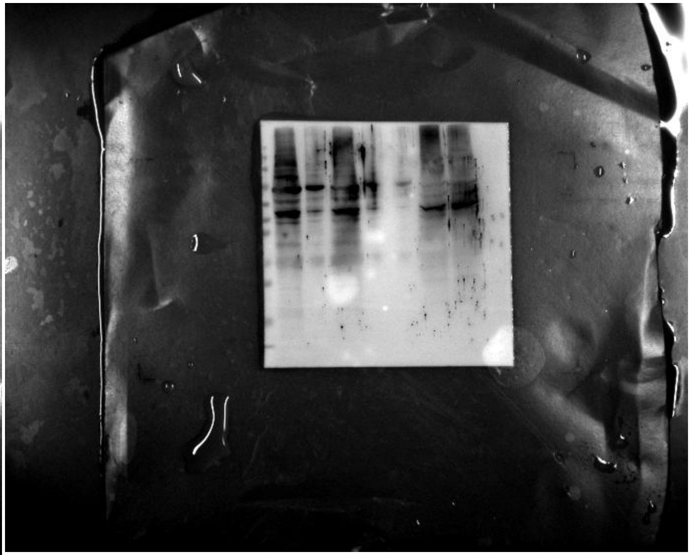

IB: β-actin

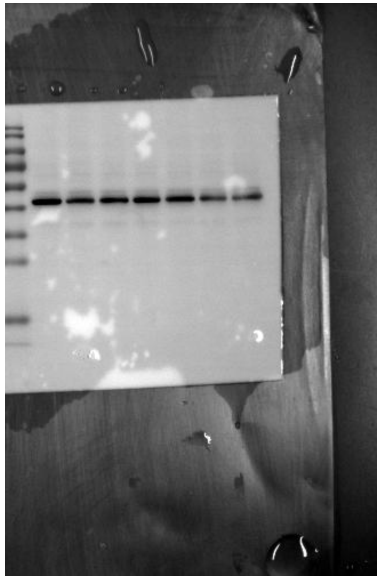

Figure 4d U87-MG

IB: RPL27A

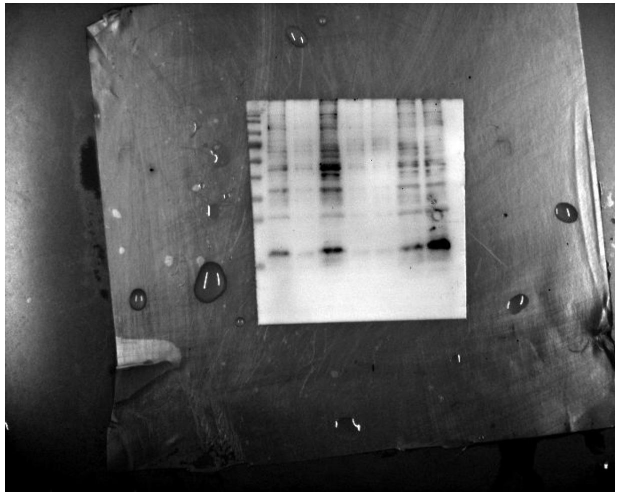

IB: RPL11

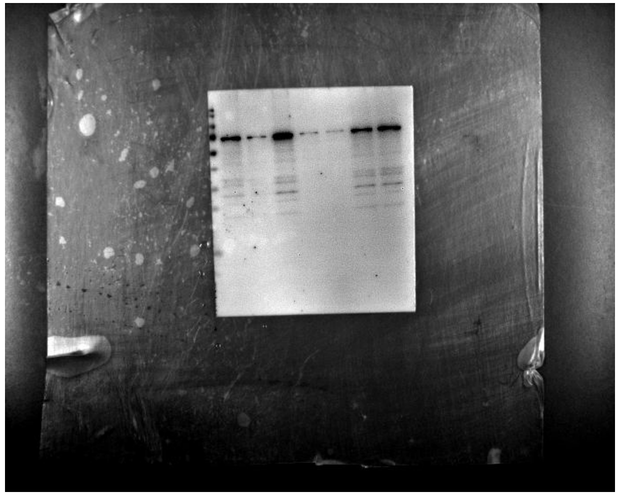

IB: RPS4X

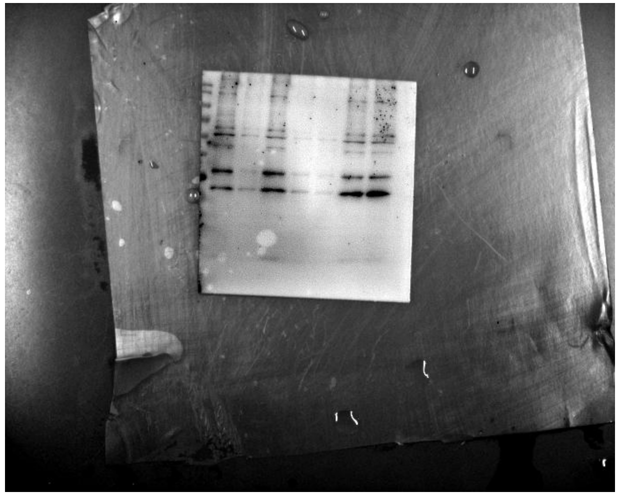

IB: MT-ND1

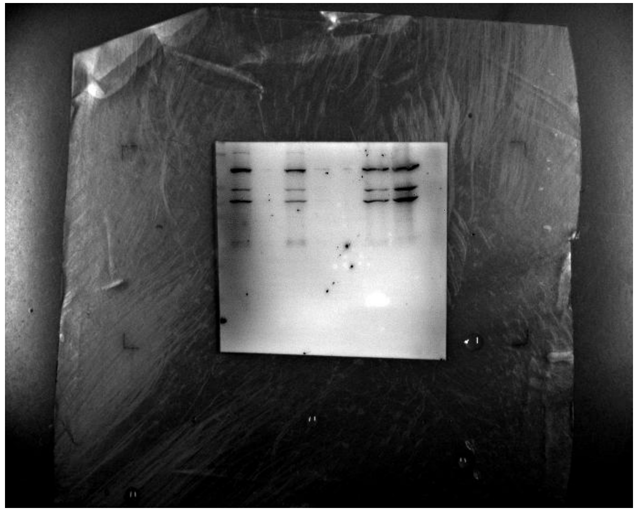

IB: MT-CO1

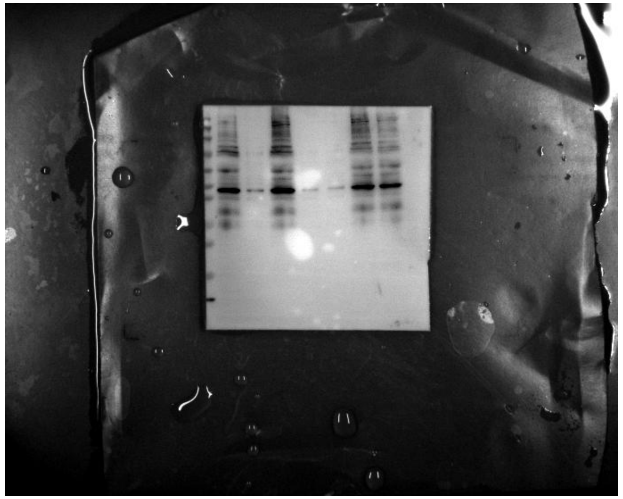

IB: MT-ATP6

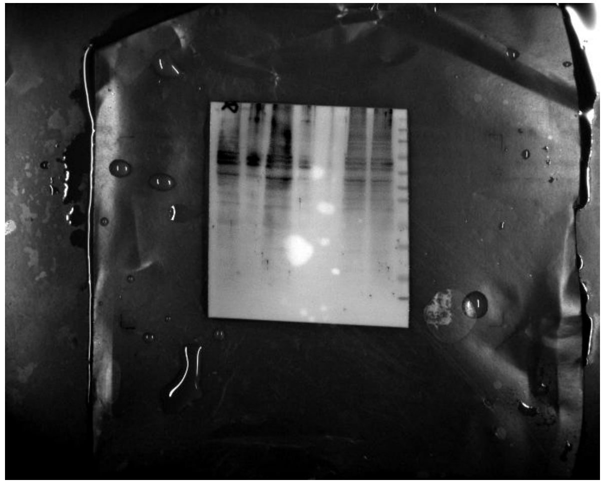

IB: β-actin

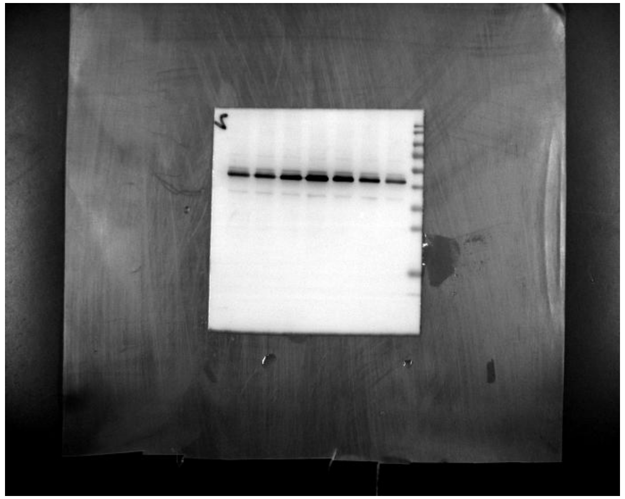

Figure 6a G3BP1

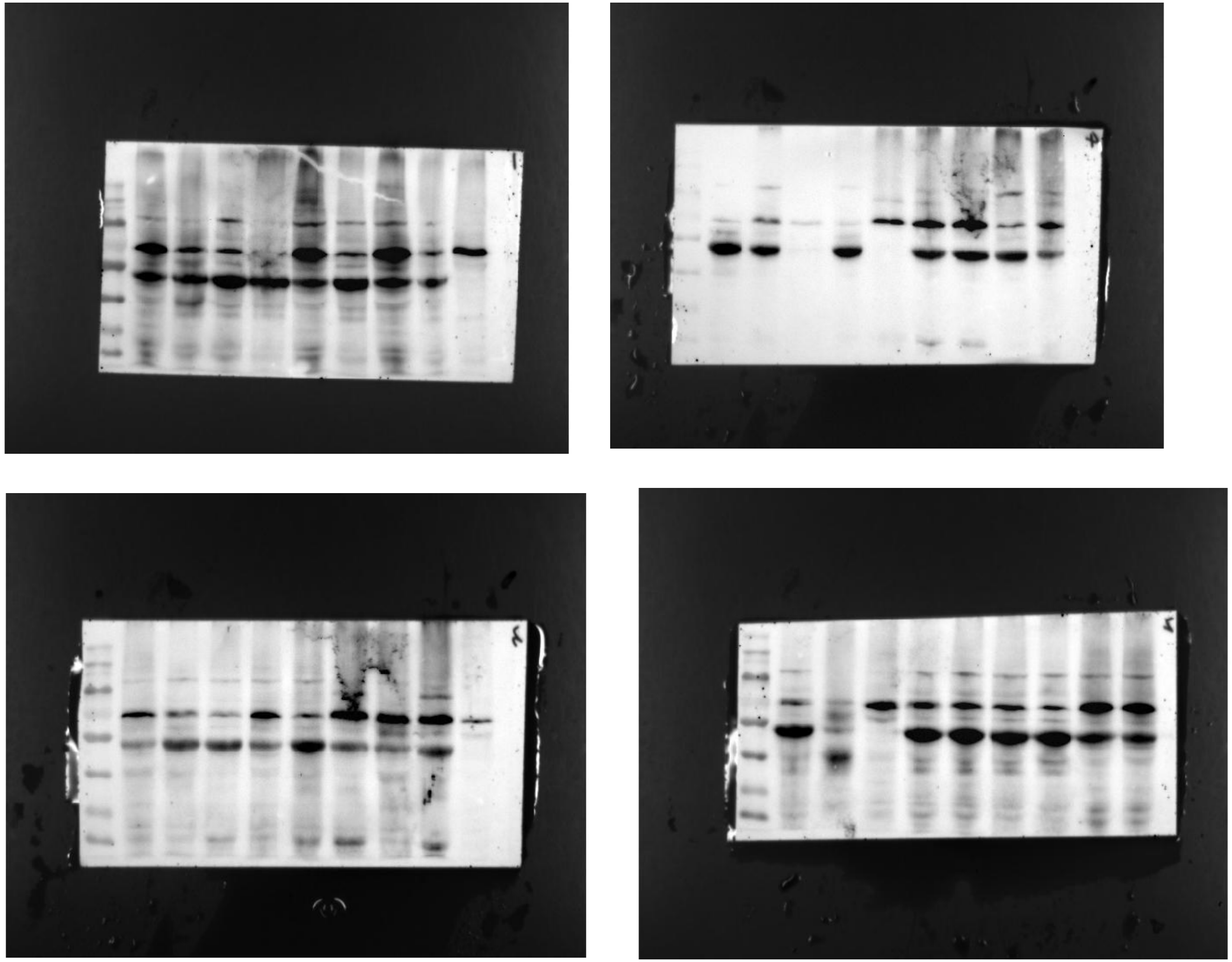

Figure 6a AEP

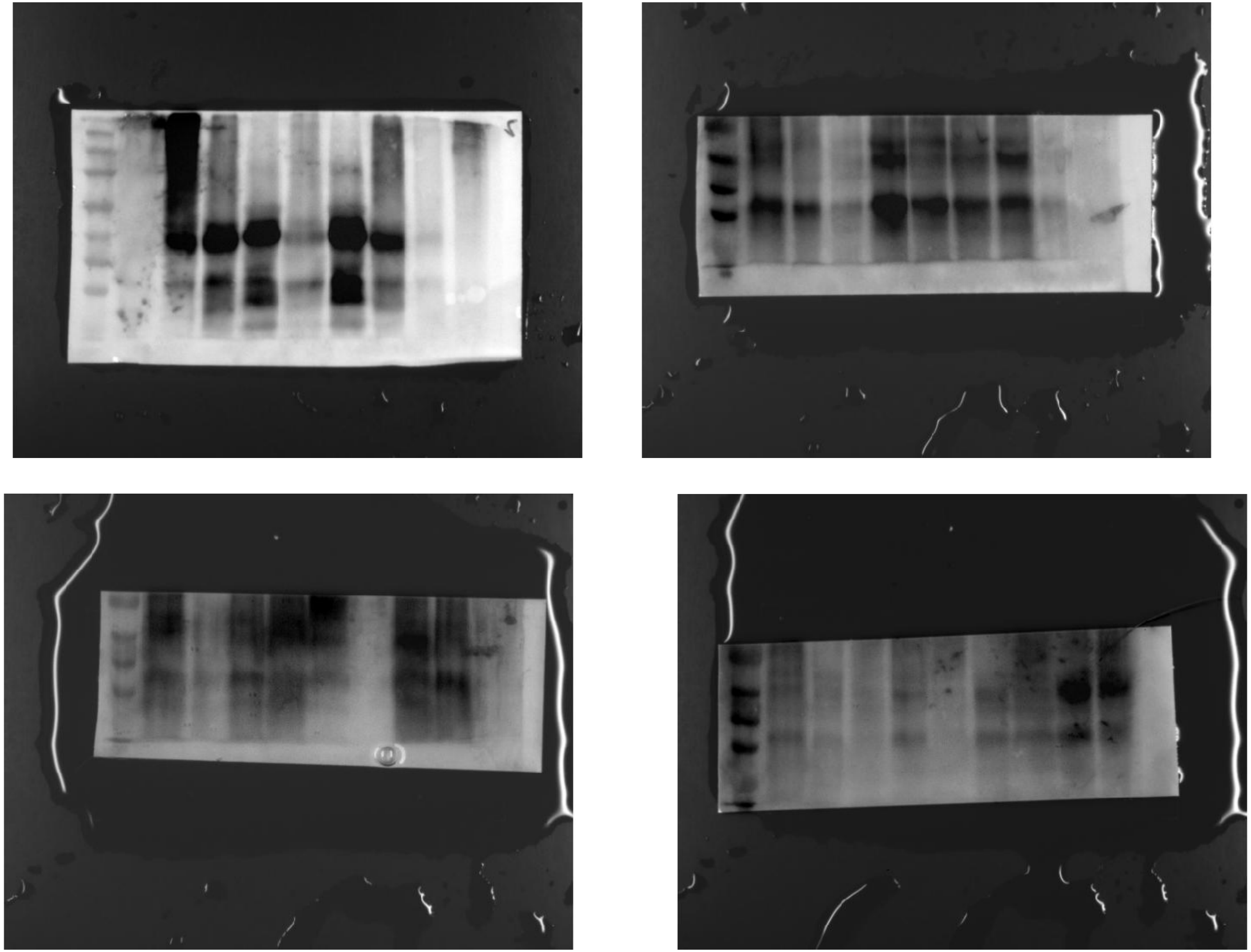

Figure 6a β-actin

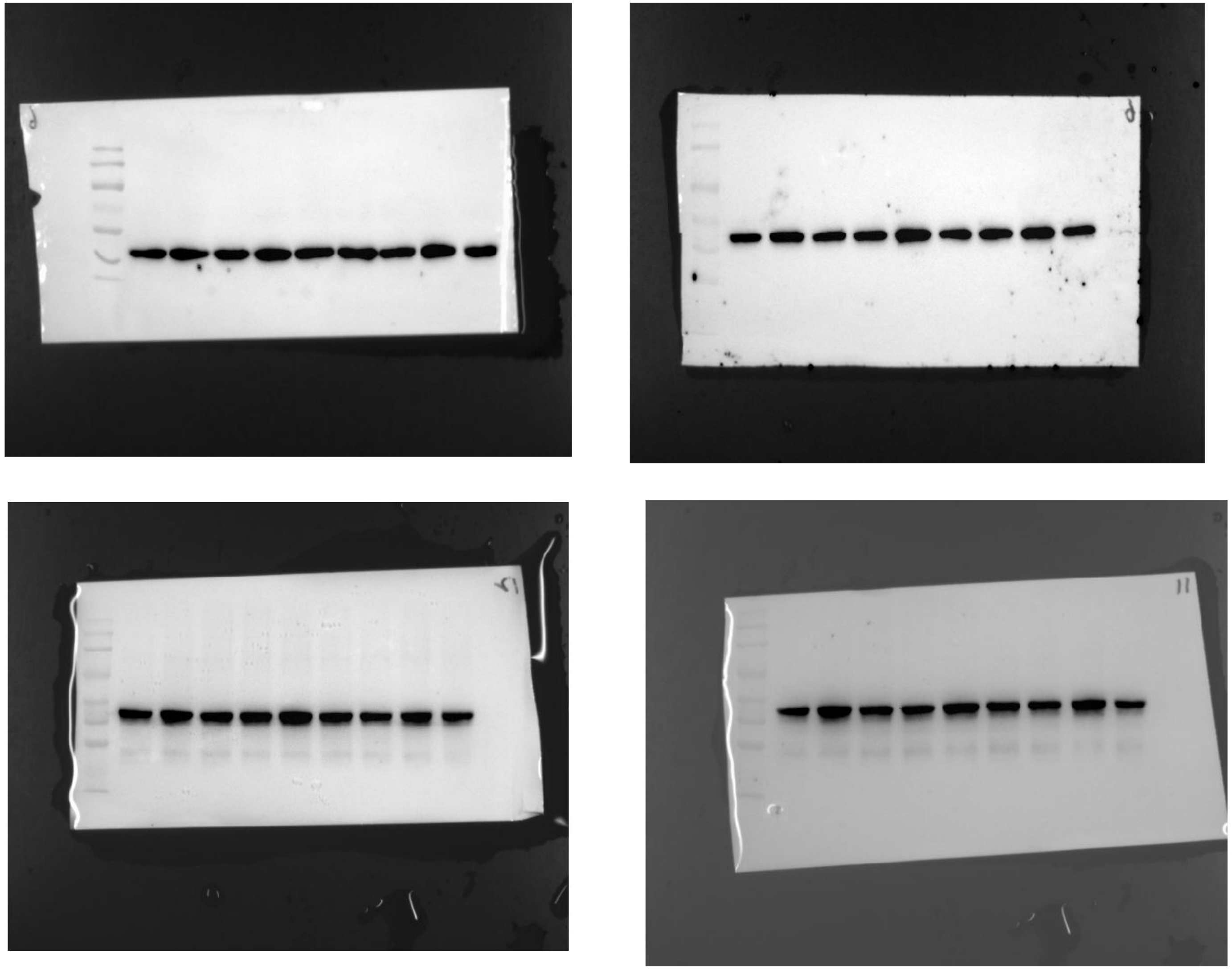

Figure S3a G3BP1

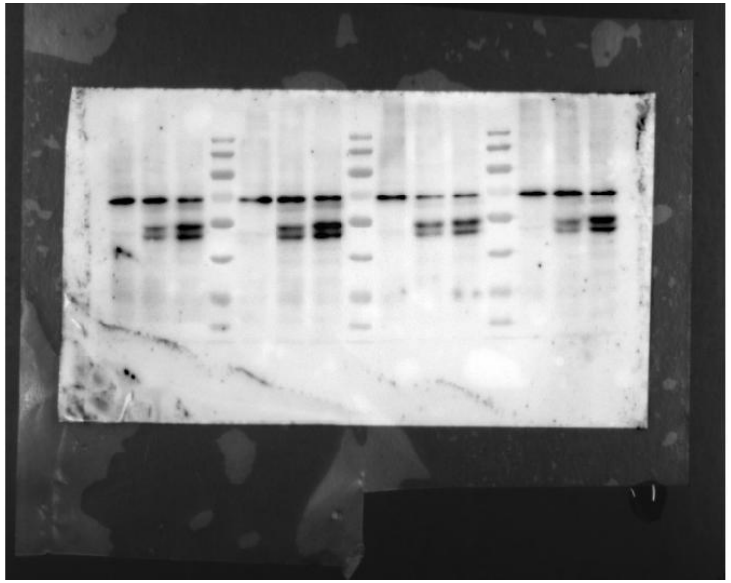

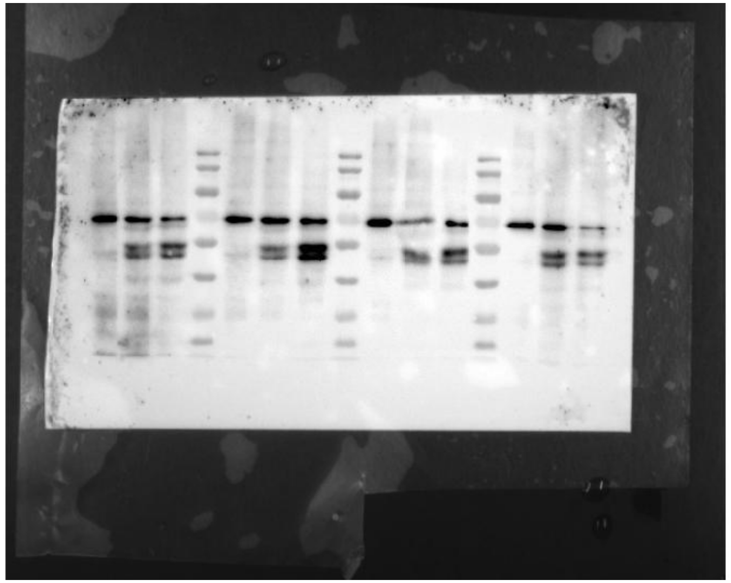

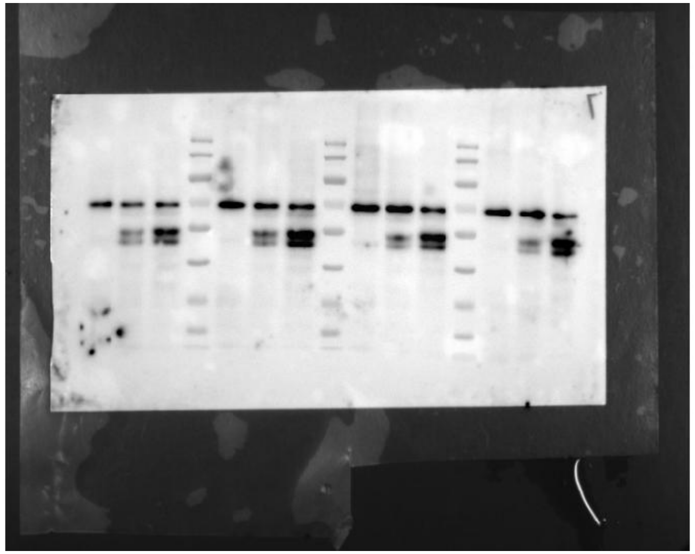

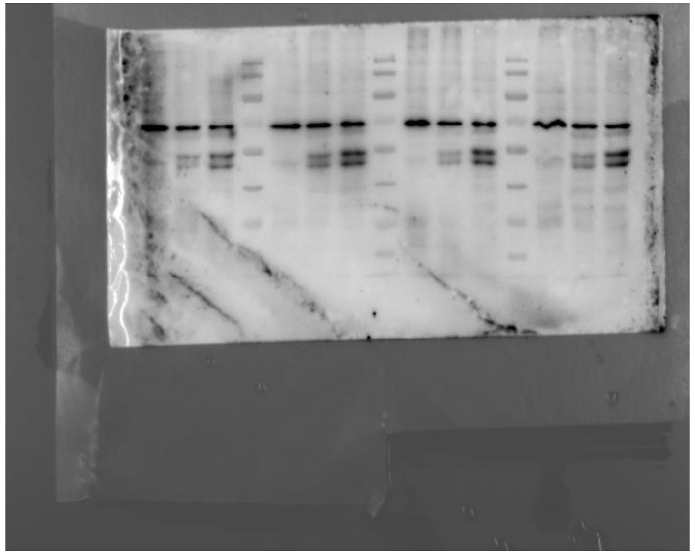

Figure S3a AEP

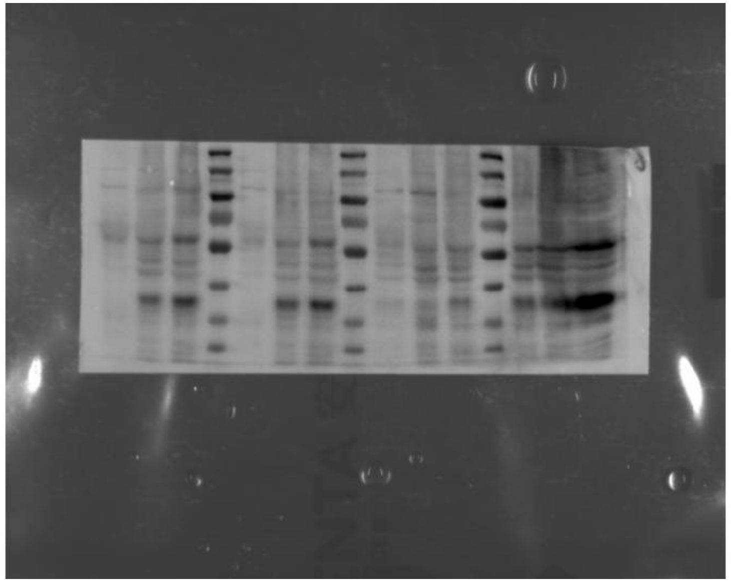

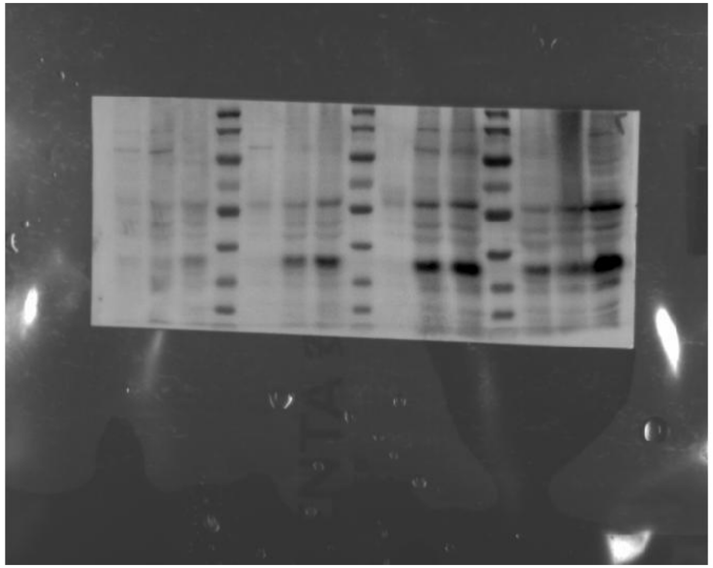

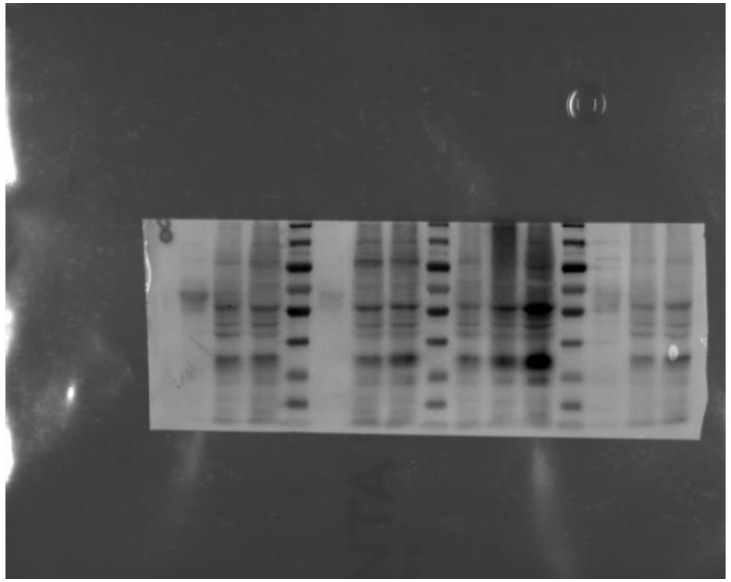

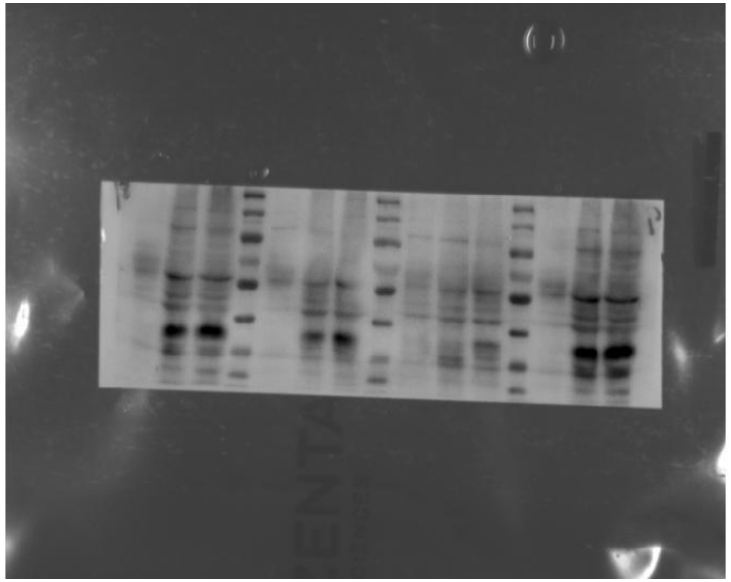

Figure S3a β-actin

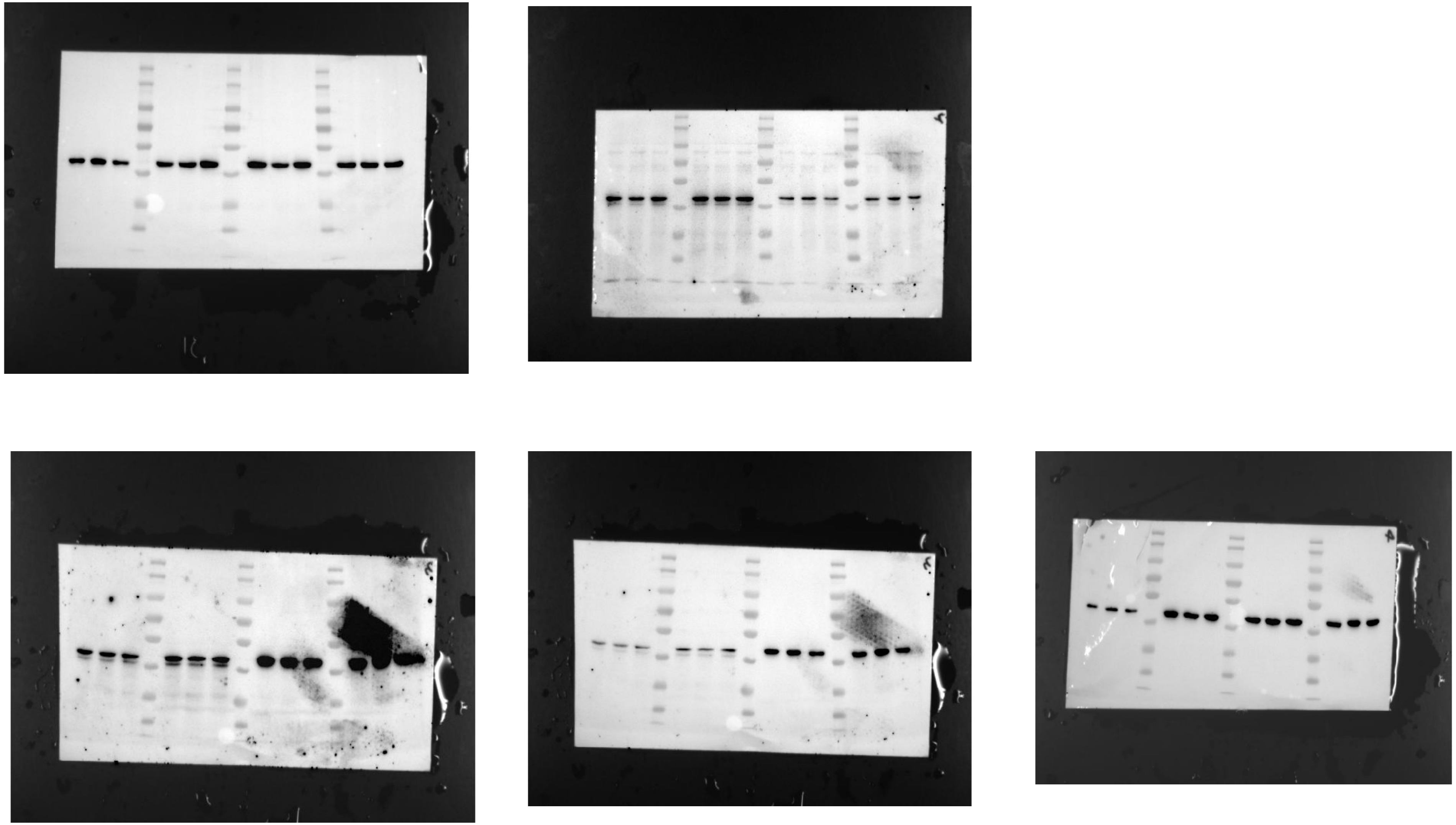

Figure S4a U87-MG-WT IB: G3BP1

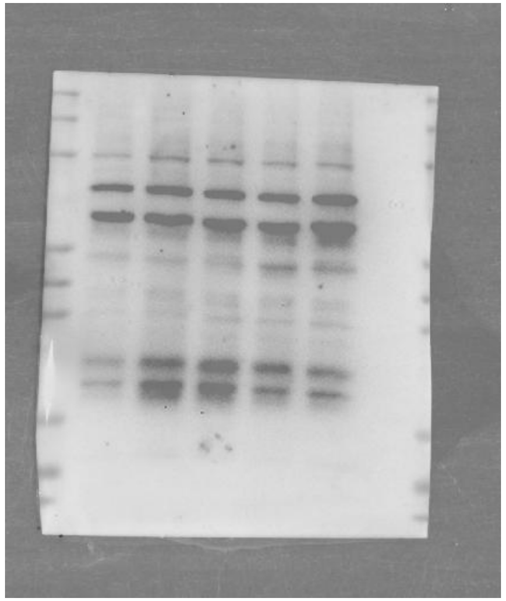

U87-wt/AEP-KD IB:AEP

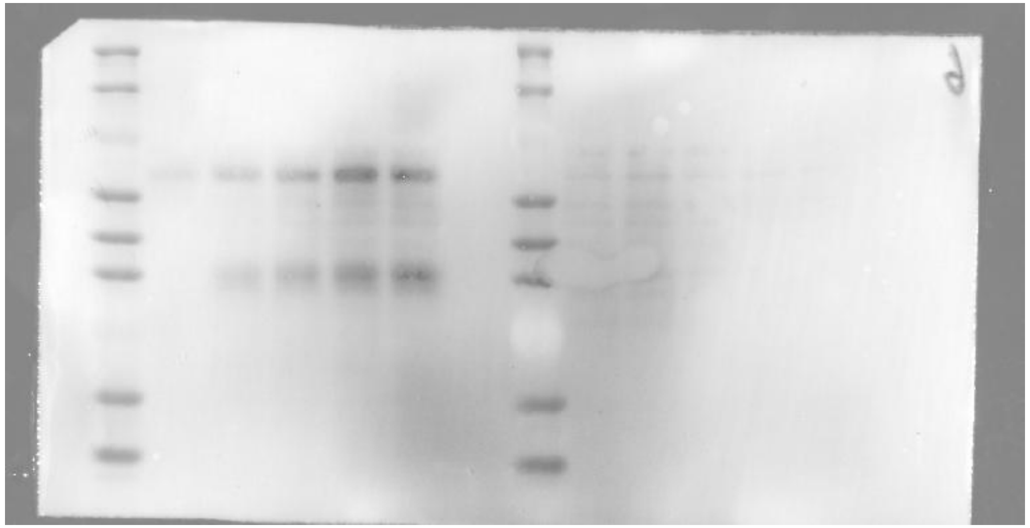

U87-MG-AEP-KD IB:G3BP1

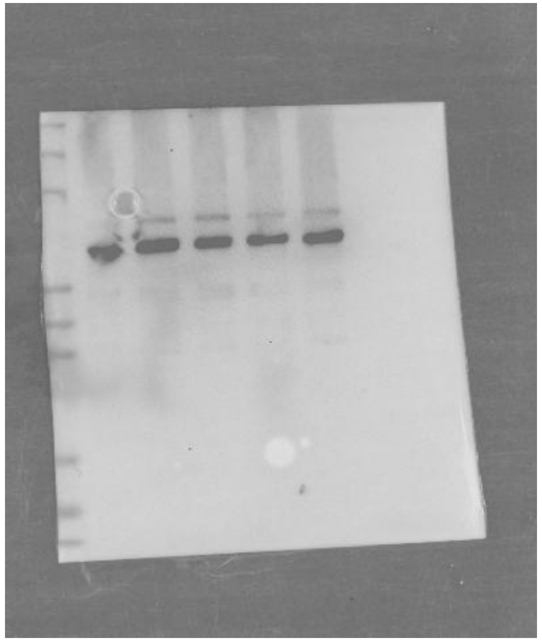

U87 β-actin

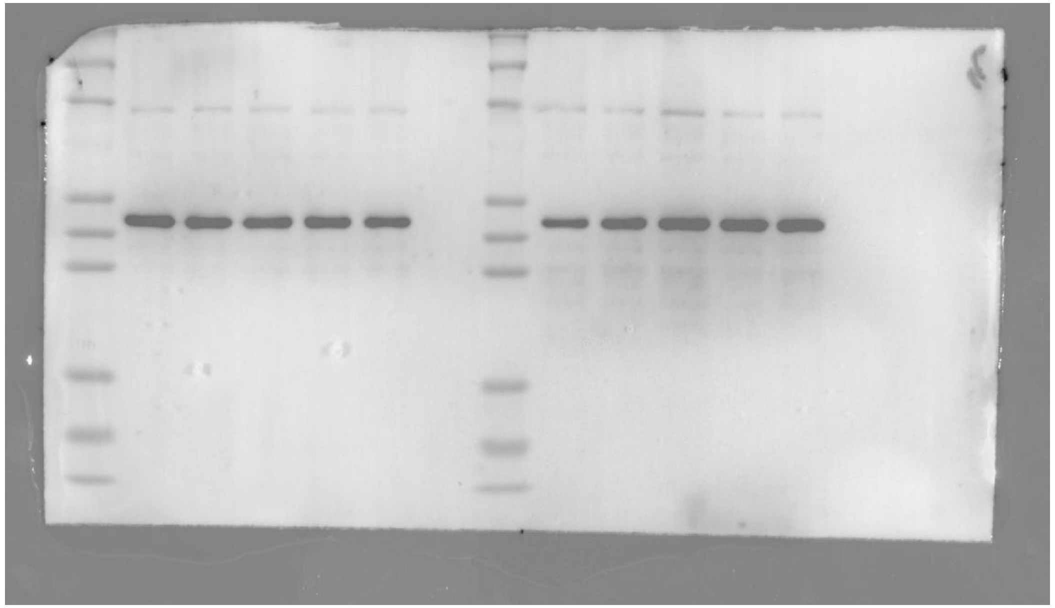

Figure S4b

IB:AEP

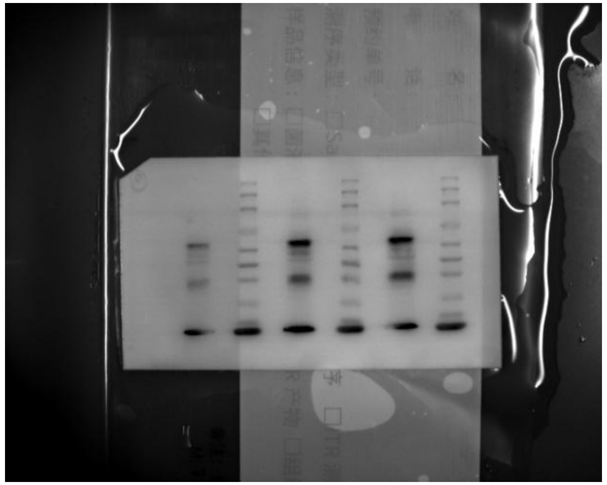

IB:β-actin

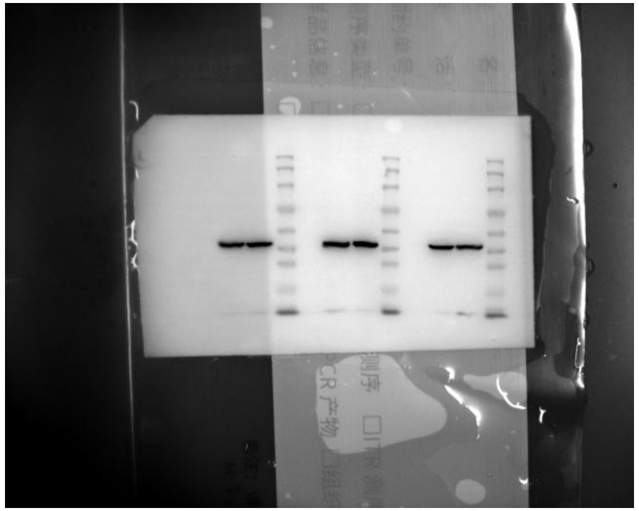

Figure S4c IB:G3BP1

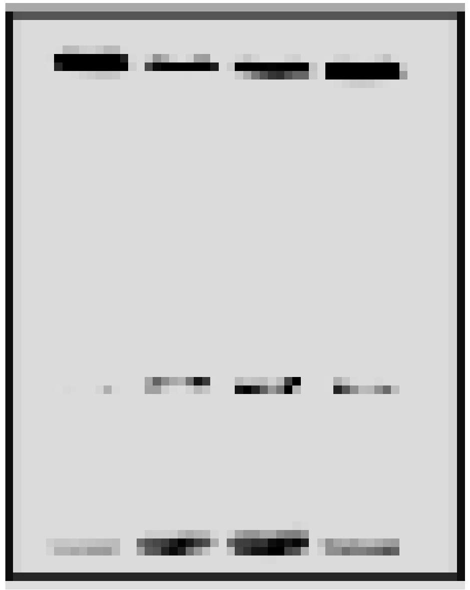

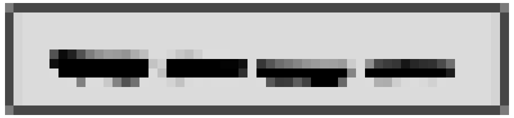

Figure S4d IB:G3BP1

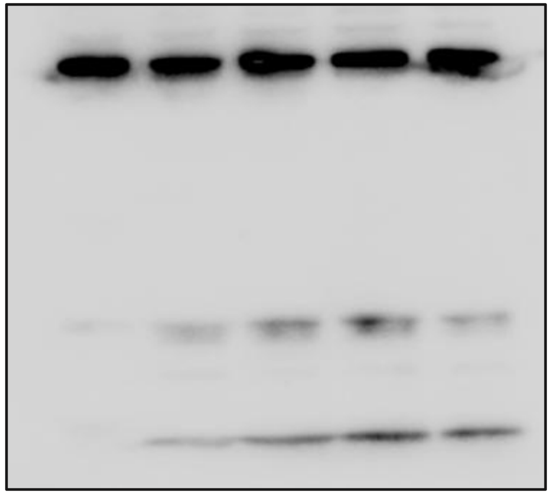

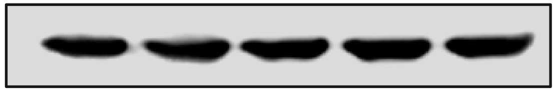

Figure S4e

Sliver Stain

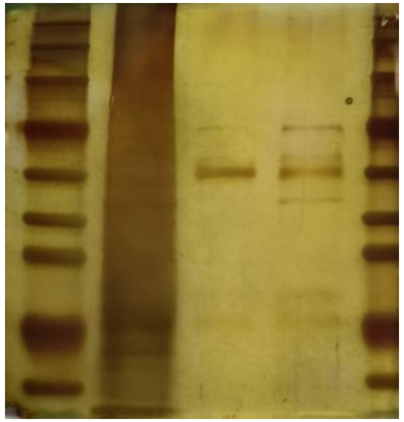

Figure S4f
Co-IP G3BP1

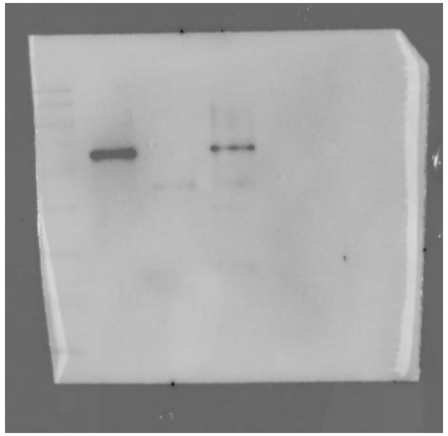

IP AEP

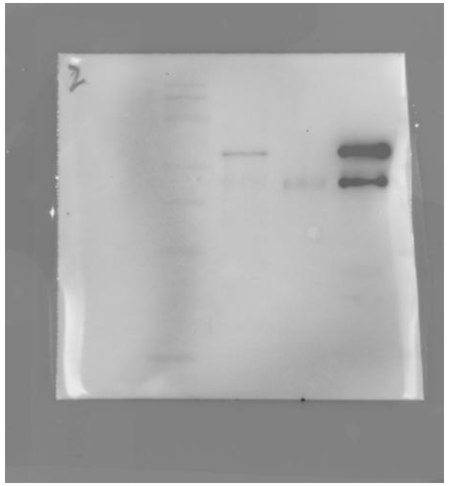

Figure S4h

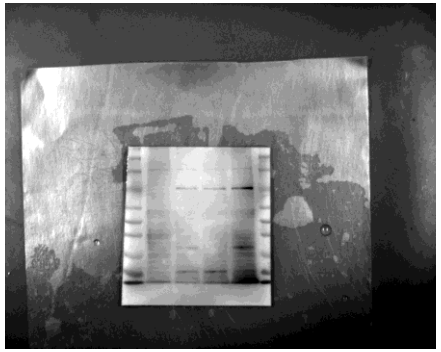

IB:G3BP1

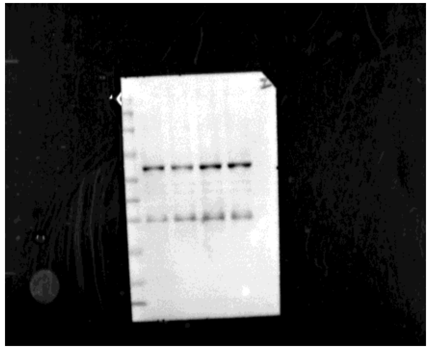

IB: AEP

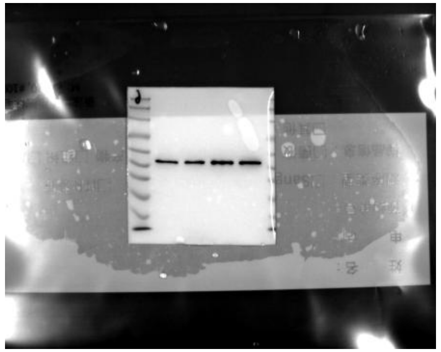

IB: β-actin

Figure S4i IB:G3BP1

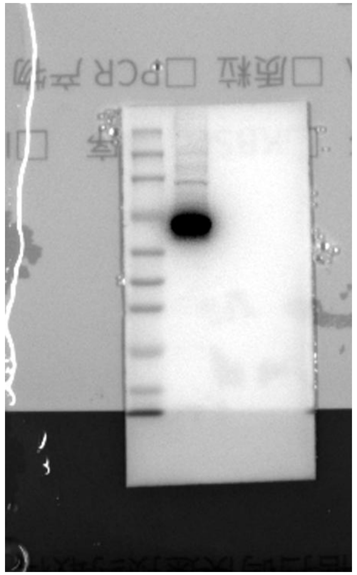

IB: G3BP2

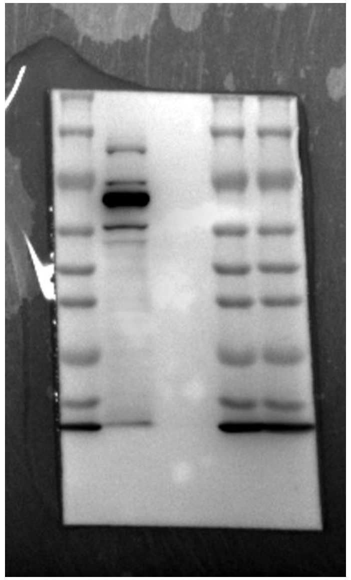

IB: β-actin

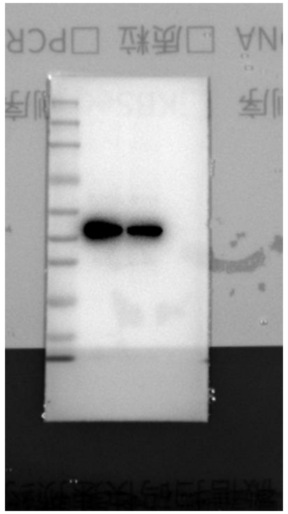

Figure S5a

U87-MG IB:p53

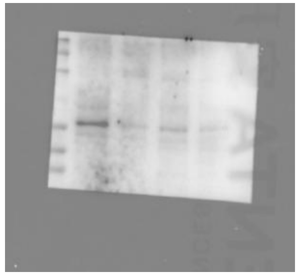

143B IB:p53

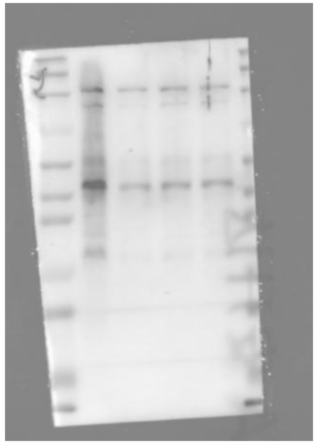

U2OS IB:p53

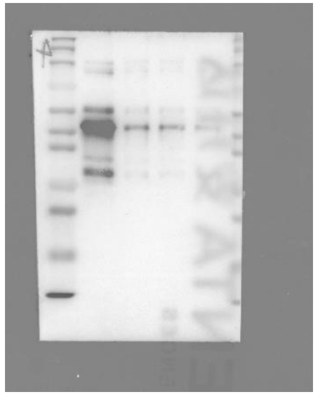

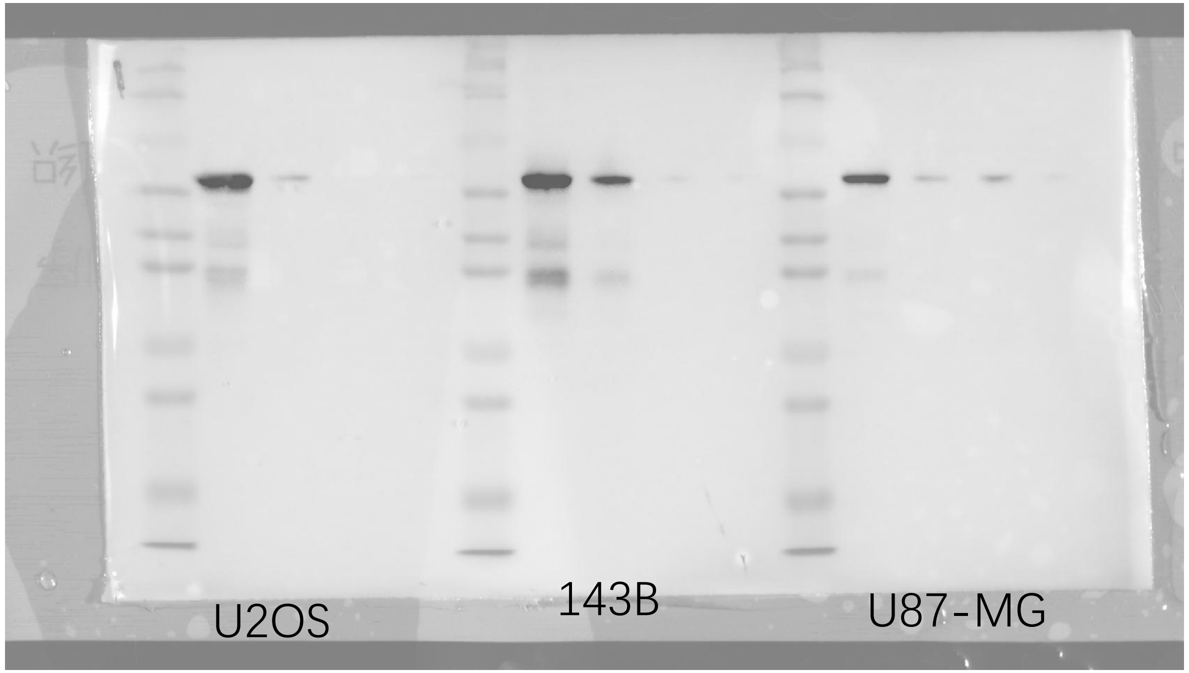

IB:AEP

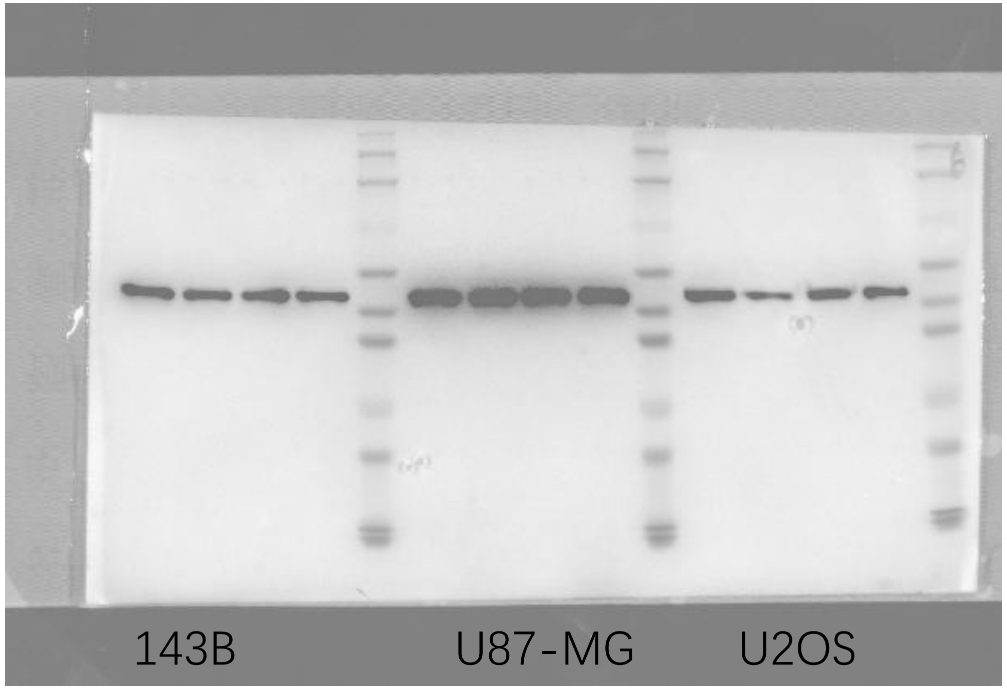

IB:β-actin

Figure S5b

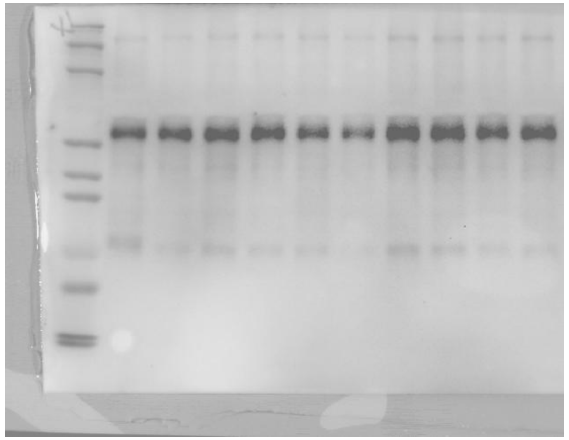

IB:AEP

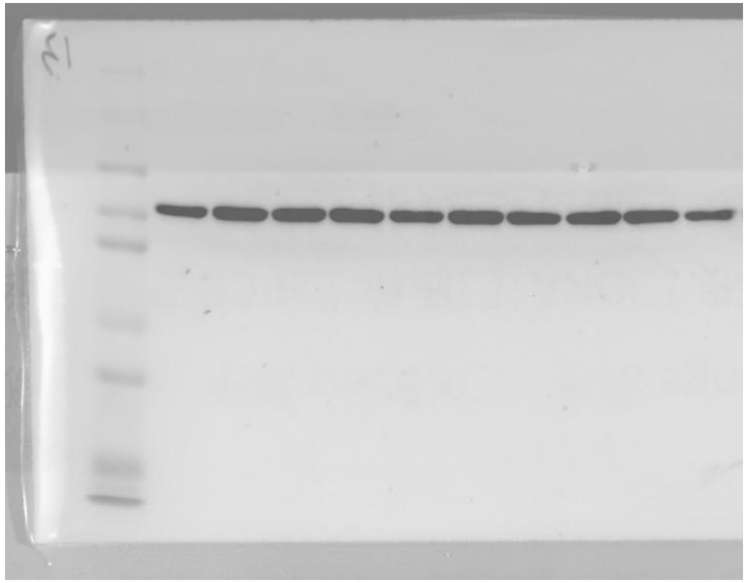

IB:β-actin

Figure S5c

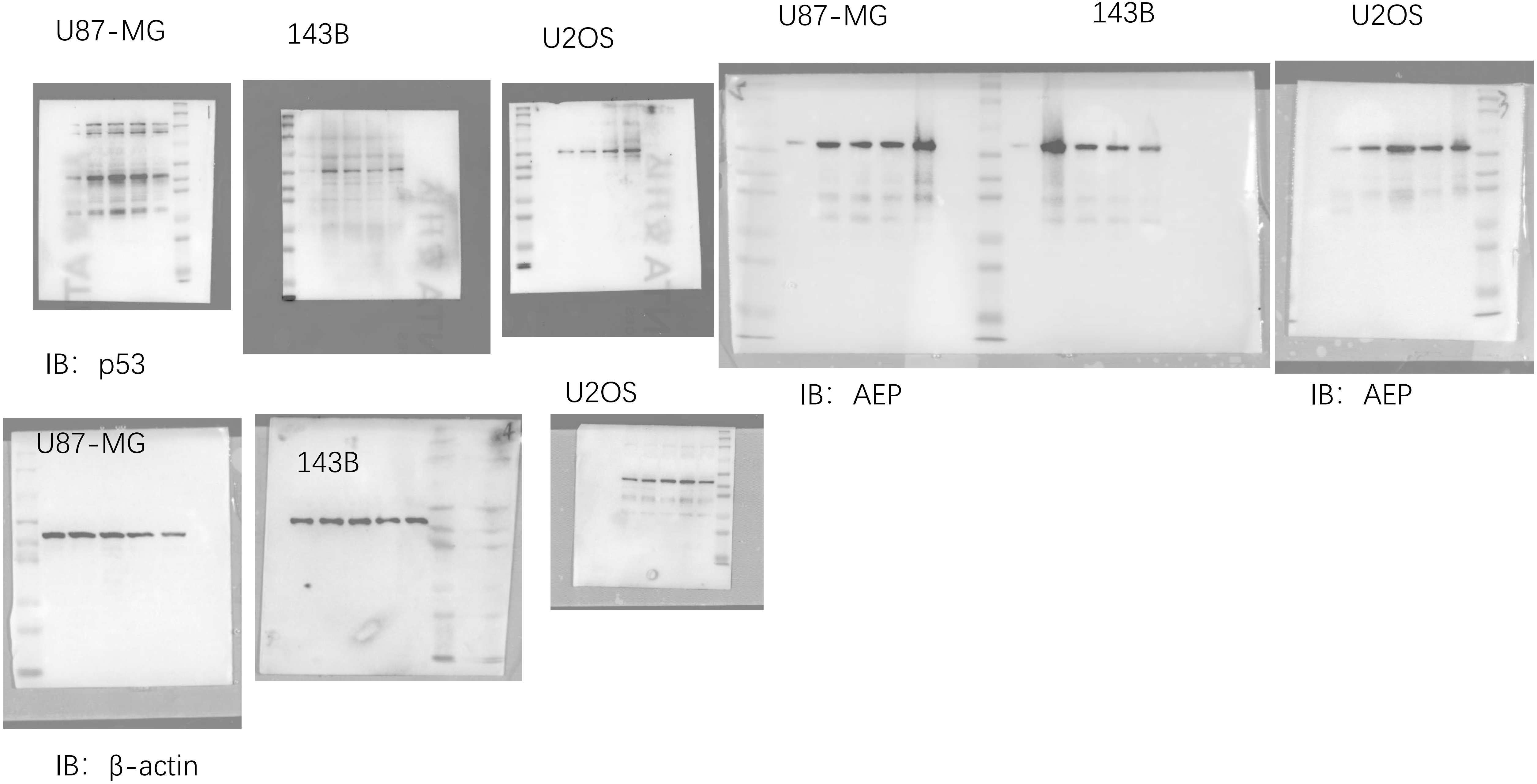
U87-MG
143B
U2OS
U87-MG
143B
U2OS
IB： p53
IB： AEP
IB： AEP
U2OS
U87-MG
143B
IB： β-actin

Figure S5c A549

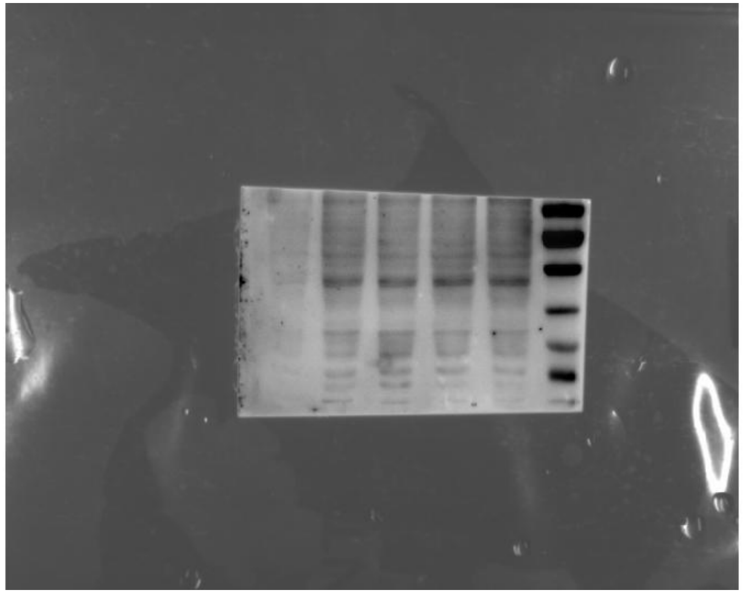

IB: p53

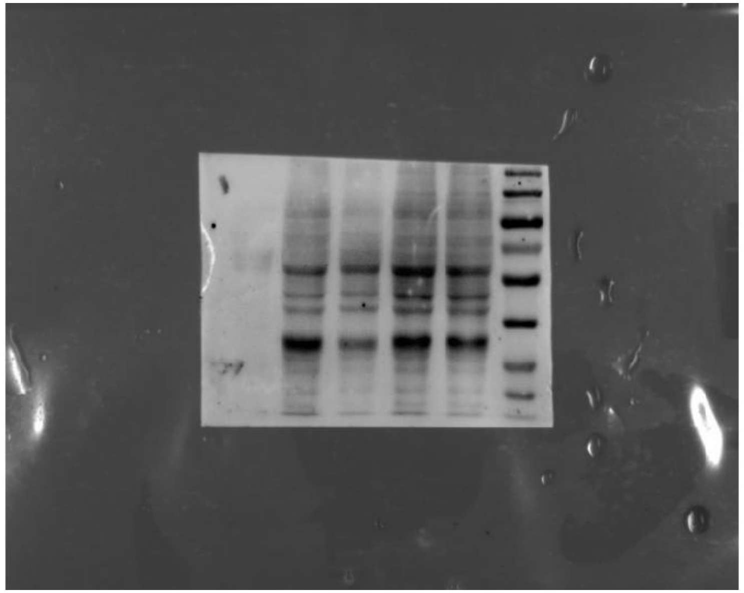

IB: AEP

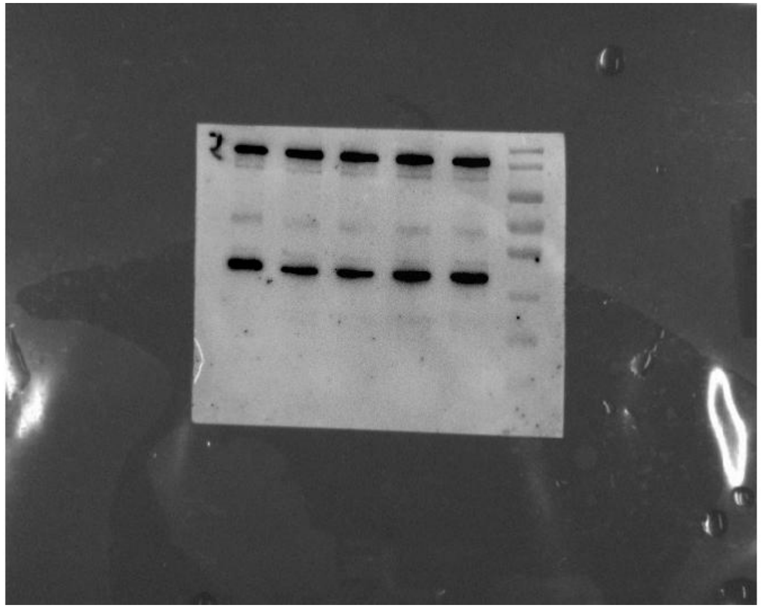

IB: β-actin

Figure S7c IB: Flag

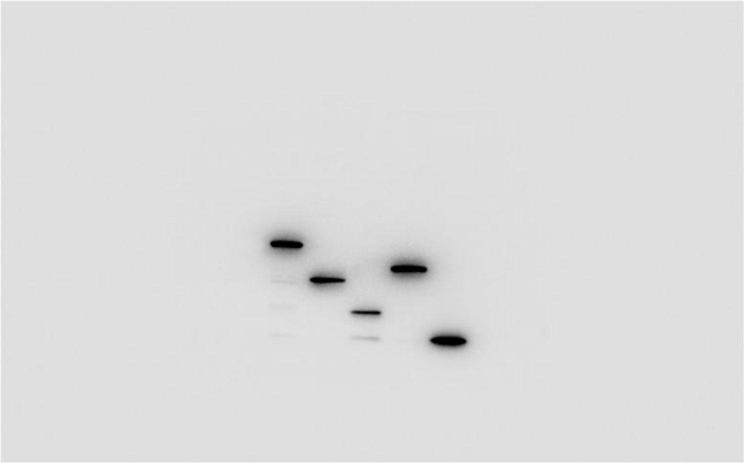

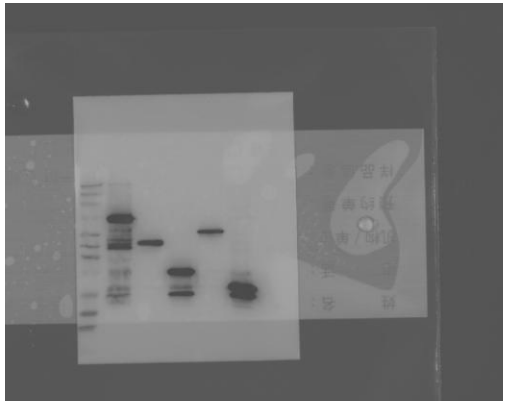

Figure S8e IB Flag

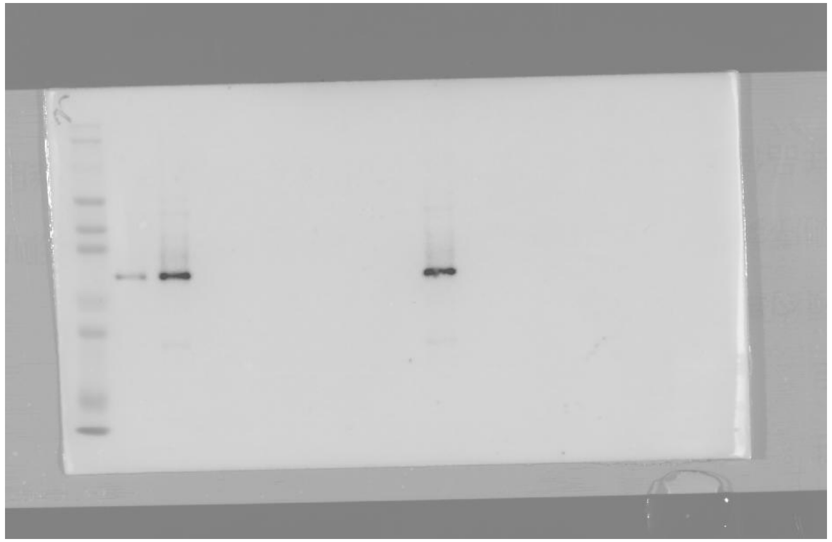

tG3BP1-C1-Flag

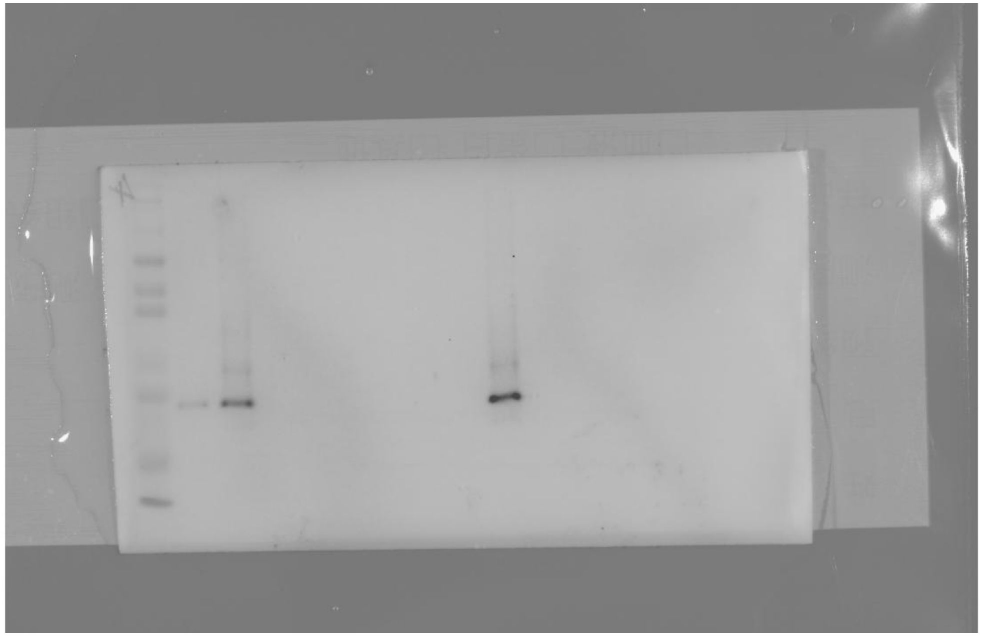

tG3BP1-C2-Flag

Figure S14a IB： G3BP1

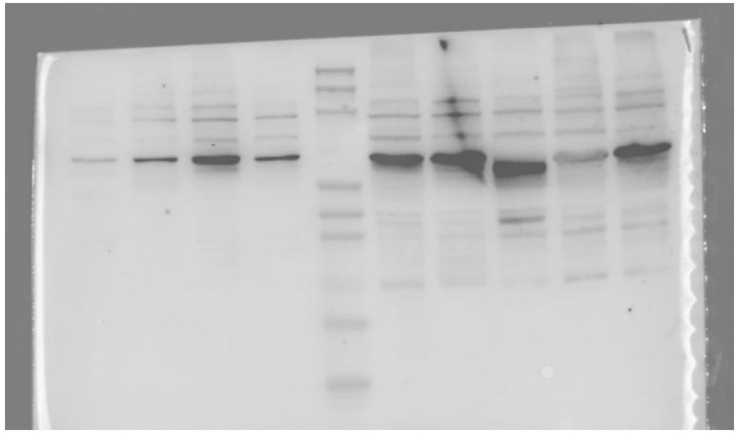
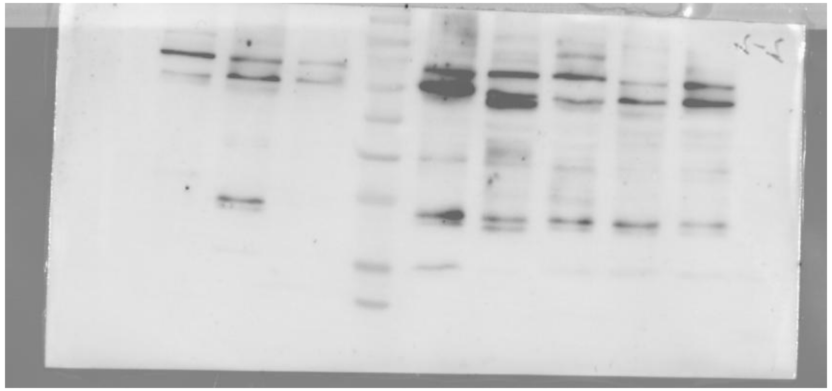
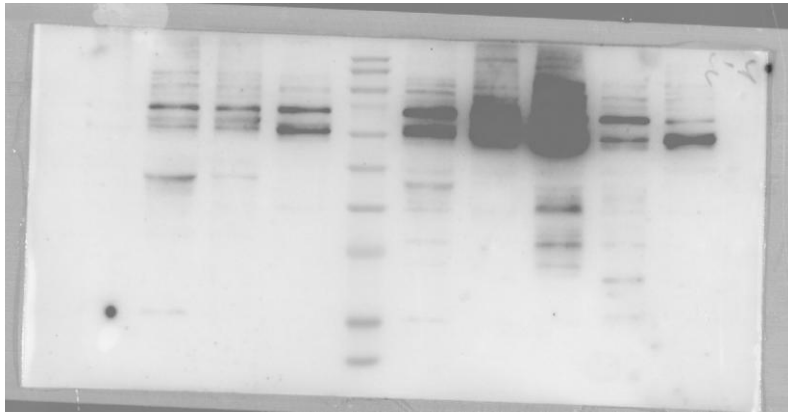
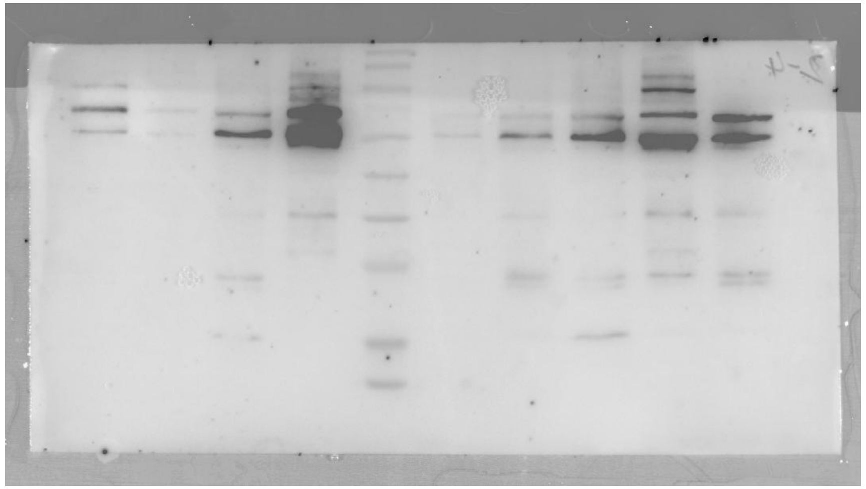
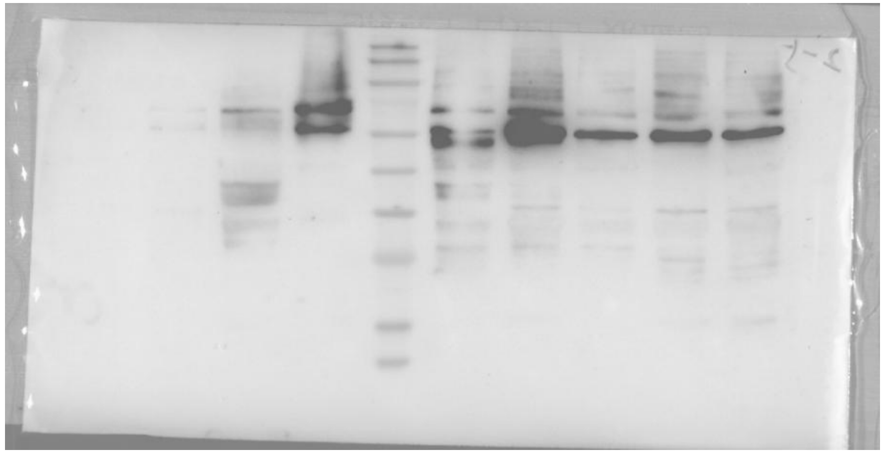

Figure S14a IB：AEP

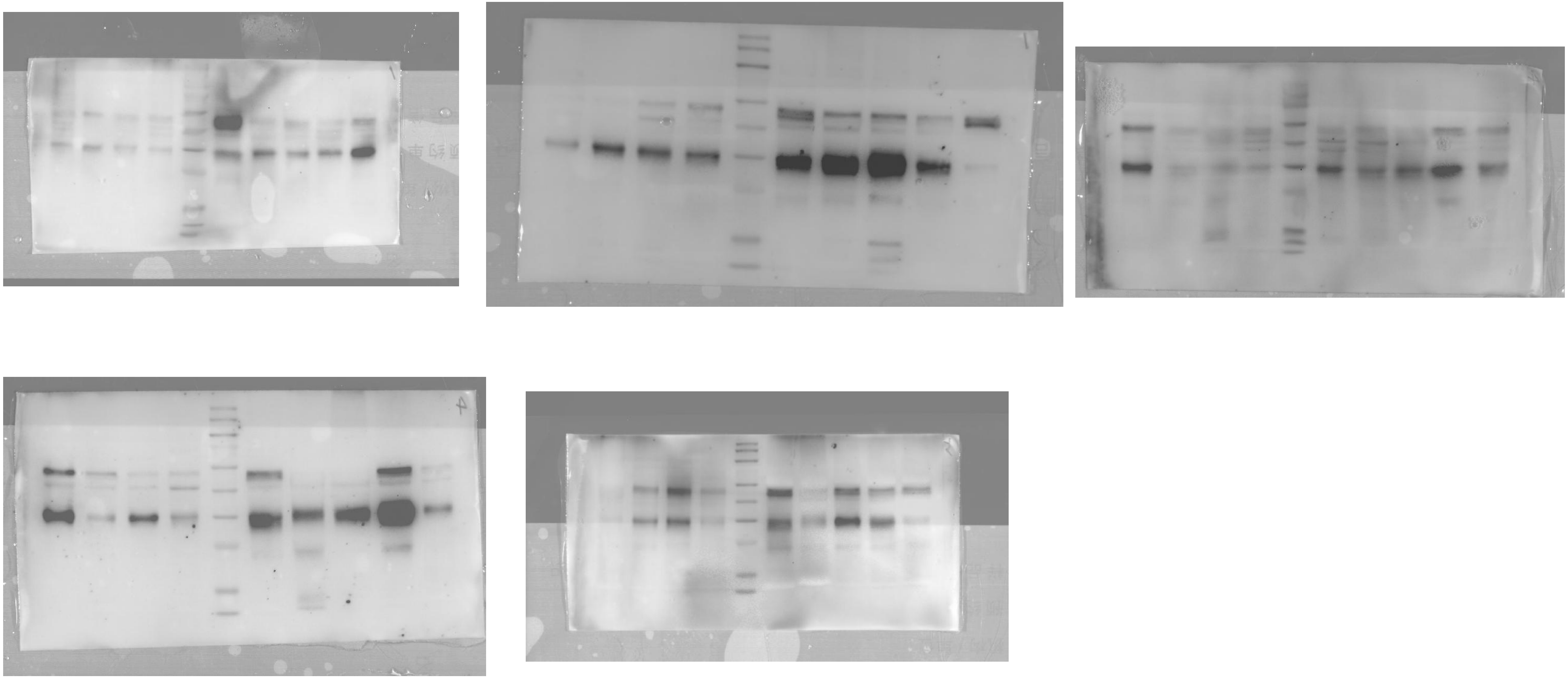

Figure S14a IB： β-actin

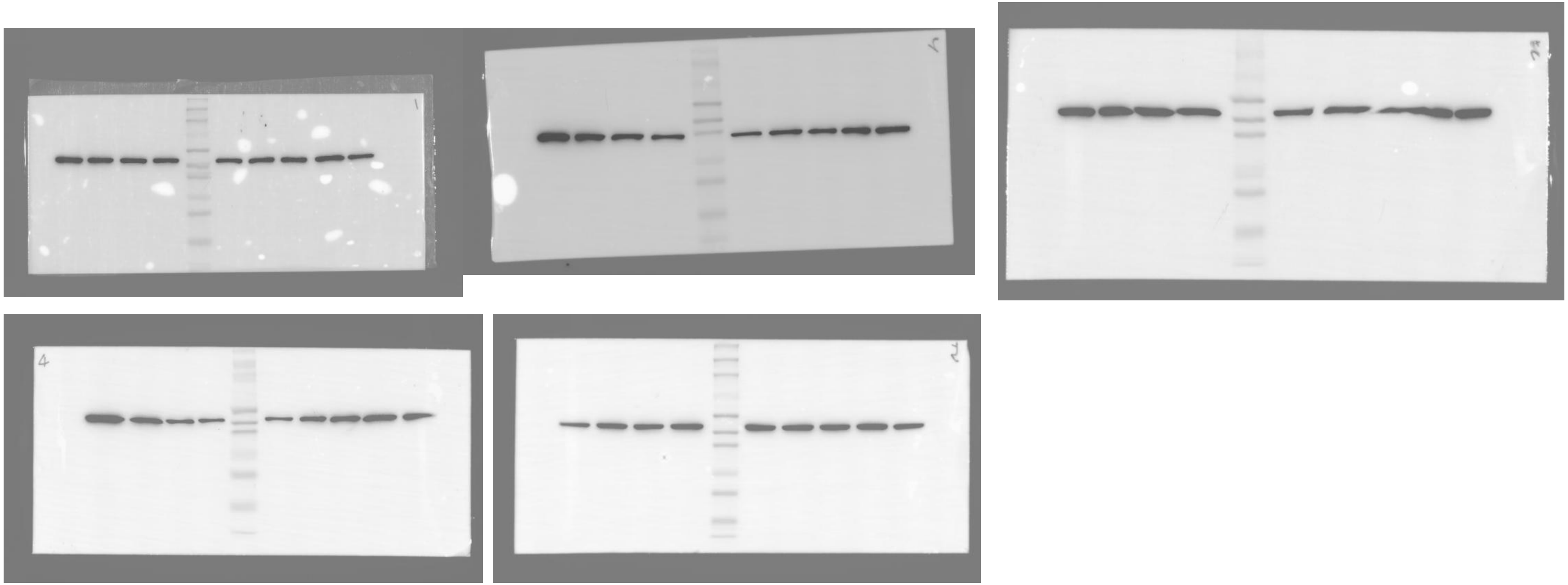

Supplement: Supplementary file 5 — WB original data [file 41413_2025_453_MOESM5_ESM.pdf]
