# Supplementary figures and images for: Chemotherapeutic drug-triggered AEP-cleaved G3BP1 orchestrates stress granules/nucleoli/mitochondria in osteosarcoma
Source: Bone Res. 2025 Aug 26;13:74. doi: 10.1038/s41413-025-00453-w (PMC12381239; doi:10.1038/s41413-025-00453-w)

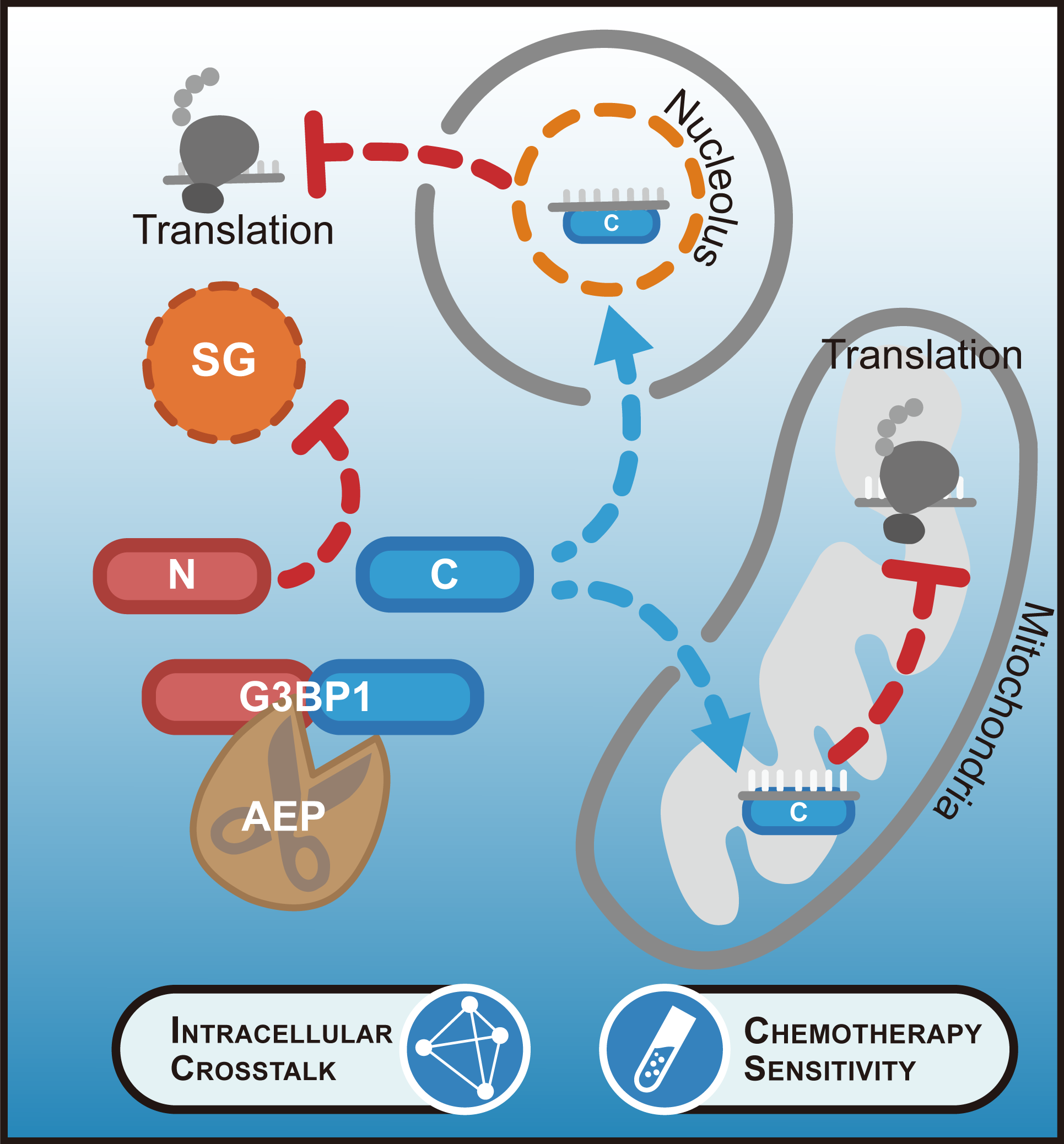

Supplement: Supplementary file 6 — Graphic Abstract [file 41413_2025_453_MOESM6_ESM.tif]
