# Supplementary material for: Chemotherapeutic drug-triggered AEP-cleaved G3BP1 orchestrates stress granules/nucleoli/mitochondria in osteosarcoma
Source: Bone Res. 2025 Aug 26;13:74. doi: 10.1038/s41413-025-00453-w (PMC12381239; doi:10.1038/s41413-025-00453-w)

细胞遗传质量鉴定检测

Cell Line Authentication Service

STR 基因型检测报告

样 品 信 息

样品编号：

客户样本编号	公司编号
U2OS	20221123

样品数量： 1

样品性状： 细胞系

检测项目： STR

检测方法： 用 Axygen 的基因组抽提试剂盒提取 DNA，采用 21- STR 扩增方案扩增，在 ABI 3730XL 型遗传分析仪上对 STR 位点和性别基因 Amelogenin 进行检测。

检 测 结 果

(一) 检验基本情况

公司编号	多等位基因	匹配细胞系	细胞库	EV 值	匹配说明
20221123	无	U2OS	ATCC	1.0	完全匹配

样本基因型检验结果

- 多等位基因指三等位及以上基因现象。
- 本次检测各细胞分型结果良好。

(二) 各样本描述

- 20221123：该株细胞 DNA 分型在细胞系检索中找到**完全匹配**的细胞系，ATCC 数据库显示细胞名为 **U2OS**，细胞号对应 **CVCL_0042**。本次检测在该细胞系中**没有发现多等位基因，**。

  备注：待测细胞系与收录于 ATCC, DSMZ（DSMZ 收录了来自 ATCC、 DSMZ、JCRB 和 RIKEN 等 2490 株细胞的 STR 数据）, ExPASy 细胞库（ExPASy 收录了来自于 ATCC、DSMZ、JCRB、ECACC 和 Riken 等数据库约 8,000 株人源细胞 STR 数据）中的 STR 数据匹配，未收录于上述细胞库的细胞将无法匹配。根据 ATCC 标准委员会鉴定标准（ANSI/ATCC ASN-0002-2022）,匹配度 EV≥80%认为它们具有相关性，可能衍生于共同的祖先细胞；匹配度 55%-80%之间，需要结合其它方法进一步的鉴定认证其相关性。

（三）样本分型结果

细胞的 STR 位点和 Amelogenin 位点的基因分型结果							
Loci	送检细胞 STR 信息				细胞库细胞 STR 信息		
	送检细胞名：U2OS				细胞库细胞名： U2OS		
	Allele1	Allele2	Allele3	Allele4	Allele1	Allele2	Allele3
D5S818	11	11			11	11	
D13S317	13	13			13	13	
D7S820	11	12			11	12	
D16S539	11	12			11	12	
VWA	14	18			14	18	
TH01	6	9.3			6	9.3	
AMEL	X	X			X	X	
TPOX	11	12			11	12	
CSF1PO	13	13			13	13	
D12S391	19	20					
FGA	20	20					
D2S1338	20	24					
D21S11	31	31					
D18S51	14	14					
D8S1179	12	14					
D3S1358	16	16					
D6S1043	11	11					
PENTAE	10	13					
D19S433	15	15					
PENTAD	9	9					
D1S1656	16	17.3					

其 他 说 明

(一) 分型方案及位点分布

	方案 1	方案 2	方案 3	方案 4
1	D3S1358	D8S1179	D19S433	AMEL
2	VWA	D21S11	TH01	D1S1656
3	D7S820	D16S539	D13S317	D5S818
4	CSF1PO	D2S1338	TPOX	D12S391
5	PENTAE	PENTAD	D18S51	FGA
6			D6S1043	

实验方案及位点

(二) STR 数据库比对

本公司采用 DSMZ tools 进行细胞系比对，其中包含来自于 ATCC, DSMZ, JCRB 和 RIKEN 数据库的 2455 个细胞系 STR 数据。如果待检测细胞未收录于以上细胞库或这是自行建立的新细胞系将无法进行比对，用户需根据细胞分型结果自行与其他数据库进行比对。

Sample File	Sample Name	Panel	OS	SQ
44_D06_Cellidentification-2-1124.fsa	U2OS	21Plex_STR_Panel_v1.1		

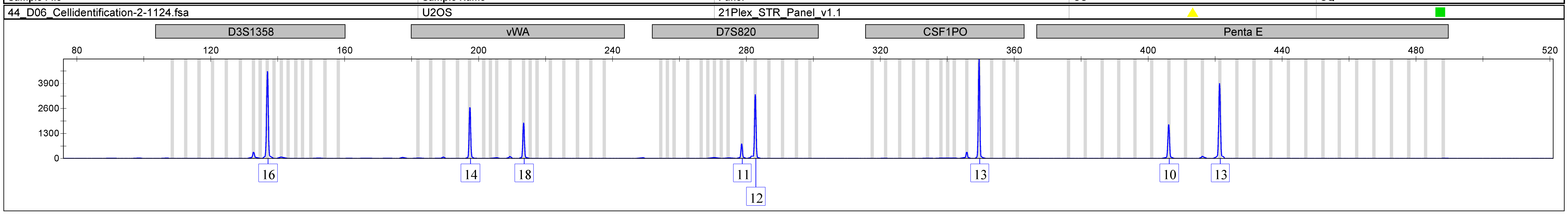

44_D06_Cellidentification-2-1124.fsa	U2OS	21Plex_STR_Panel_v1.1		

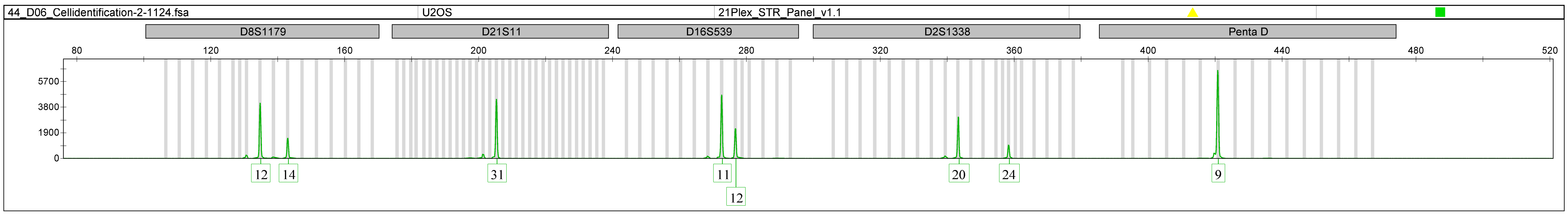

44_D06_Cellidentification-2-1124.fsa	U2OS	21Plex_STR_Panel_v1.1		

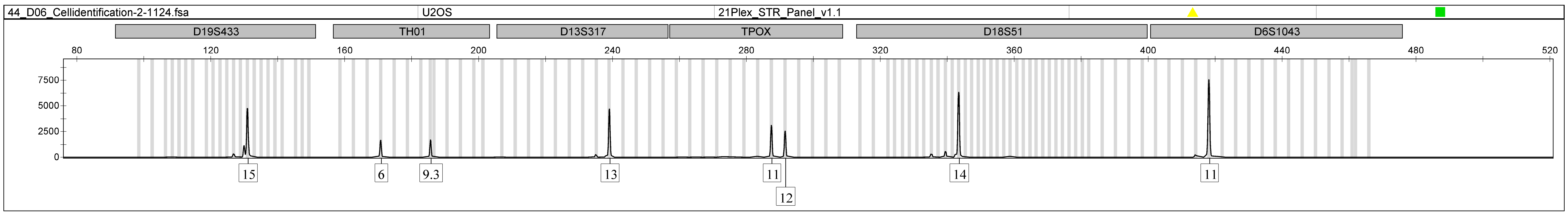

44_D06_Cellidentification-2-1124.fsa	U2OS	21Plex_STR_Panel_v1.1		

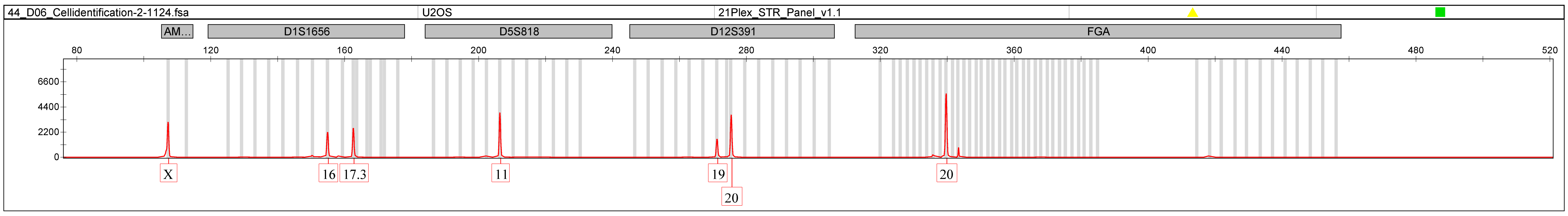

Supplement: Supplementary file 7 — U2OS STR [file 41413_2025_453_MOESM7_ESM.pdf]
