# Supplementary material for: Chemotherapeutic drug-triggered AEP-cleaved G3BP1 orchestrates stress granules/nucleoli/mitochondria in osteosarcoma
Source: Bone Res. 2025 Aug 26;13:74. doi: 10.1038/s41413-025-00453-w (PMC12381239; doi:10.1038/s41413-025-00453-w)

细胞遗传质量鉴定检测

Cell Line Authentication Service

STR 基因型检测报告

样 品 信 息

样品编号：

客户样本编号	公司编号
143B	20250408-01

样品数量： 1

样品性状： 细胞系

检测项目： STR

送检单位： **上海富衡生物科技有限公司**

检测方法： 用 Axygen 的基因组抽提试剂盒提取 DNA，采用 21- STR 扩增方案扩增，在 ABI 3730XL 型遗传分析仪上对 STR 位点和性别基因 Amelogenin 进行检测。

检 测 结 果

(一)　检验基本情况

公司编号	多等位基因	匹配细胞系	细胞库	EV 值	匹配说明
20250408-01	无	143B	DSMZ	1.0	完全匹配

样本基因型检验结果

- 多等位基因指三等位及以上基因现象。
- 本次检测各细胞分型结果良好。

(二)　各样本描述

- 20250408-01：该株细胞 DNA 分型在细胞系检索中找到**完全匹配**的细胞系，DSMZ 数据库显示细胞名为 **143B**，细胞号对应 **CRL-8303**。本次检测在该细胞系中**没有发现多等位基因**。

EV	Cell No.	Cell name	Locus names								
			D5S818	D13S317	D7S820	D16S539	VWA	TH01	AM	TPOX	CSF1PO
	Query (Your Cell)		13,13	12,12	11,12	10,13	18,18	6,6	X,X	11,11	12,12
1.0(36/36)	CRL-8303	143B	['13', '13']	['12', '12']	['11', '12']	['10', '13']	['18', '18']	['6', '6']	['X', 'X']	['11', '11']	['12', '12']

备注：待测细胞系与收录于 ATCC, DSMZ（DSMZ 收录了来自 ATCC、 DSMZ、JCRB 和 RIKEN 等 2490 株细胞的 STR 数据）, ExPASy 细胞库（ExPASy 收录了来自于 ATCC、DSMZ、JCRB、ECACC 和 Riken 等数据库约 8,000 株人源细胞 STR 数据）中的 STR 数据匹配，未收录于上述细胞库的细胞将无法匹配。根据 ATCC 标准委员会鉴定标准（ANSI/ATCC ASN-0002-2022）,匹配度 EV≥80%认为它们具有相关性，可能衍生于共同的祖先细胞；匹配度 55%-80%之间，需要结合其它方法进一步的鉴定认证其相关性。

（三）样本分型结果

细胞的 STR 位点和 Amelogenin 位点的基因分型结果						
Loci	送检细胞 STR 信息			细胞库细胞 STR 信息		
	送检细胞名：143B			细胞库细胞名：143B		
	Allele1	Allele2	Allele3	Allele1	Allele2	Allele3
D5S818	13	13		13	13	
D13S317	12	12		12	12	
D7S820	11	12		11	12	
D16S539	10	13		10	13	
VWA	18	18		18	18	
TH01	6	6		6	6	
AMEL	X	X		X	X	
TPOX	11	11		11	11	
CSF1PO	12	12		12	12	
D12S391	20	21				
FGA	24	24				
D2S1338	24	25				
D21S11	31.2	32.2				
D18S51	17	17				
D8S1179	11	14				
D3S1358	15	15				
D6S1043	18	18				
PENTAE	7	12				
D19S433	13	13				
PENTAD	9	10				
D1S1656	13	15				

其 他 说 明

(一)　分型方案及位点分布

	方案 1	方案 2	方案 3	方案 4
1	D3S1358	D8S1179	D19S433	AMEL
2	VWA	D21S11	TH01	D1S1656
3	D7S820	D16S539	D13S317	D5S818
4	CSF1PO	D2S1338	TPOX	D12S391
5	PENTAE	PENTAD	D18S51	FGA
6			D6S1043	

实验方案及位点

(二)　STR 数据库比对

如果待检测细胞未收录于国际、国内细胞库或者是自行建立的新细胞系将无法进行比对，用户需根据细胞分型结果自行与其对照参考数据进行比对，或者提供给我们协助分析。

签发日期：2025-04-10

Sample File	Sample Name	Panel	OS	SQ
31_G04_Cellidentification-2----0409.fsa	143B	21Plex_STR_Panel_v1.2		

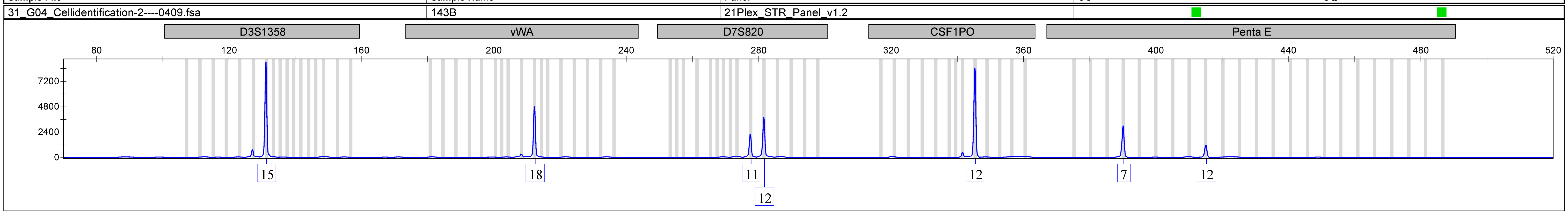

Sample File	Sample Name	Panel	OS	SQ
31_G04_Cellidentification-2----0409.fsa	143B	21Plex_STR_Panel_v1.2		

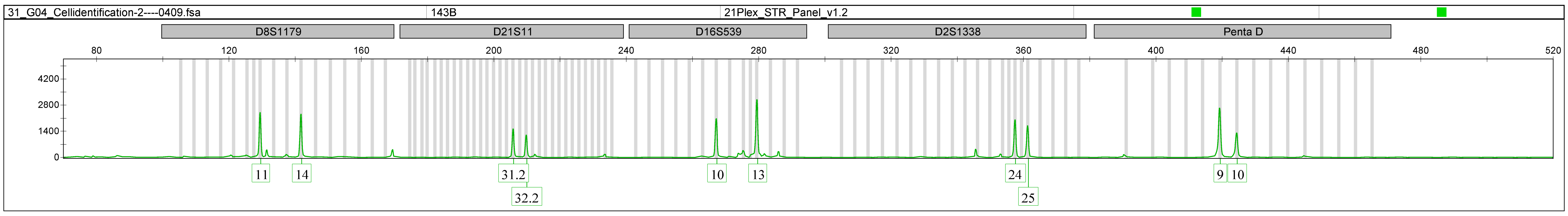

Sample File	Sample Name	Panel	OS	SQ
31_G04_Cellidentification-2----0409.fsa	143B	21Plex_STR_Panel_v1.2		

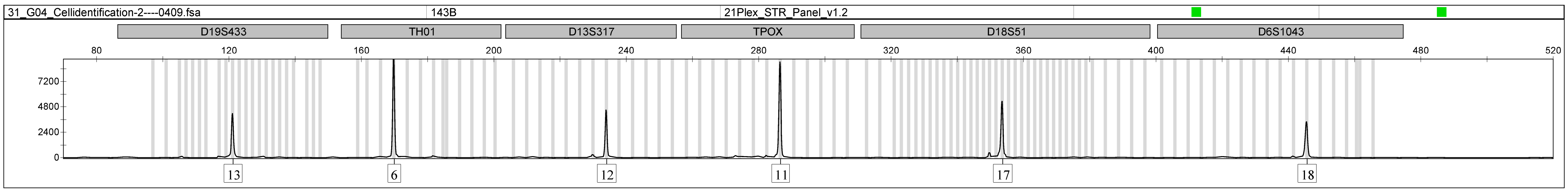

Sample File	Sample Name	Panel	OS	SQ
31_G04_Cellidentification-2----0409.fsa	143B	21Plex_STR_Panel_v1.2		

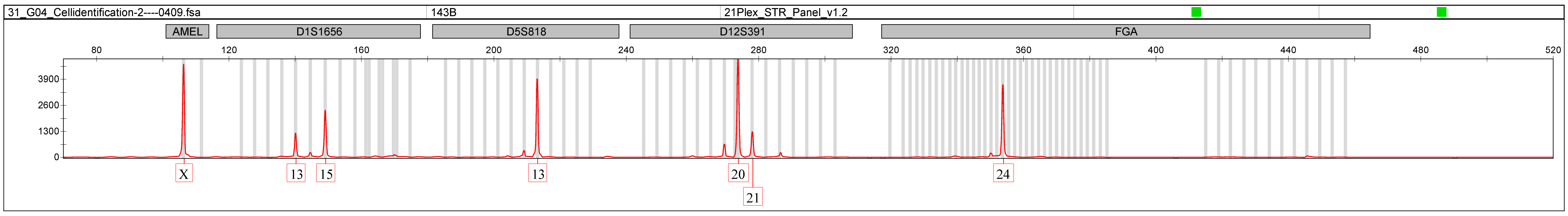

Supplement: Supplementary file 8 — 143B STR [file 41413_2025_453_MOESM8_ESM.pdf]
