# Supplementary material for: Chemotherapeutic drug-triggered AEP-cleaved G3BP1 orchestrates stress granules/nucleoli/mitochondria in osteosarcoma
Source: Bone Res. 2025 Aug 26;13:74. doi: 10.1038/s41413-025-00453-w (PMC12381239; doi:10.1038/s41413-025-00453-w)

Cell Line Authentication – STR Profiling

Sample from: FuHeng Cell Center, Shanghai, China
Testing Method: STR Genotyping
Report Time:Jan 19,2022

Cell Line Authentication - STR Profiling Report

Sample code

Table 1. Sample Code

Customer's code	Company Code
U87	20220117-07

Sample Number:1

Sample Type: Cell line

Testing Type: STR

Sample From: FuHeng Cell Center, Shanghai, China

Testing Method:

DNA was extracted by a commercial kit from CORNING (AP-EMN-BL-GDNA-250G). The twenty STRs including Amelogenin locus were amplified by six multiplex PCR and separated on ABI 3730XL Genetic Analyzer. The signals were then analyzed by the software GeneMapper.

Data Interpretation:

Cell lines were authenticated using Short Tandem Repeat (STR) analysis as described in 2012 in ANSI Standard (ASN-0002) by the ATCC Standards Development Organization (SDO) and inCapes-Davis et al., Match criteria for human cell line authentication: Where do we draw the line?

Int J Cancer.2013;132(11):2510-9.

Test Results:

1. Result

Table 2. Matching information on the cell lines

Sample Code	Multi-allele	Cell line matched	Cell Bank	Percentage
20220117-07	NO	U-87MG	DSMZ	1.0

Multi-allele means some STR contain more than two loci.

2. Sample Description

20220117-07 The DNA typing of the cell line found a completely matched cell line in the cell line search. The **DSMZ** database showed that the cell name was **U-87mg** and the cell number corresponded to **HTB-14**. No multiple alleles were found in this cell line

3. Genotyping Result

STR and Amelogenin Genotyping Results of Cell line						
Loci	**Sample**			**Cell Bank information**		
	U87			U-87MG		
	Allele1	Allele2	Allele3	Allele1	Allele2	Allele3
D5S818	11	12		11	12	
D13S317	8	11		8	11	
D7S820	8	9		8	9	
D16S539	12	12		12	12	
VWA	15	17		15	17	
TH01	9.3	9.3		9.3	9.3	
AMEL	X	X		X	X	
TPOX	8	8		8	8	
CSF1PO	10	11		10	11	
D12S391	18	21				
FGA	18	24				
D2S1338	20	23				
D21S11	28	32.2				

D18S51	13	14				
D8S1179	10	11				
D3S1358	16	17				
D6S1043	11	18				
PENTAE	7	14				
D19S433	15	15.2				
PENTAD	9	14				
D1S1656	15	15				

Others

1. Genotyping Strategy and Site DistributionAttached Table. Experimental Strategy and Sites

	Strategy 1	Strategy 2	Strategy 3	Strategy 4
1	TH01	TPOX	D3S1358	AMEL
2	D12S391	VWA	D13S317	D5S818
3	D7S820	D8S1179	D6S1043	D2S1338
4	CSF1PO	PENTAD	D16S539	D21S11
5	FGA		D19S433	D18S51
6	PENTAE			

The allele match algorithm compares the 8 core loci plus amelogenin only, even though alleles from all loci will be reported when available.

2. STR database comparison

DSMZ tools was used to carry on the cell line comparison, which contains 2455 cell lines STR data from ATCC, DSMZ, JCRB ,ECACC and RIKEN databases. If the cell is not included in the above cell library, users need to compared with other databases.

Applied Biosystems

GeneMapper ID v3.2

20220117

Sample File	Sample Name	Panel	SQO	OS	SQ
14_F02_CellLineAuthentication-1-0118.fsa	U87	21Plex_STR_Panel_v1.1			

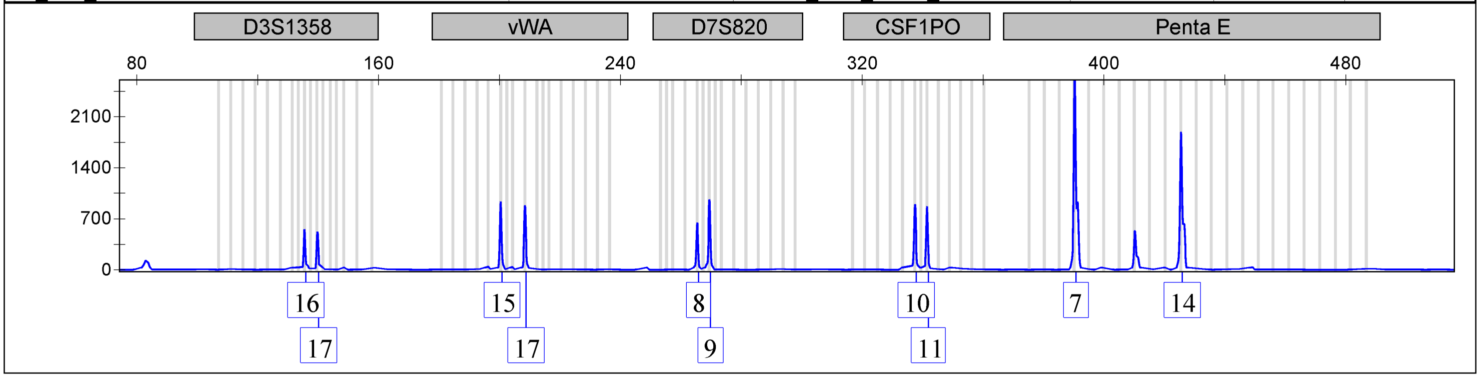

14_F02_CellLineAuthentication-1-0118.fsa	U87	21Plex_STR_Panel_v1.1			

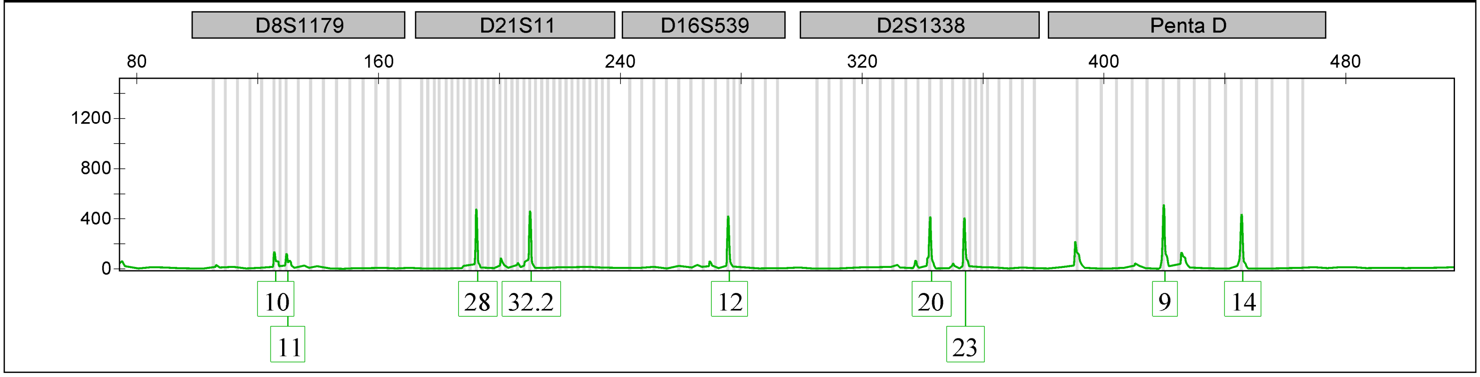

14_F02_CellLineAuthentication-1-0118.fsa	U87	21Plex_STR_Panel_v1.1			

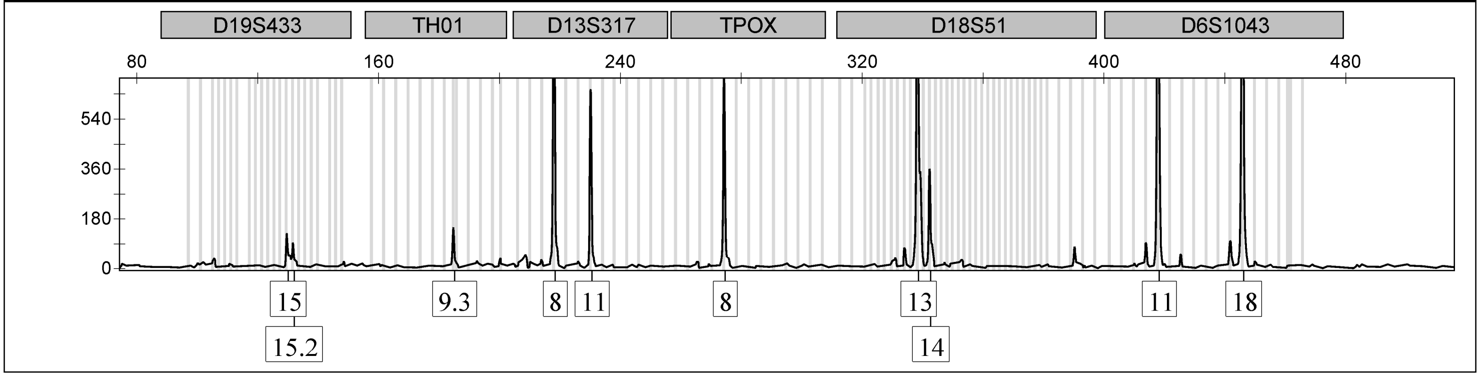

14_F02_CellLineAuthentication-1-0118.fsa	U87	21Plex_STR_Panel_v1.1			

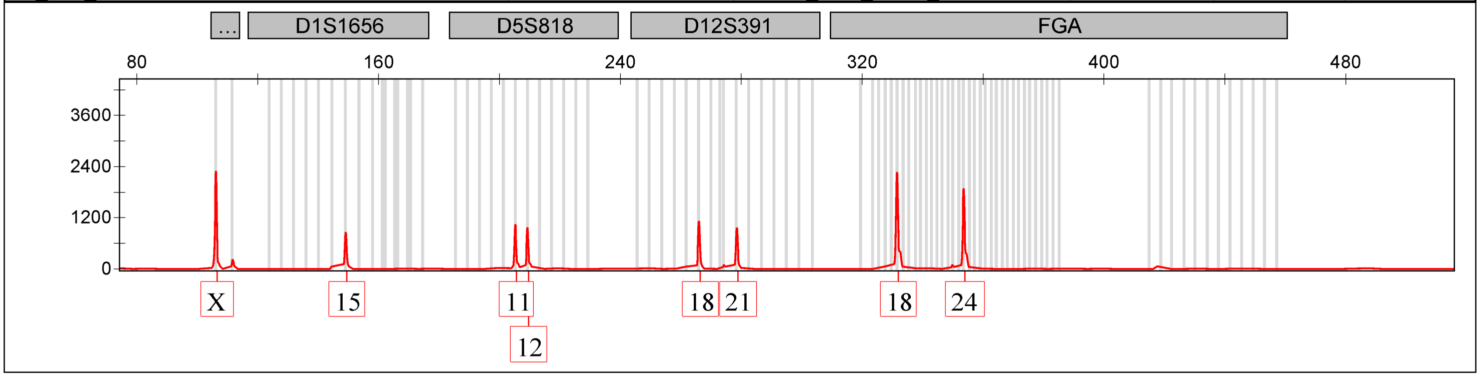

Supplement: Supplementary file 9 — U87 STR [file 41413_2025_453_MOESM9_ESM.pdf]
