# Supplementary material for: Chemotherapeutic drug-triggered AEP-cleaved G3BP1 orchestrates stress granules/nucleoli/mitochondria in osteosarcoma
Source: Bone Res. 2025 Aug 26;13:74. doi: 10.1038/s41413-025-00453-w (PMC12381239; doi:10.1038/s41413-025-00453-w)

细胞遗传质量鉴定检测

Cell Line Authentication Service

STR 基因型检测报告

样 品 信 息

样品编号：

客户样本编号	公司编号
A549	20210510-07

样品数量： 1

样品性状： 细胞系

检测项目： STR

送检单位：富衡

检测方法：用 Axygen 的基因组抽提试剂盒提取 DNA，采用 21- STR 扩增方案扩增，在 ABI 3730XL 型遗传分析仪上对 STR 位点和性别基因 Amelogenin 进行检测。

检 测 结 果

(一) 检验基本情况

公司编号	多等位基因	匹配细胞系	细胞库	EV 值	匹配说明
20210510-07	有	A-549	DSMZ	1.0	完全匹配

样本基因型检验结果

- 多等位基因指三等位及以上基因现象。
- 本次检测各细胞分型结果良好。
- **各样本描述**
- 20210510-07：该株细胞 DNA 分型在细胞系检索中找到**完全匹配**的细胞系，DSMZ 数据库显示细胞名为 **A-549**，细胞号对应 **107**。本次检测在该细胞系中**发现多等位基因**。

备注：待测细胞系与收录于 ATCC, DSMZ, JCRB 和 RIKEN 数据库的细胞系 STR 数据进行比对，未收录于以上细胞库的细胞系将无法匹配。

样本分型结果

细胞的 STR 位点和 Amelogenin 位点的基因分型结果						
Loci	送检细胞 STR 信息			细胞库细胞 STR 信息		
	送检细胞名：A549			细胞库细胞名：A-549		
	Allele1	Allele2	Allele3	Allele1	Allele2	Allele3
D5S818	11	11		11	11	

D13S317	11	11		11	11	
D7S820	8	11		8	11	
D16S539	11	12		11	12	
VWA	14	14		14	14	
TH01	8	9.3		8	9.3	
AMEL	X	Y		X	Y	
TPOX	8	11		8	11	
CSF1PO	10	12		10	12	
D12S391	18	18				
FGA	23	23				
D2S1338	24	24				
D21S11	29	29				
D18S51	14	17				
D8S1179	13	14				
D3S1358	16	16				
D6S1043	10	11	13			
PENTAE	7	11				
D19S433	13	13				
PENTAD	7	9				
D1S1656	17	18.3				

其 他 说 明

(一) 分型方案及位点分布

	方案 1	方案 2	方案 3	方案 4
1	D3S1358	D8S1179	D19S433	AMEL
2	VWA	D21S11	TH01	D1S1656
3	D7S820	D16S539	D13S317	D5S818
4	CSF1PO	D2S1338	TPOX	D12S391

5	PENTAE	PENTAD	D18S51	FGA
6			D6S1043	

实验方案及位点

(二) STR 数据库比对

本公司采用 DSMZ tools 进行细胞系比对，其中包含来自于 ATCC, DSMZ, JCRB 和 RIKEN 数据库的 2455 个细胞系 STR 数据。如果待检测细胞未收录于以上细胞库或这是自行建立的新细胞系将无法进行比对，用户需根据细胞分型结果自行与其他数据库进行比对。

Sample File	Sample Name	Panel	SQO	OS	SQ
47_G06_CellLineAuthehntication-1-0516.fsa	A549	21Plex_STR_Panel_v0.4			

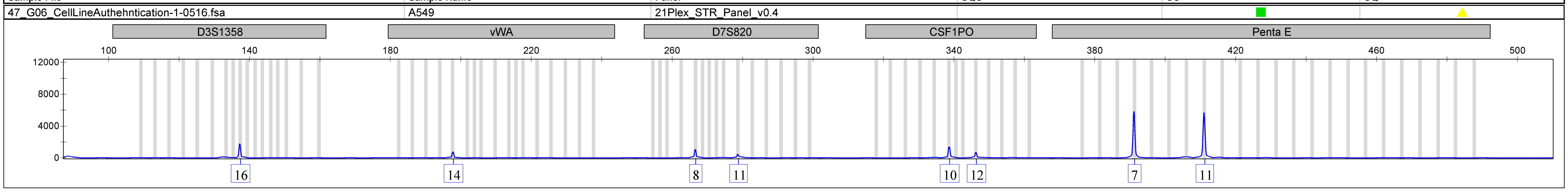

47_G06_CellLineAuthehntication-1-0516.fsa	A549	21Plex_STR_Panel_v0.4			

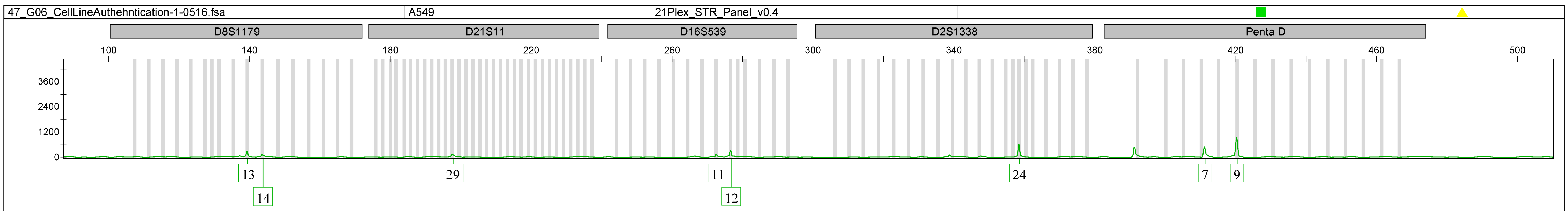

47_G06_CellLineAuthehntication-1-0516.fsa	A549	21Plex_STR_Panel_v0.4			

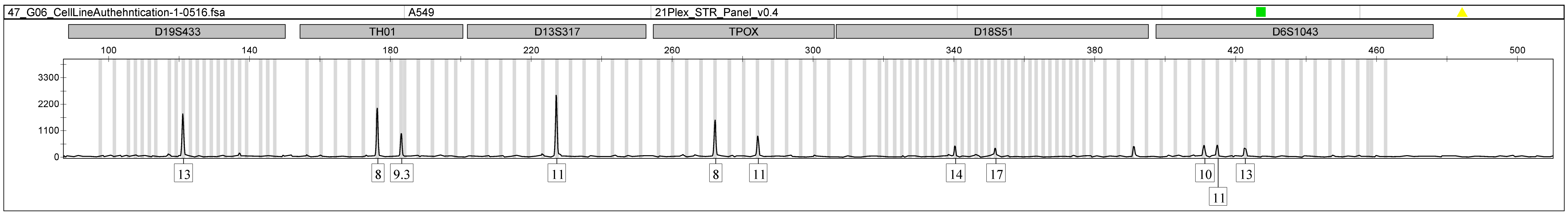

47_G06_CellLineAuthehntication-1-0516.fsa	A549	21Plex_STR_Panel_v0.4			

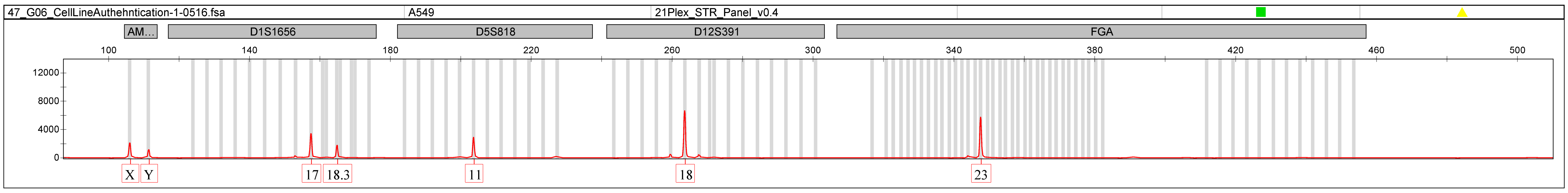

Supplement: Supplementary file 10 — A549 STR [file 41413_2025_453_MOESM10_ESM.pdf]
